# Peptide modification *via* a mild carbonylative Suzuki–Miyaura reaction gives late-stage access to diaryl-ketones

**DOI:** 10.1039/d5sc05588a

**Published:** 2026-05-05

**Authors:** James P. Kleppen, Neil W. J. Scott, Tom J. Phillips, Ksenia S. Stankevich, James R. Donald, Lydia J. Barber, Katie M. Lewis, Ian J. S. Fairlamb, Christopher D. Spicer

**Affiliations:** a Department of Chemistry, University of York Heslington YO10 5DD UK chris.spicer@york.ac.uk; b York Biomedical Research Institute, University of York Heslington YO10 5DD UK; c Department of Biology, University of York Heslington YO10 5DD UK

## Abstract

Benzophenones and other diaryl ketones are common handles for photo-crosslinking and photo-affinity labelling. Here, we introduce the use of an operationally simple carbonylative Suzuki–Miyaura cross-coupling for the late-stage introduction of these handles into unprotected peptides. By using molybdenum hexacarbonyl, Mo(CO)_6_, as a carbon monoxide surrogate, these reactions take place under mild aqueous conditions, under an open (air) atmosphere, requiring no specialist equipment or techniques.

## Abstract 2

Benzophenones can be chemically installed into peptides *via* a carbonylative Suzuki–Miyaura reaction.

## Graphical abstract

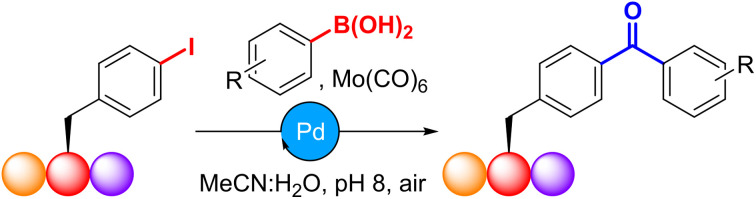

Diaryl-ketones, typified most commonly by benzophenones, are an important molecular handle for biomedical research. Upon irradiation with UV light, these ketones are activated to form a highly reactive ‘C–O’ triplet diradical species, which can react with nearby biomolecules *via* a sequential abstraction–recombination process.^1,2^ Diaryl-ketones are therefore important reagents for photo-crosslinking and photo-affinity labelling, techniques which covalently trap interacting biomolecules, and allow the investigation of important processes in health and disease.^3^

In this context, benzophenone-containing peptides have proved invaluable in the study of diverse biological processes, ranging from protein-membrane interactions^4^ to post-translational modification.^5^ In almost all cases, the benzophenone motif is integrated into the peptide either by amide coupling at the ε-amine of lysine residues, or through the incorporation of the unnatural amino acid *p*-benzoyl-l-phenylalanine (Bpa) during solid phase peptide synthesis (SPPS).^6^ However, the photo-labelling efficiency of Bpa is often poor, due to its low absorptivity and quantum yield.^7^ Diversification of the diaryl-ketone motif can greatly increase this photo-labelling efficiency, while also offering potential routes to red-shift absorbance away from the damaging UV region and generate photo-orthogonal crosslinkers for multiplexed labelling.^8^ Indeed, the effects of aryl substituents on diaryl ketone photophysical properties have been extensively studied,^9^ with Joiner *et al.* having demonstrated up to 50-fold enhancements in protein crosslinking using halogenated analogues of Bpa.^7^

This need for diversification requires the parallel synthesis of large numbers of diaryl-ketone containing peptides, with associated challenges in the synthesis and protection of diaryl-ketone based amino acids for SPPS. In contrast, a chemical strategy for late-stage installation of diaryl-ketones into unprotected peptides would be a more powerful and versatile tool for the biological and biomedical communities. However, to be viable, such a strategy should be efficient at ambient temperatures, near neutral pH, and in aqueous media. It would also need to be able to tolerate the myriad of reactive functional groups typically found in peptides. Finally, the reaction would benefit from being operational in an open atmosphere to maximise translatability to non-specialist labs and to facilitate small-scale reactions.

The development of such a strategy therefore represents a significant challenge, that has yet to be addressed. Indeed, these requirements rule out conventional synthetic routes towards diaryl-ketones, such as Friedel–Crafts acylations or carbonyl addition/oxidation reactions.^9,10^ While carbonylative Suzuki–Miyaura cross couplings (cSMCCs) provide a promising alternative, these reactions commonly require the use of highly toxic and flammable CO.^11^ This can be limiting to the use of these reactions in general synthesis, a factor that is exacerbated for bioconjugation. There is therefore great interest in the use of CO-surrogates and CO delivery vehicles, including transition metal carbonyls, for cSMCC.^12^ Indeed, in 2019 Sun *et al.* reported the use of Mo(CO)_6_ and palladium(ii) trifluoroacetate, ‘Pd(TFA)_2_’, as a precatalyst for the cSMCC of aryl iodides in acetonitrile : water mixtures at 50 °C.^13^ Building on our previous experiences of translating Pd-mediated cross-couplings to peptide and protein substrates, we were drawn to the possibilities of this approach in bioconjugation.^14–18^

In this paper, we report the development of operationally simple carbonylative Suzuki–Miyaura cross-couplings (cSMCCs) for peptide modification (Fig. 1). By using Mo(CO)_6_ as a carbon monoxide surrogate, these couplings take place at < 37 °C, under aqueous, ‘open-to-air’ conditions, and are therefore applicable to fully deprotected substrates. This has allowed us to demonstrate the selective late-stage formation of structurally and chemically diverse diaryl-ketones, for future applications in photo-labelling studies.

**Fig. 1:**
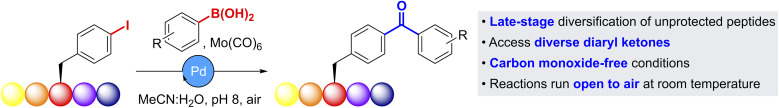
Overview of the carbonylative Suzuki–Miyaura cross-coupling (cSMCC) developed in this paper for the late-stage diversification of unprotected peptides.

## Results and discussion

**Scheme 1:**
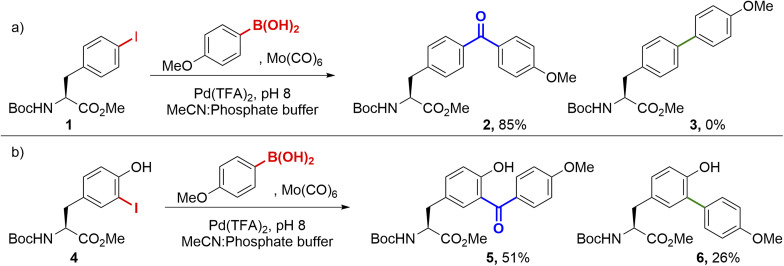
Model cSMCCs on protected *p*IPhe and 3-ITyr substrates. Both reactions were undertaken ‘open-to-air’ at room temperature, with no attempt to exclude oxygen. Reaction conditions: Mo(CO)_6_ (0.5 equiv.), Pd(TFA)_2_ (0.05 equiv.), boronic acid (1.5 equiv.), acetonitrile : PBS (pH 8, 5 : 1), rt, 20 h.

We commenced our studies by investigating cSMCCs on the unnatural aryl iodide amino acid *para*-iodophenylalanine (*p*IPhe). *p*IPhe can be site-selectively incorporated into proteins *via* codon suppression, where we have previously shown it can act as a handle for Suzuki–Miyaura modification and the mimicry of natural post-translational modifications.^14–16,19^ Couplings were initially performed on Boc-*p*IPhe-OMe 1, in the presence of 0.5 equiv. Mo(CO)_6_, 5 mol% ‘Pd(TFA)_2_’, and 1.5 equiv. 4-methoxyphenyl boronic acid. Under these conditions, the carbonylative product 2 was preferentially delivered over the non-carbonylative side-product 3. Notably, couplings proceeded readily at room temperature and in pH 8.0 phosphate buffer (Scheme 1a). Importantly, when no effort was made to degas the reagents or to ensure an inert atmosphere, little difference in conversion with respect to the starting material was observed. Analogous results were found for the modification of Boc-3-iodotyrosine-OMe 4, another protein-compatible iodinated amino acid^20^ that also plays a key role in human biology as an intermediate during thyroid hormone synthesis.^21^ Again, cSMCC product 5 dominated (51% yield), though the non-carbonylative side-product 6 also formed in significant amounts (26% yield, Scheme 1b).

**Fig. 2:**
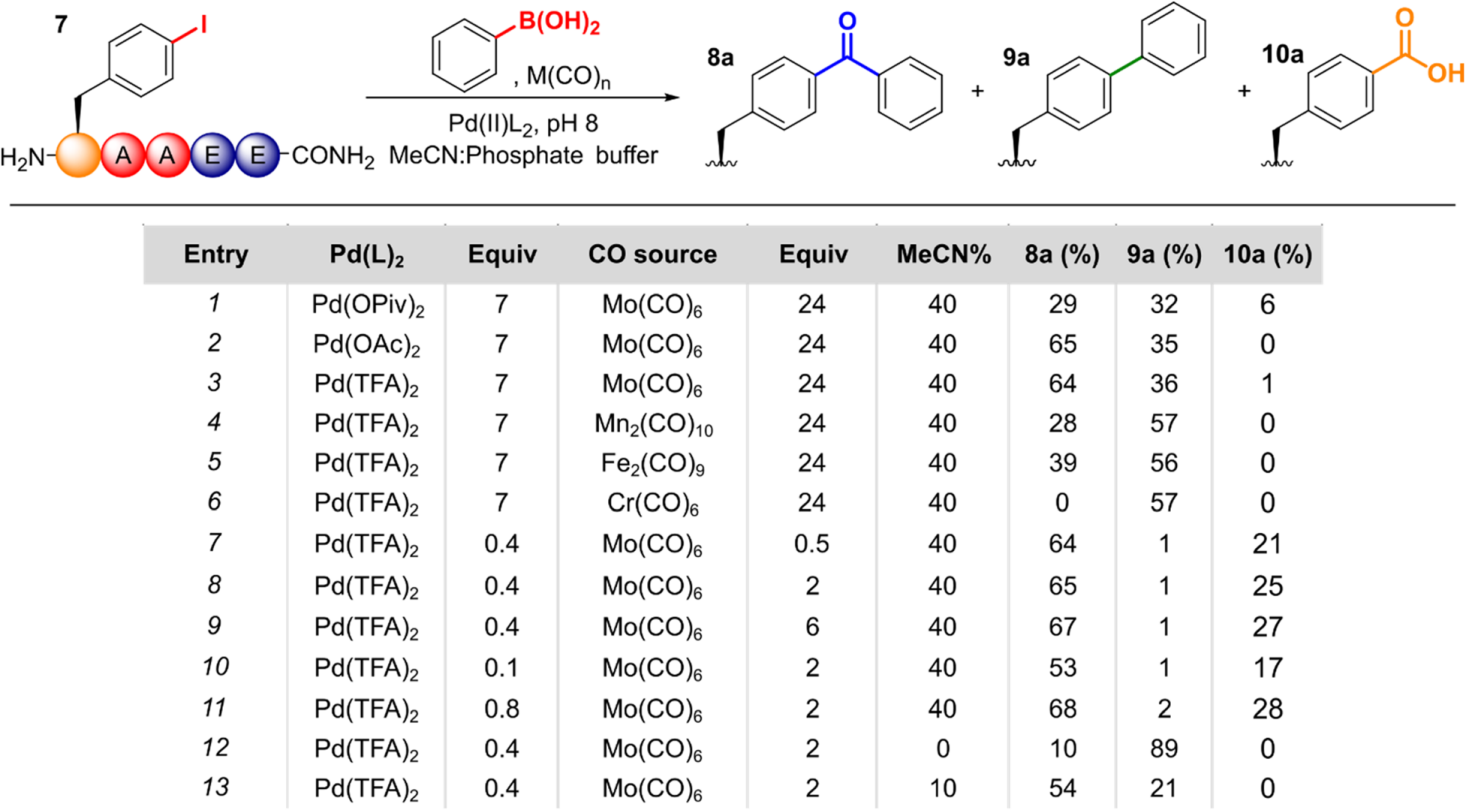
Optimisation of cSMCC on the model peptide *p*IPhe-AAEE, 7.

With these promising results in hand, we applied the cSMCC reaction conditions to a model iodinated peptide, H_2_N-*p*IPhe-AAEE-CONH_2_, 7, using phenylboronic acid as a coupling partner. Pleasingly, conversion to the cSMCC product 8a was observed by LC-MS, with the desired structure confirmed by ^1^H NMR (see SI. Fig. S2) and comparison to an equivalent peptide synthesised *via* SPPS using Bpa directly. Further evidence for the presence of the diaryl ketone was provided by reduction to the corresponding alcohol upon treatment with sodium borohydride (See SI Section 5.12). We therefore set out to screen and optimise the reaction conditions for peptide modification (Fig. 2).

Under the conditions of the reaction, there are three possible pathways that can operate following initial oxidative addition of the aryl halide (see SI Fig. S1 for a depiction of the catalytic cycle): (i) when CO insertion is faster than transmetallation of the boronic acid, the desired cSMCC product 8a can be formed; (ii) if transmetallation is faster than CO insertion, then the corresponding non-carbonylated Suzuki–Miyaura product 9a will be formed; and (iii) in the presence of a nucleophile, in this case water, an acylpalladium–hydroxo complex may form which can ultimately lead to the corresponding carboxylic acid product, 10a.^22,23^ Therefore, the relative conversions to these two by-products was monitored *via* LC-MS alongside the desired diaryl-ketone.

First, we screened alternative palladium precatalysts. ‘Pd(TFA)_2_’, ‘Pd(OAc)_2_’, and water soluble 2-amino-4,6-dihydroxypyrimidine (ADHP)-Pd complex^14,24^ showed comparable activity. In contrast, palladium(ii) pivalate, ‘Pd(OPiv)_2_’, gave significantly lower conversion (Entries 1–3, see SI Section 5.1 for further details). Of the metal carbonyl complexes screened, Mo(CO)_6_ gave the highest conversion to 8a, with both Mn_2_(CO)_10_ and Fe_2_(CO)_9_ giving lower cSMCC : SMCC ratios, and Cr(CO)_6_ giving solely the SMCC product (Entries 4–6, SI Section 5.2). In the absence of a metal carbonyl complex, 9a was formed exclusively.

Following this initial validation, we aimed to optimise the reagent loadings. Formation of the cSMCC product 8a was found to be consistent across a range of 0.5–24 equiv. of Mo(CO)_6_ with a significant drop-off at lower loadings (Entries 7–9, SI Section 5.3). Similarly, Pd loadings could be lowered to as little as 10 mol% (Entry 10, SI Section 5.4). However, at such small loadings of catalyst, the practical challenges of measuring such small quantities led to high variability in reaction efficiency, and so a loading of 40 mol% was used for further reactions. Although these loadings appear high, at the small scales required for peptide modification they equate to ∼2.5 mg of precatalyst for preparative-scale peptide modification, as discussed later. The effect of lowering the arylboronic acid equivalents was more impactful. At low loadings, 4-carboxy peptide 10a became the dominant product, as would be expected when less boronic acid was available for transmetallation of the carbonylated-Pd complex (See SI Fig. S1). Up to 8 equiv. of boronic acid, a steady increase in the amount of 8a formed, above which further increases had minimal effect on conversion (SI Section 5.5).

We next sought to reduce the amount of acetonitrile required for effective cSMCC. Though acetonitrile is tolerated at low concentrations by many biomolecules,^25^ at higher concentrations it can disrupt folding within peptides and proteins.^26^ We observed little difference in cSMCC efficiency or selectivity over a range of 20–50% acetonitrile in PBS. At 10% acetonitrile, a small decrease in both conversion and selectivity for the cSMCC product was observed. However, even under these stringent conditions, 8a was still the major product (54% relative conversion, Entry 13). In the absence of acetonitrile, the non-carbonylative product 9a was the dominant species (Entry 12). The reaction was also tolerant of a number of buffers, with pH 8 potassium phosphate buffer being found to be optimal (SI Section 5.8).

We next performed a screen to identify an optimal temperature for the reaction. Conversion was seen at as little as 5 °C, though a marked increase in the formation of 8a was observed above 25 °C (SI Section 5.9). Notably, above 40 °C conversion to the non-carbonylated SMCC side-product 9a appeared to be enhanced, and so a temperature of 37 °C was chosen to move forwards. At this temperature, a time-screen indicated that the reaction neared completion after ∼4 h, and so reactions were typically left to progress overnight (SI Section 5.10). Under these optimised conditions, peptides containing *para*-bromophenylalanine residues were unreactive, with no evidence of any consumption of the starting peptide.

Cumulatively, these results indicate that under the conditions of the reaction, at weakly basic pH and with aryl iodide coupling partners, CO insertion occurs at a faster rate than transmetallation. However, transmetallation remains faster than elimination from an acylpalladium–hydroxo complex which may form during the catalytic cycle from the corresponding halide complex, though this pathway was still found to be competitive under some conditions.^23^

The hypothesis that CO insertion does not rely on simple CO release from Mo(CO)_6_, is supported by a number of observations: (i) selectivity for the cSMCC *vs.* non-carbonylative product is consistent between a Mo : Pd ratio of ∼1 : 1–15 : 1, indicating that increasing CO concentration does not drive selectivity within this range – this observation is caveated by the low solubility of CO in water and the possibility that the catalyst may sequester CO, and so is not in isolation sufficient to support the hypothesis; (ii) when Mo(CO)_6_ was pre-incubated in acetonitrile : PBS (pH 8.0) for 30 min, prior to coupling, no change in conversion to 8a was observed; and (iii) when reactions were performed in an open flask no differences in selectivity were found. Together, these results indicate that direct acetonitrile-induced CO release from the Mo(CO)_6_ complex does not play a role in the coupling, while similar selectivities observed when reactions were run in the dark also rule out a photo-activated process of CO release.^27^ This is in line with the findings of Sun *et al.*, who proposed that acetonitrile plays a key role in activating the Mo(CO)_6_ for cSMCC, though the exact mechanism by which this takes place is not clear – in their work, replacing Mo(CO)_6_ with a CO atmosphere failed to deliver any product, suggesting induced CO release is not a sole contributing factor, though they did observe some degree of cSMCC at elevated pressures. Our observation that the non-carbonylated product 9a dominated in the absence of acetonitrile further supports this idea. We have recently reported the formation of Pd–Fe nanocatalysts as a key species during cSMCCs in organic solvents, and it is possible that a related Pd–Mo species may play an important role in the couplings reported here.^28^

**Fig. 3:**
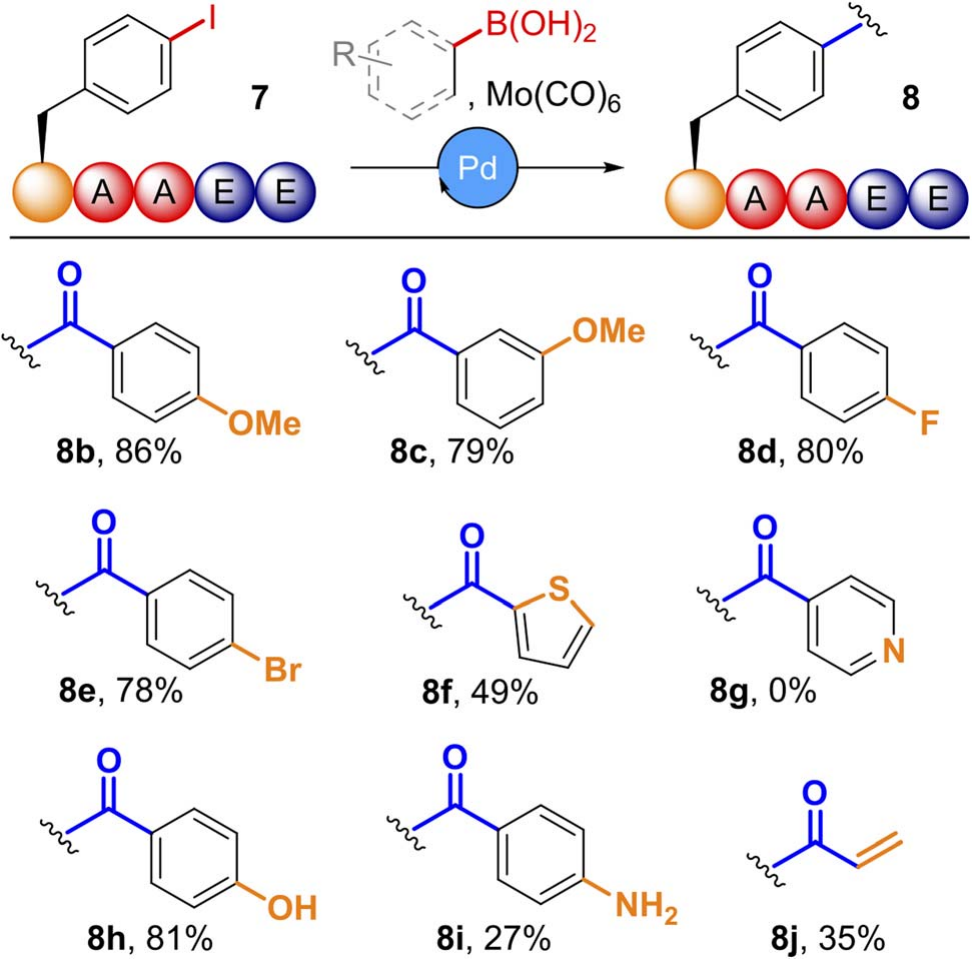
Conversion of *p*IPhe-AAEE, 7, to carbonylative products, 8, with varying boronic acids; Reaction conditions: Mo(CO)_6_ (2 equiv.), Pd(TFA)_2_ (0.4 equiv.), boronic acid (8 equiv.), acetonitrile : PBS (pH 8, 4 : 6), 37 °C, 20 h.

Having optimised conditions for cSMCC, we subsequently applied these to a diverse panel of coupling partners (Fig. 3 See SI Section 5.7 for further details). A screen of organoboronic acids revealed that both electron-rich and electron-deficient substrates were tolerated, with relative conversions ranging from 66–86% for *para-* and *meta*-substituted methoxy (8b,c) and fluoro (8d) phenyl boronic acids. Couplings with halogenated boronic acids (8e), heteroaromatics (8f), unprotected phenols (8h) and anilines (8i) were all tolerated, though 4-pyridinylboronic acid resulted in no conversion (8g). Potassium trifluoroborates were also viable coupling partners, giving comparable conversions to the parent boronic acid. Potassium vinyltrifluoroborate provided the related vinyl ketone 8j with 35% relative conversion, albeit with a high degree of competition from the corresponding vinylbenzene SMCC product (37% conversion). These results demonstrate the potential of our methodology to generate a wide-range of functionalised diaryl-ketones *via* late-stage modification of a common peptide precursor.

With these results in hand, we applied our optimised conditions to the preparative scale modification of peptides. Couplings were performed on a ∼20 µmol scale and after completion were centrifuged to remove insoluble molybdenum and palladium byproducts. The supernatants were then treated with a commercial dimercaptotriazine-scavenger resin to remove remaining palladium, before peptide purification *via* reverse-phase chromatography. On this scale, phenyl-diaryl ketone 8a was isolated in an 81% yield.

4-Methoxy and 4-fluoro phenylboronic acids were also coupled successfully on this scale, to generate 8b (34%) and 8d (32%) respectively. The reduction in isolated yield relative to 8a was a result of loss during purification, rather than reduced conversion, as indicated by LC-MS analysis of the crude reaction mixtures.

**Fig. 4:**
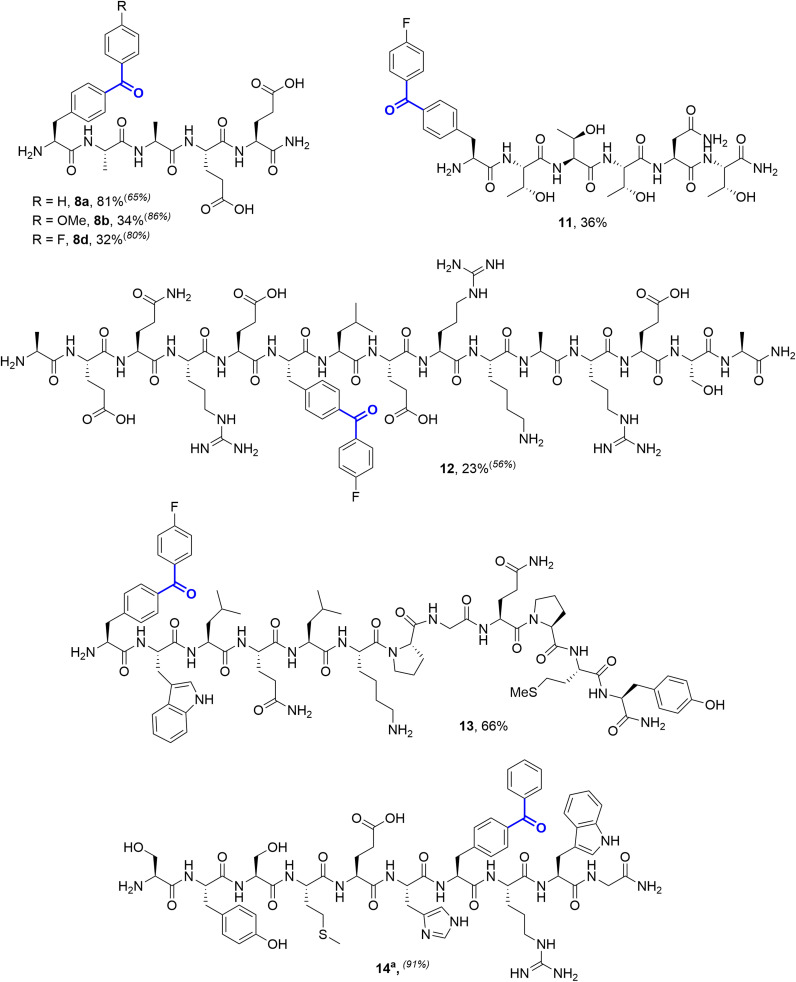
Isolated yields of cSMCC-modified peptides. Reactions performed on a 20 mmol scale with 40 mol% ‘Pd(TFA)_2_’, 2 equiv. Mo(CO)_6_, and 8 equiv. boronic acid in 6 : 4 potassium phosphate buffer (250 mM, pH 8):acetronitrile. Reactions were incubated at 37 °C for 20 h. Relative conversions for small scale reactions (1.5 µmol) are provided in superscript parentheses where relevant. ^a^Run with 100 mol% ‘Pd(TFA)_2_’.

Couplings were subsequently successfully performed on a number of peptide substrates, on both preparative and analytical scale, demonstrating the tolerance of the reaction to the wide-range of functional groups present on unprotected peptides. Peptides based on the HIV entry inhibitor Peptide T (11), an algal RuBisCO-binding domain (12), the yeast pheromone alpha-factor (13), and the N-terminal fragment of human adrenocorticotropic hormone (14) all successfully underwent cSMCC with 4-fluorophenyl boronic acid (Fig. 4). In the case of 14, low conversions were observed under our standard conditions, which we attributed to the histidine residue in the sequence. This could be easily addressed, either through the addition of extra palladium precatalyst, or through the addition of sodium ascorbate as a reductant.^29^

4-Fluorobenzophenones have been previously shown to exhibit greatly enhanced photo-affinity labelling efficiency relative to unsubstituted Bpa.^7^ Modification of these sequences demonstrated that coupling is effective at both internal positions and at peptide termini. In cases where yields were reduced, LC-MS of the crude reaction mixtures indicated high conversion to the desired cSMCC products, and losses occurred primarily during purification. For example, for Peptide T 78% relative conversion to 11 was observed *via* LC-MS, with an isolated yield of 36%. An exception to the functional group tolerance was the presence of unprotected thiols, with no conversion being observed for a free cysteine-containing decapeptide derivative of laminin (H_2_N-CDPGYIGSR-*p*IPhe-CONH_2_, 15, see SI Section 5.11).

**Fig. 5:**
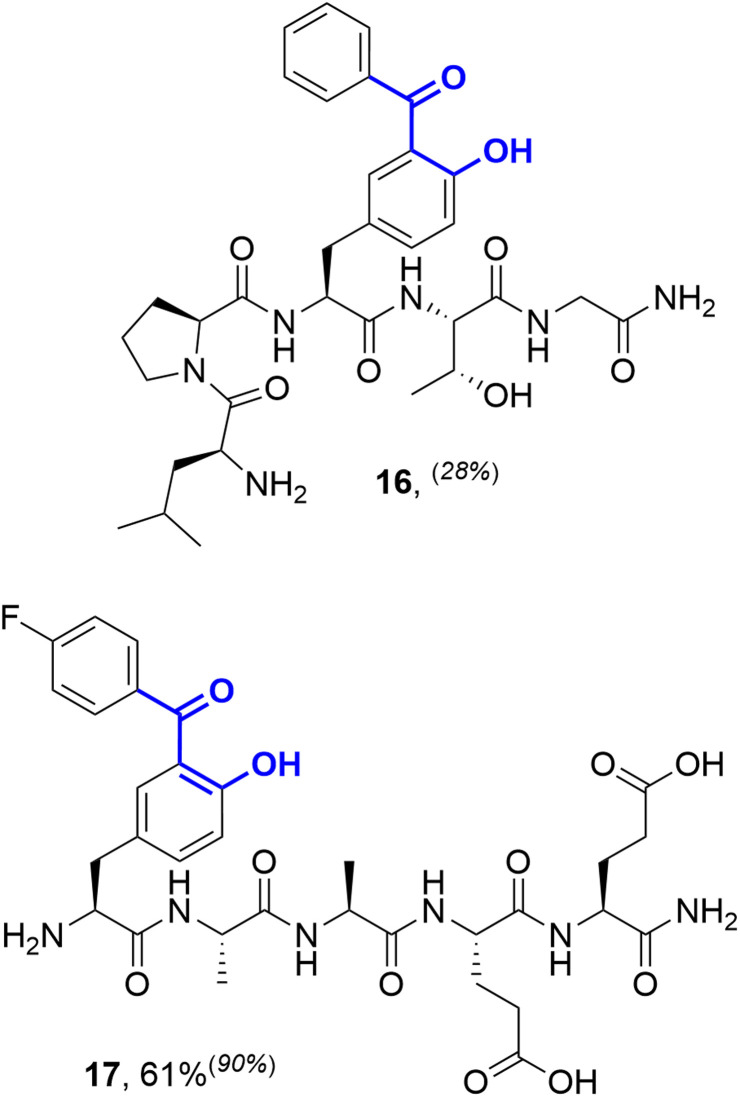
Synthesis of peptides modified *via* cSMCCs at 3-iodotyrosine residues. Reactions performed on a 20 mmol scale with 40 mol% ‘Pd(TFA)_2_’, 2 equiv. Mo(CO)_6_, and 8 equiv. boronic acid in 6 : 4 potassium phosphate buffer (250 mM, pH 8):acetronitrile. Reactions were incubated at 37 °C for 20 h. Relative conversions for small scale reactions (1.5 µmol) from LC-MS analysis are provided in superscript parentheses where relevant.

**Scheme 2:**
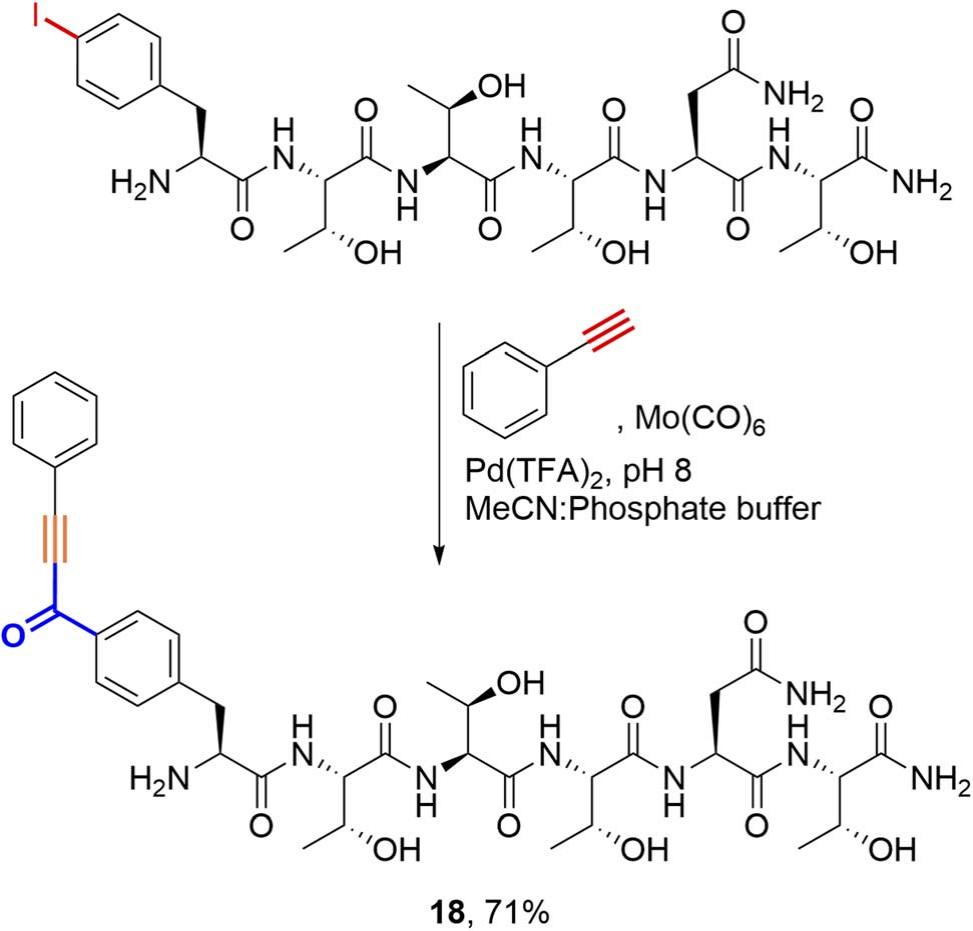
Carbonylative-Sonogashira cross-coupling applied to Peptide T on a 20 mmol scale. Reaction conditions: Mo(CO)_6_ (2 equiv.), Pd(TFA)_2_ (0.4 equiv.), phenylacetylene (8 equiv.), acetonitrile:PBS (pH 8, 4 : 6), 37 °C, 20 h.

Considering alternative halogenated amino acids, 3-iodotyrosine containing peptides based on the sortase-recognition sequence LPTYG and a model peptide H_2_N-3ITyr-AAEE-CONH_2_ also underwent successful coupling, to generate the corresponding 2-hydroxybenzophenones 16 and 17 (Fig. 5). The 3-iodotyrosine could be installed either directly during SPPS, or *via ortho*-iodination of tyrosine residues within cleaved peptides with Barluenga's reagent (IPy_2_BF_4_) prior to cross-coupling.^30^ In this scenario, careful purification of the iodinated peptide was essential to remove the pyridine side-product of iodination, which may otherwise inhibit cSMCC. Finally, we also demonstrated that carbonylative copper-free Sonogashira couplings (carbonylative Heck–Cassar–Sonogashira type-reactions) were also viable under our optimised conditions, with alkynyl-aryl ketone 18 being isolated in a 70% yield following the coupling of phenylacetylene to Peptide T (Scheme 2).

## Conclusions

In this study, we have demonstrated that carbonylative Suzuki–Miyaura cross-couplings offer a viable method for the late-stage diversification of peptide substrates, and access to peptide-based diaryl ketones for photo-affinity labelling. Key to this work is the use of Mo(CO)_6_ as a carbon monoxide surrogate, which negates the need for CO atmospheres which would otherwise make cSMCCs challenging on the small scales required for peptide modification, and without the specialist equipment needed to work under such atmospheres safely. As such, our operationally simple methodology, which can be performed in an open atmosphere, under mild aqueous conditions, is translatable to diverse laboratory settings.

Optimisation of the cSMCC reaction conditions and reagent loadings allowed us to demonstrate the tolerance of the reaction to a broad range of boronic acid coupling partners, as well as applicability to two common unnatural iodinated amino acids, *p*IPhe and 3-ITyr. Both of these amino acids can be integrated into proteins, in the case of 3-ITyr by both codon expansion technologies and chemical modification. Though the reliance on an acetonitrile co-solvent is currently limiting for the translation of this chemistry onto such proteins, we were able to show that cSMCC was possible at as high as 90% water, offering possibilities for further optimisation to open up this pathway. We are currently working to develop the chemistry further with this goal in mind, as well as exploring the use of alternative carbonylative routes to access diverse ‘on-peptide’ chemical space.

## Author contributions

James Kleppen and Neil Scott contributed equally to this manuscript. JPK, NWJS, TJP, and KML synthesised and modified the peptides described in this study. NWJS and KSS synthesised and modified amino acid models and substrates for SPPS. JRD undertook preliminary studies that led directly to this work. LJB supervised and supported student researchers involved in the project. IJSF and CDS developed the project. KSS and CDS wrote the manuscript. CDS supervised and managed the study. All authors contributed to the editing of the manuscript.

## Conflicts of interest

The authors declare no competing financial interests.

## Supplementary Material

SC-017-D5SC05588A-s001

## Data Availability

All datasets associated with this manuscript will be made freely accessible through the University of York's PURE research depository. Supplementary information: all experimental details, including peptide synthesis, reaction protocols, and full screening results. See DOI: https://doi.org/10.1039/d5sc05588a.
