# Supplementary material for: Peptide modification via a mild carbonylative Suzuki–Miyaura reaction gives late-stage access to diaryl-ketones
Source: Chem Sci. 2026 May 5;17(24):11858–64. doi: 10.1039/d5sc05588a (PMC13169266; doi:10.1039/d5sc05588a)
Supplement: SC-017-D5SC05588A-s001 [file SC-017-D5SC05588A-s001.pdf]

Supplementary Information (SI) for Chemical Science.
This journal is © The Royal Society of Chemistry 2026

Peptide modification via a mild carbonylative Suzuki-Miyaura reaction gives late-stage access to diaryl-ketones

James P. Kleppen,[a,b]* Neil W. J. Scott,[a,b]* Tom J. Philiips,[a,b] Ksenia S. Stankevich,[a,b] James R. Donald,[a,b] Lydia J. Barber,[a,b] Katie M. Lewis,[a,b] Ian J. S. Fairlamb,[a] and Christopher D. Spicer*[a,b]

[a]Department of Chemistry, University of York, Heslington, YO10 5DD, UK.

[b]York Biomedical Research Institute, University of York, Heslington, YO10 5DD, UK.

Table of contents

S2 General considerations

S3 Supplementary figures

S5 Peptide synthesis

S12 Amino acid synthesis

S15 cSMCC on single amino acids

S17 Optimisation of cSMCC conditions on peptides

S34 Peptide iodination with Barluenga's reagent

S34 Large scale peptide modifications

S44 Peptide MS/MS analysis

S47 References

S48 Peptide NMRs and HRMS

S61 Small molecule NMRs and HRMS

General considerations

Proton and carbon nuclear magnetic resonance (^{1}H and ^{13}C NMR respectively) spectra were recorded on a Jeol ECX-400 (400 MHz) or Bruker AVIIIHD (600 MHz) spectrometer. NMR shifts were assigned using COSY, HSQC and HMBC spectra. All chemical shifts are quoted on the δ scale in ppm using residual solvent as the internal standard (^{1}H NMR: $CDCl_3$ = 7.26; MeOD = 3.31; D_2O = 4.69; DMSO-d_6 = 2.50 and ^{13}C NMR: $CDCl_3$ = 77.16, MeOD = 49.00, DMSO-d_6 = 39.52). Coupling constants (*J*) are reported in Hz with the following splitting abbreviations: s = singlet, d = doublet, t = triplet, q = quartet, m = multiplet, app = apparent, br = broad. Melting points (m.p.) were recorded on a Gallenkamp melting point apparatus. Infrared (IR) spectra were recorded on a Perkin Elmer UATR Two FT-IR spectrometer or a Bruker Alpha II ATR spectrometer with Opus build 8.5.29. Absorption maxima (υ_{max}) are reported in wavenumbers (cm^{-1}). High resolution electrospray ionisation (ESI) mass spectra (HRMS) were recorded on a Bruker Compact TOF-MS or a Jeol AccuTOF GCx-plus spectrometer. Nominal and exact *m/z* values are reported in Daltons (Da).

Thin layer chromatography (TLC) was carried out using aluminium backed sheets coated with 60 F_{254} silica gel (Merck). Visualization of the silica plates was achieved using a UV lamp (λ_{max} = 254 nm), potassium permanganate (5% $KMnO_4$ in 1M NaOH with 5% potassium carbonate), or ninhydrin (1.5% ninhydrin, 3% AcOH in *n*-butanol). Flash column chromatography was carried out using Geduran Si 60 (40-63 µm) (Merck). Mobile phases are reported as % volume of more polar solvent in less polar solvent. Anhydrous solvents were dried over a PureSolv MD 7 Solvent Purification System. Deionized water was used for chemical reactions and for protein manipulations. All other solvents were used as supplied (Analytical or HPLC grade), without prior purification. Reagents were purchased from Sigma-Aldrich, VWR, or Fluorochem and used as supplied, unless otherwise indicated. 'Pd(OR)$_2$' precatalysts refer to the [$Pd_3(OR)_6$] complexes. 'Pd(OAc)$_2$' was not purified prior to use and therefore contained levels of nitrite-contamination.[1] Brine refers to a saturated solution of sodium chloride. Petrol refers to the fraction of petroleum ether boiling in the range 40-60 °C. Anhydrous magnesium sulfate ($MgSO_4$) was used as the drying agent after reaction workup unless otherwise stated.

Liquid chromatography-mass spectrometry (LC-MS) was performed on a HCTultra ETD II ion trap spectrometer, coupled to an Ultimate300 HPLC using an Accucore C18

column (150 × 2.1 mm, 2.6 µm particle size). Water (solvent A) and acetonitrile (solvent B), both containing 0.1% formic acid, were used as the mobile phase at a flow rate of 0.3 mL min^{-1}. LC traces were measured via UV absorption at 220, 270, and 280. The gradient was programmed as shown below:

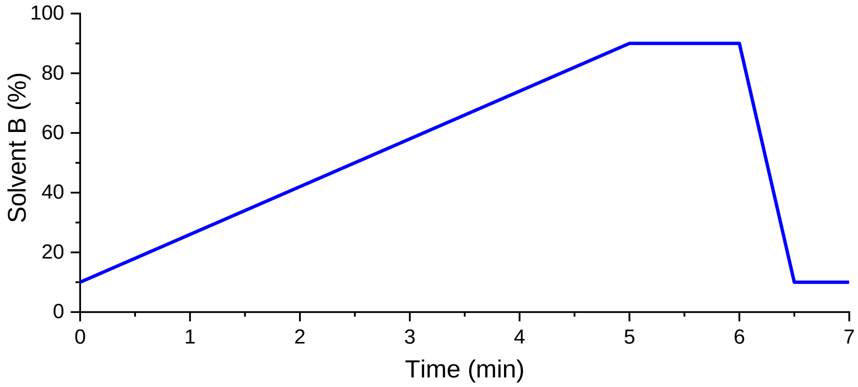

Spectra were analysed using the Bruker Data Analysis 4.4 software. Relative reaction conversions were determined from the integration of the mass spectrometry base peak chromatograms.

Supplementary figures

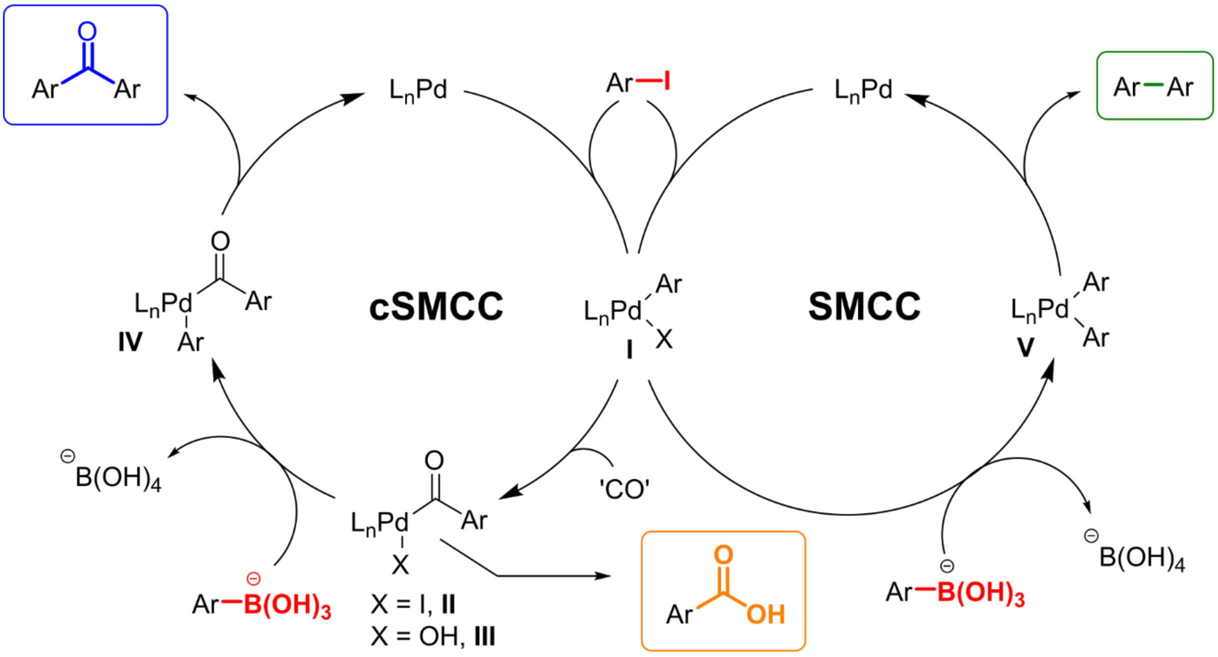

Figure S1: Catalytic cycle for the competition between carbonylative and non-carbonylative Suzuki-Miyaura cross-couplings, including elimination from the acylpalladium-hydroxo complex **III** to generate a carboxylic acid side-product.

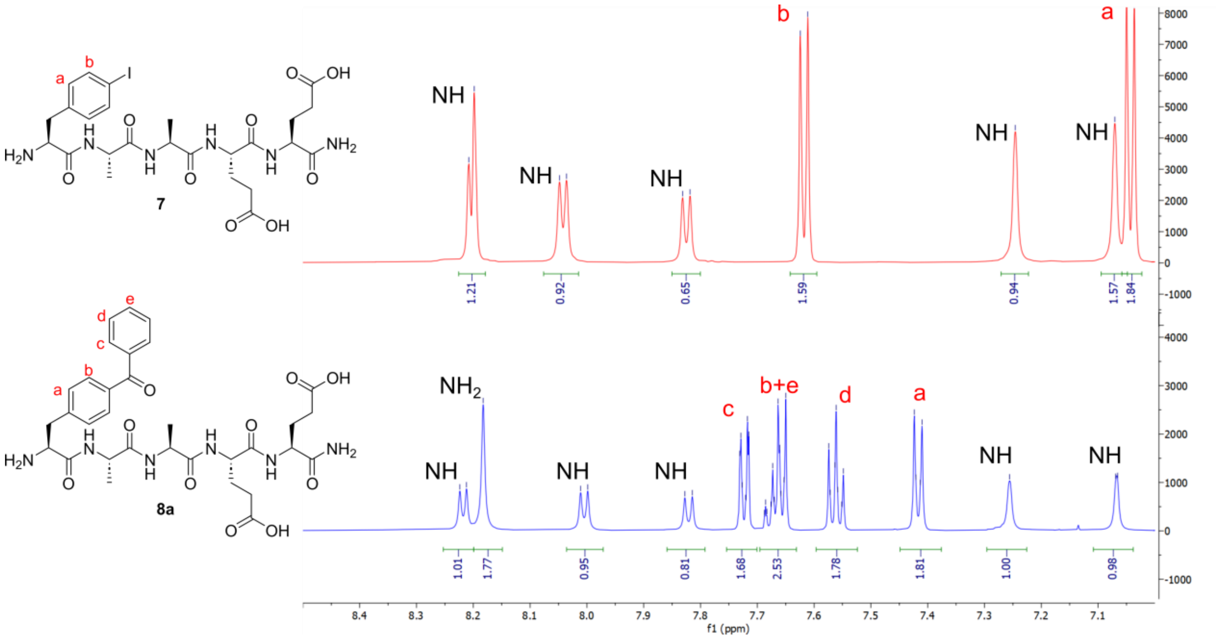

Figure S2: Aromatic region of the NMR spectra for peptides **7** and **8a**, demonstrating the formation of the benzophenone product. Spectra recorded in DMSO-d_6 at 600 MHz.

1. Peptide synthesis

Solid-phase peptide synthesis (SPPS) was performed on a CEM Liberty Lite or a CEM Liberty Blue 2.0 Automated Microwave Peptide Synthesiser, according to the manufacturer's standard protocols. Briefly, Fmoc-protected amino acids (5 equiv., 0.2 M in DMF) were coupled in the presence of *N,N'*-diisopropylcarbodiimide (DIC, 15 equiv.) and Oxyma Pure (5 equiv.), as coupling agent and base respectively, under microwave irradiation at a temperature of 90 °C for 2 minutes. Fmoc-*p*IPhe-OH and Fmoc-3-ITyr-OH were synthesised as described below and were installed into the peptides using the standard coupling cycles. Arginine was coupled at 25 °C, and cysteine and histidine were coupled at 50 °C for 10 minutes. Fmoc deprotection was performed using 10% piperidine in DMF at 90 °C for 60 seconds. Syntheses were performed on a 0.1 mmol scale, using Rink Amide MBHA resin (*C*-terminal amide, 0.5 mmol/g, 1% DVB, 100-200 mesh, NovaBiochem). Prior to cleavage, the resin was washed sequentially with DCM (3 × 15 mL) and methanol (3 × 15 mL). Peptides were cleaved from the resin in 20 mL of cleavage cocktail (90% TFA, 5% H_2O, 3% TIPS, 2% DTT for Cys-containing sequences) for 4 hrs (18 hrs for Arg-containing sequences). After filtration, the resin was washed extensively with DCM (3 × 50 mL) and the filtrate concentrated *in vacuo* to ~2 mL volume. The residue was dropped into ice cold diethyl ether (~ 50 mL), and the resultant precipitate collected by centrifugation (4000 rpm, 6 min), resuspended in diethyl ether, and centrifuged again. The residual solid was allowed to air dry for 10 min, then dissolved in deionized water (10 mL) and dried by lyophilisation.

Peptides were purified via reverse-phase flash column chromatography on a Teledyne CombiFlash NEXTGEN 300+ system, using a Redi*Sep* Rf Gold C18Aq column. The column was pre-equilibrated with water, and the peptide purified using a gradient from 5-100% MeCN:H_2O at a flow rate of 30 mL^{-1} min. The gradient programme was dependent on the peptide purified.

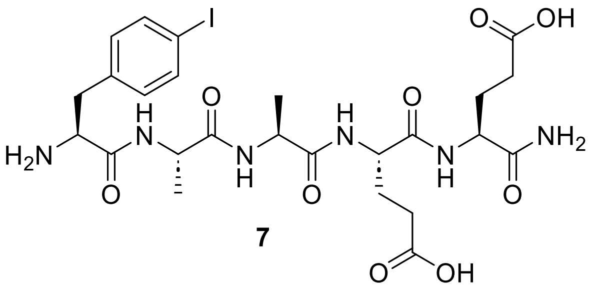

7

HRMS (ESI$^-$): *m/z*
Calcd for $C_{25}H_{34}IN_6O_9^-$: 689.1437 [M-H]$^-$;
Observed: 689.1403.

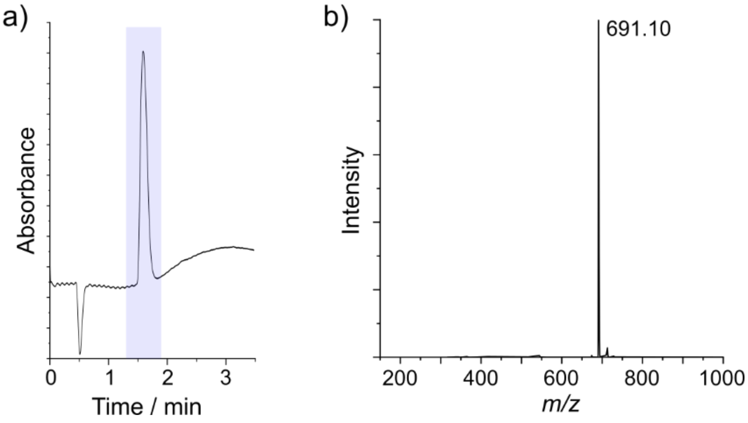

Figure S3: a) LC trace of **7**, with absorbance monitored at 280 nm; b) Low resolution mass spectrum ([M+H]$^+$) of **7**.

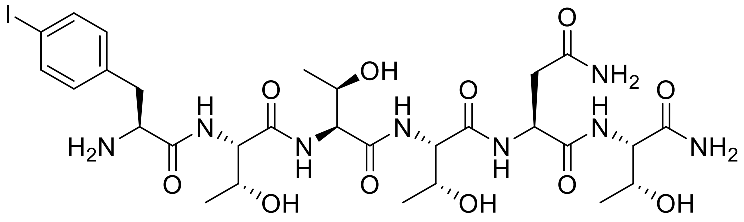

19

HRMS (ESI$^-$): *m/z*
Calcd for $C_{29}H_{44}IN_8O_{11}^-$: 807.2180 [M-H]$^-$;
Observed: 807.2157.

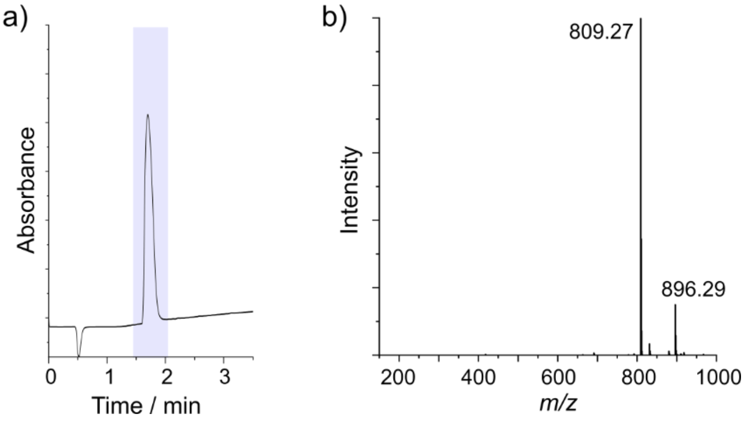

Figure S4: a) LC trace of **19**, with absorbance monitored at 280 nm; b) Low resolution mass spectrum ([M+H]$^+$) of **19**.

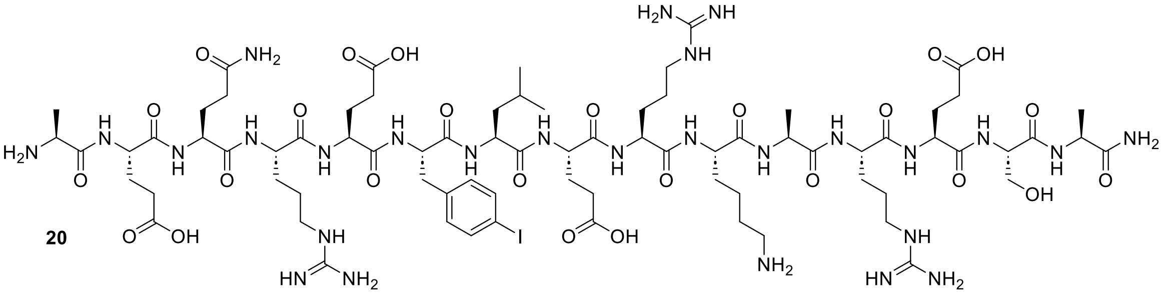

20

HRMS (MALDI+): *m/z* Calcd for $C_{76}H_{127}IN_{27}O_{25}^{+}$: 1944.8535 $[M+H]^{+}$; Observed: 1944.8566.

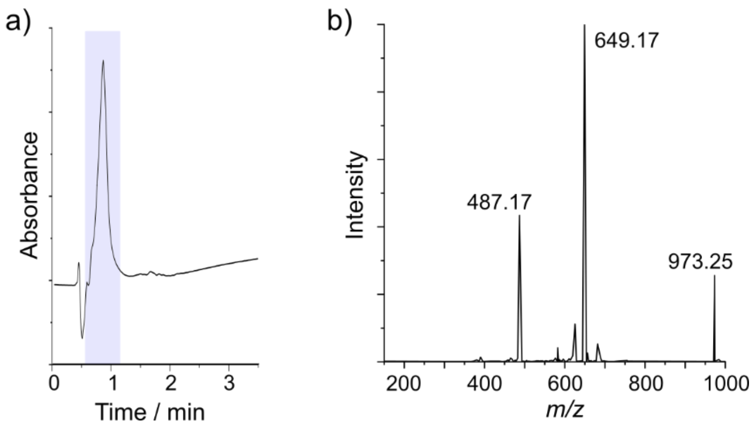

Figure S5: a) LC trace of **20**, with absorbance monitored at 280 nm; b) Low resolution mass spectrum($[M+2H]^{2+}$, $[M+3H]^{3+}$, $[M+4H]^{4+}$) of **20**.

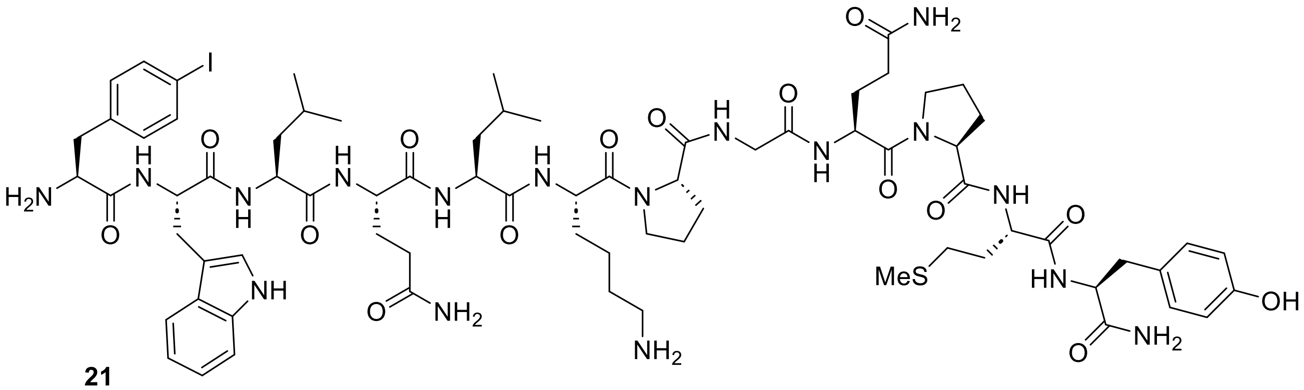

21

HRMS (ESI+): *m/z* Calcd for $C_{74}H_{106}IN_{17}O_{15}SNa_{2}^{2+}$: 838.8302 $[M+2Na]^{2+}$; Observed: 838.8319.

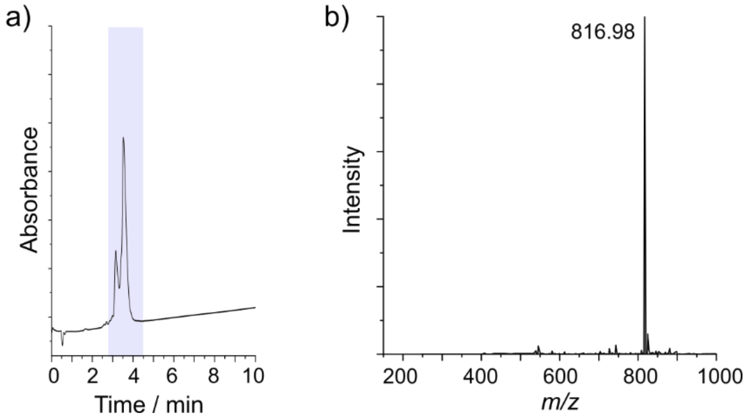

Figure S6: a) LC trace of **21**, with absorbance monitored at 280 nm; b) Low resolution mass spectrum ($[M+2H]^{2+}$) of **21**.

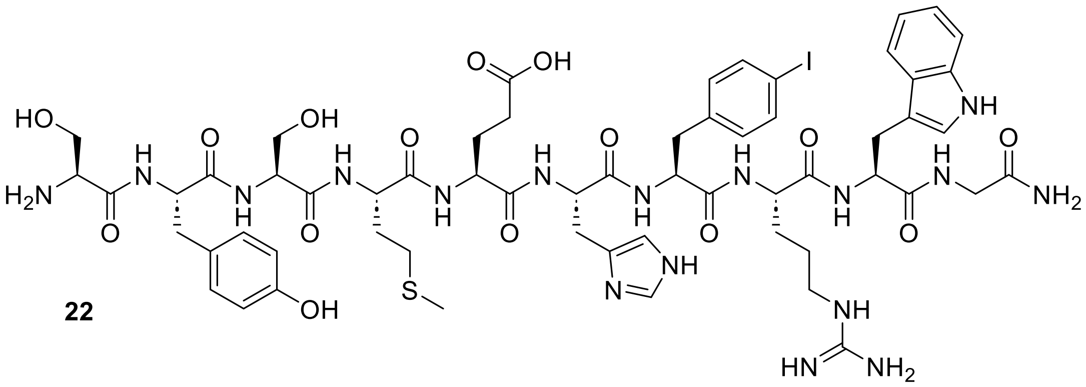

22

HRMS (ESI$^+$): *m/z* Calcd for $C_{59}H_{79}IN_{17}O_{15}S^+$: 1424.4701 $[M+H]^+$; Observed: 1424.4755.

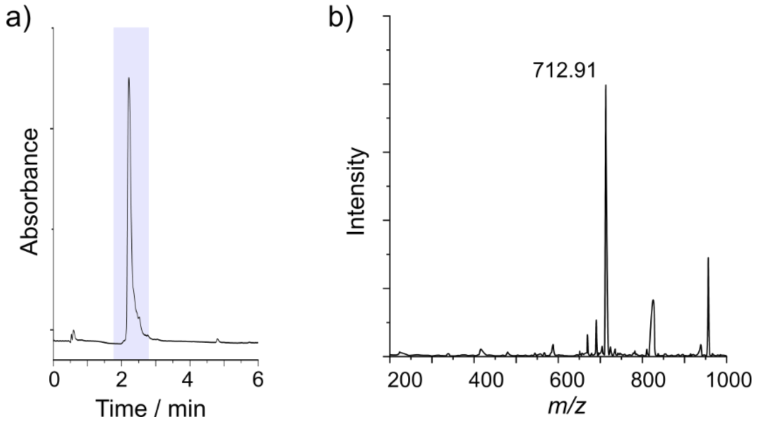

Figure S7: a) LC trace of **22**, with absorbance monitored at 280 nm; b) Low resolution mass spectrum ($[M+2H]^{2+}$) of **22**.

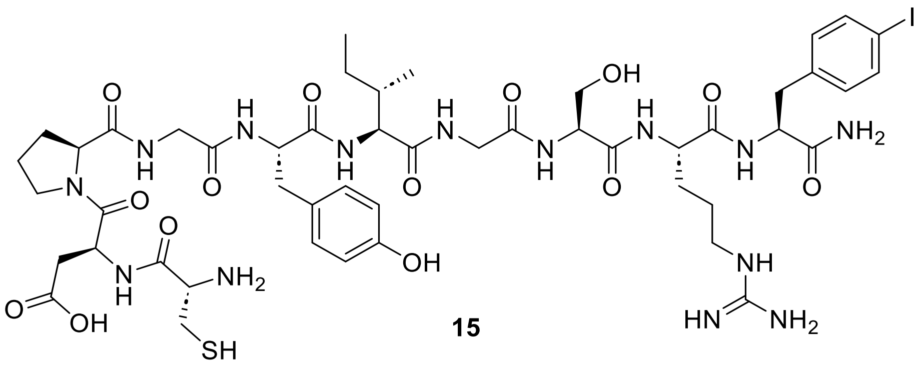

15

HRMS (ESI$^-$): *m/z* Calcd for $C_{49}H_{70}IN_{14}O_{14}S^-$: 1237.3967 [M-H]$^-$; Observed: 1237.3987.

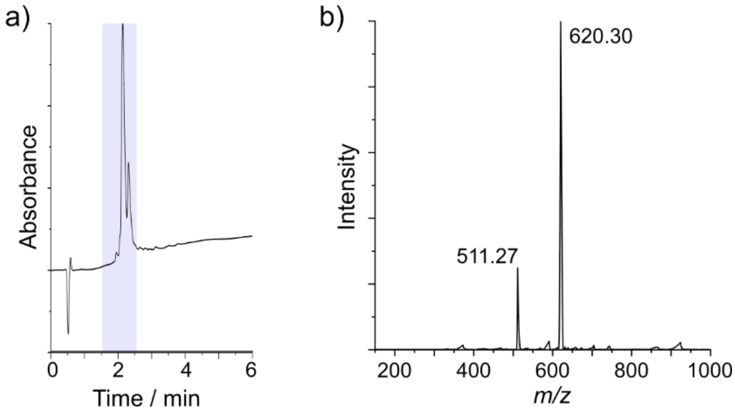

Figure S8: a) LC trace of **15**, with absorbance monitored at 280 nm; b) Low resolution mass spectrum ([M+2H]$^{2+}$) of **15**.

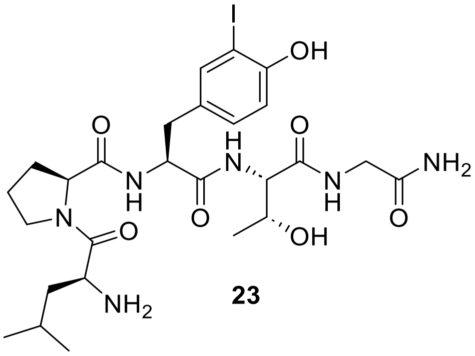

23

HRMS (ESI$^+$): *m/z* Calcd for $C_{26}H_{40}IN_6O_7^+$: 675.1998 [M+H]$^+$; Observed: 675.1998.

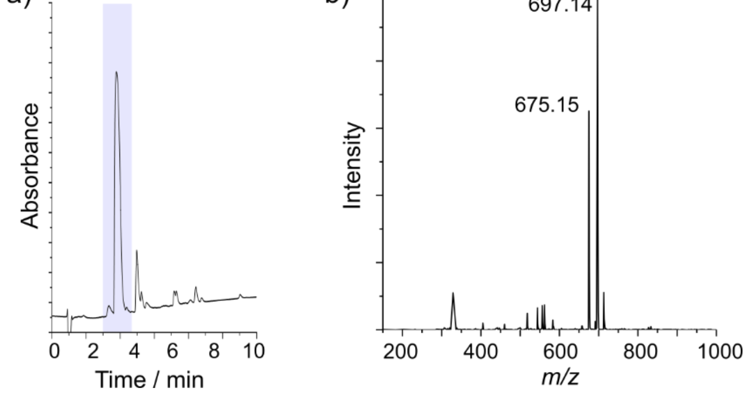

Figure S9: a) LC trace of **23**, with absorbance monitored at 280 nm; b) Low resolution mass spectrum ([M+H]$^+$, [M+Na]$^+$) of **23**.

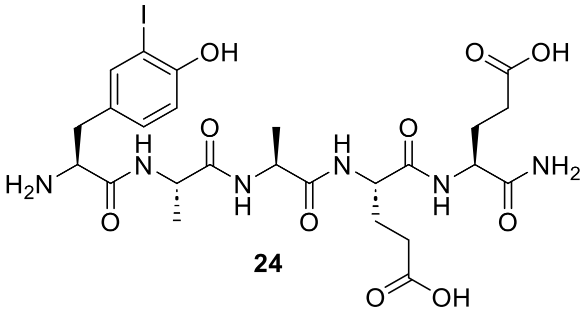

24

HRMS (ESI$^+$): *m/z* Calcd for $C_{25}H_{36}IN_6O_{10}$: 707.1532 [M+H]$^+$; Observed: 707.1543.

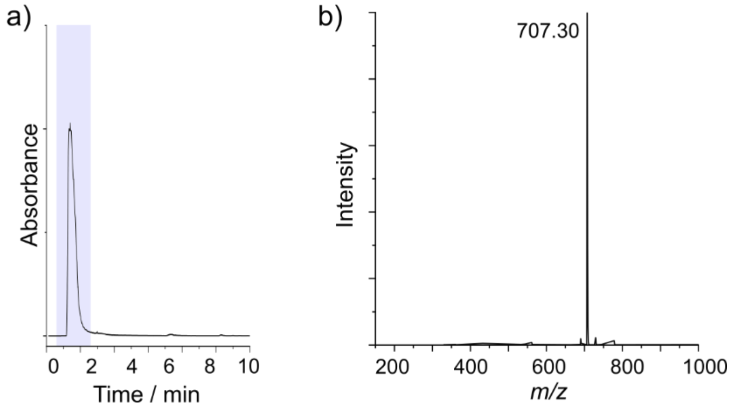

Figure S10: a) LC trace of **24**, with absorbance monitored at 280 nm; b) Low resolution mass spectrum ([M+H]$^+$) of **24**.

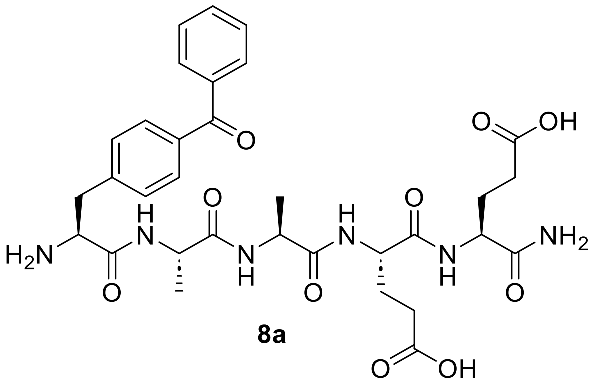

8a

HRMS (ESI$^+$): *m/z* Calcd for $C_{32}H_{41}N_6O_{10}^+$: 669.2912 [M+H]$^+$; Observed: 669.2879.

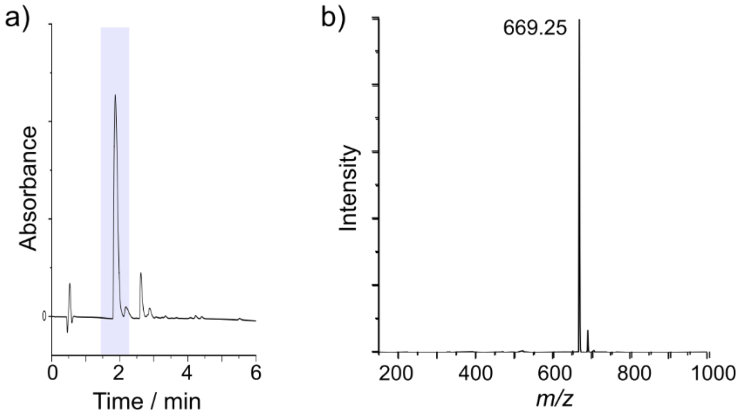

Figure S11: a) LC trace of **8a**, with absorbance monitored at 280 nm; b) Low resolution mass spectrum ($[M+H]^+$) of **8a**.

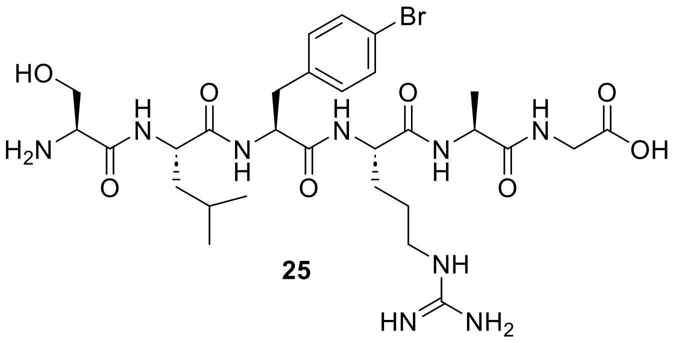

25

LRMS (ESI^+): *m/z* Calcd for $C_{29}H_{47}BrN_9O_8^+$: 728.26 $[M+H]^+$; Observed: 728.40.

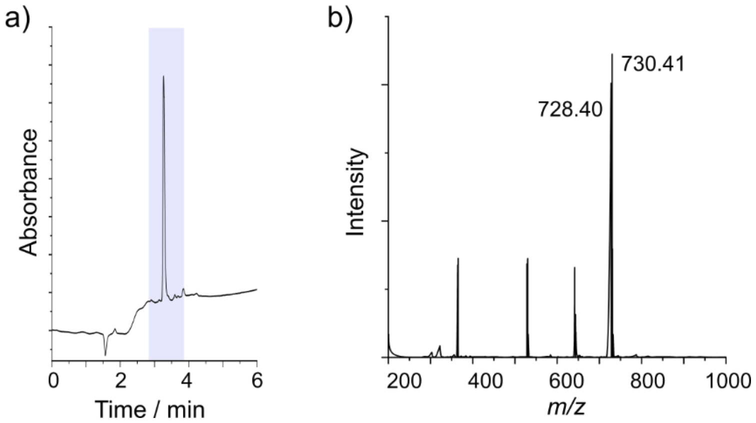

Figure S12: a) LC trace of **25**, with absorbance monitored at 280 nm; b) Low resolution mass spectrum ($[M+H]^+$) of **25**.

2. Amino acid synthesis

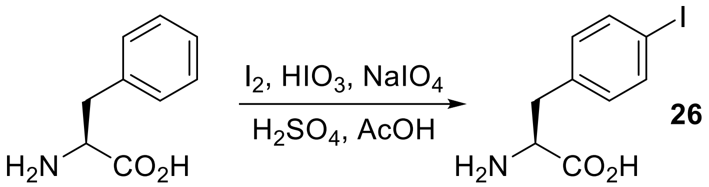

Iodine (25.2 g, 100 mmol) and iodic acid (8.8 g, 50 mmol) were added to a solution of L-phenylalanine (41.3 g, 250 mmol) in concentrated sulfuric acid (31 mL) and acetic acid (250 mL), and the mixture heated to 70 °C for 18 h. Sodium periodate (2 × 1.1 g, 10 mmol) was then added in two portions and heating at 70 °C continued for a further 30 min. The reaction was then cooled to r.t. and the acetic acid removed *in vacuo*. The crude residue was diluted with water (400 mL) and the aqueous washed with diethyl ether (2 × 200 mL) and DCM (2 × 300 mL). The aqueous was then basified with sodium hydroxide (2 M) until a white precipitate formed, which was collected by filtration and recrystalised from boiling water:ethanol (80 mL:80 mL). The resultant off-white crystals were collected by filtration and dried *in vacuo* to give the product (66.9 g, 230 mmol, 92%). Spectroscopic data were consistent with those previously reported.[2]

^{1}H NMR (400 MHz, NaOD/D_2O): δ = 7.61 (2H, d, *J* = 8.7 Hz, ArH2), 6.93 (2H, d, *J* = 8.7 Hz, ArH3), 3.35 (1H, t, *J* = 6.1 Hz, H_α), 2.82 (1H, dd, *J* = 14.5, 6.2 Hz, H_β), 2.65 (1H, dd, *J* = 14.5, 6.1 Hz, H_β).

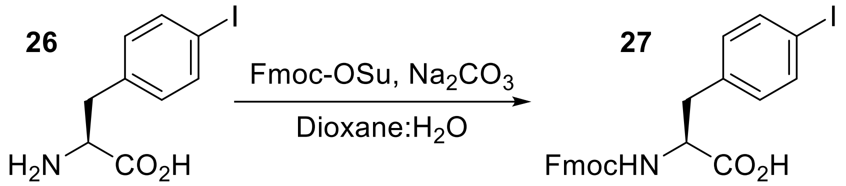

N-(9-Fluorenylmethoxycarbonyloxy)succinimide (3.65 g, 10.8 mmol) was added to a solution of L-*p*-iodophenylalanine **26** (3 g, 10.3 mmol) in dioxane (38 mL) and aqueous sodium carbonate (10% w/v, 30 mL), and the mixture stirred at r.t. for 18 h. After this time, the mixture was acidified carefully with hydrochloric acid (1 M, 100 mL) [CARE: GAS EVOLVED] and the aqueous extracted with ethyl acetate (3 × 100 mL). The combined organics were washed with brine (50 mL), dried with $MgSO_4$, filtered, and concentrated *in vacuo* to give the product as an off white solid (4.79 g, 9.3 mmol, 91%). Spectroscopic data were consistent with those previously reported.[3]

^{1}H NMR (400 MHz, MeOD): δ = 7.89 (2H, d, *J* = 7.6 Hz, Fmoc), 7.74 (2H, d, *J* = 8.3 Hz, ArH2), 7.56-7.68 (2H, m, Fmoc), 7.38-7.45 (2H, m, Fmoc), 7.25-7.34 (2H, m, Fmoc), 7.09 (2H, d, *J* = 8.3 Hz, ArH3), 4.09-4.24 (4H, m, Fmoc & H_{α}), 3.04 (1H, dd, *J* = 13.8, 4.4 Hz, H_{β}), 2.81 (1H, dd, *J* = 13.8, 10.8 Hz, H_{β}).

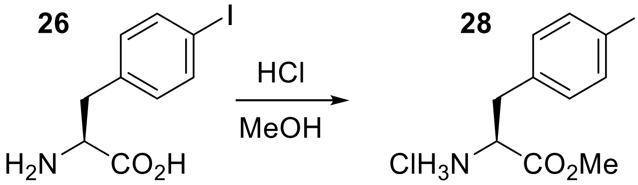

Acetyl chloride (6.1 mL, 86 mmol) was added dropwise to methanol (250 mL) at 0 °C, and stirred for 15 min. L-*p*-iodophenylalanine **26** (5.0 g, 17 mmol) was then added and the mixture stirred for 18 h. The mixture was then concentrated *in vacuo* to give the product as an off-white solid (5.85 g, 17 mmol, quant. yield). Spectroscopic data were consistent with those previously reported.[4]

^{1}H NMR (400 MHz, MeOD): δ = 7.73 (2H, d, *J* = 7.9 Hz, ArH2), 7.06 (2H, d, *J* = 7.9 Hz, ArH3), 4.32 (1H, dd, *J* = 7.2, 6.1 Hz, H_{α}), 3.81 (3H, s, -OMe), 3.22 (1H, dd, *J* = 14.5, 6.1 Hz, H_{β}), 3.13 (1H, dd, *J* = 14.5, 7.2 Hz, H_{β}).

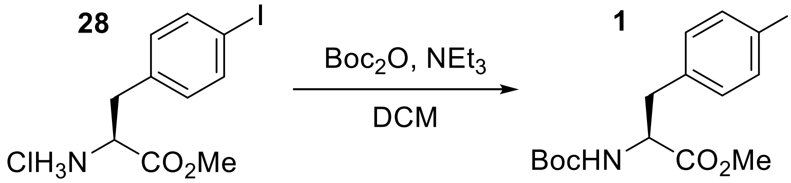

Di-*tert*-butyl decarbonate (3.6 g, 16.6 mmol) was added portion-wise to a solution of **28** (4.2 g, 13.8 mmol) and triethylamine (5.6 mL, 13.8 mmol) in DCM (100 mL) at 0 °C. The mixture was warmed to r.t. and stirred for 18 h. The mixture was washed with water (100 mL), and the organics were dried over $MgSO_4$, filtered, and concentrated *in vacuo*. The residue was purified by flash column chromatography, eluting with 20% EtOAc:Petrol. Pure fractions were concentrated *in vacuo* to provide the product as a white solid (1.95 g, 4.7 mmol, 34%). Spectroscopic data were consistent with those previously reported.[5]

^{1}H NMR (400 MHz, $CDCl_3$): δ = 7.60 (2H, d, *J* = 8.2 Hz, ArH2), 6.86 (2H, d, *J* = 8.2 Hz, ArH3), 4.96 (1H, br d, *J* = 8.5 Hz, -NH), 4.55 (1H, ddd, *J* = 8.5, 6.1, 5.8 Hz, H_{α}), 3.71 (3H, s, -OMe), 3.06 (1H, dd, *J* = 13.8, 5.8 Hz, H_{β}), 2.96 (1H, dd, *J* = 13.8, 6.1 Hz, H_{β}), 1.40 (9H, s, Boc).

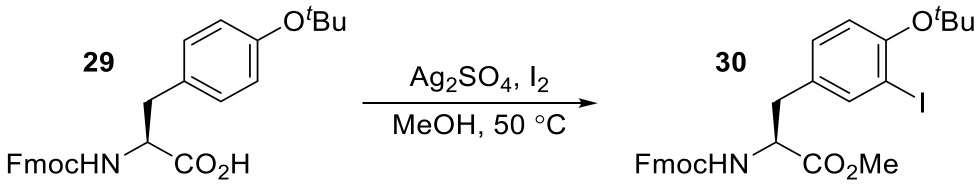

Ag_2SO_4 (0.81 g, 2.6 mmol, 1.3 equiv.) and I_2 (0.66 g, 2.6 mmol, 1.3 equiv.) were added to a round-bottom flask in the dark, followed by MeOH (38 mL, 0.05M), and the reaction was stirred at 50 °C for 30 min. Fmoc-L-Tyr(^{t}Bu)-OH **29** (0.92 g, 2.0 mmol, 1.0 equiv.) was then added in one portion and reaction was stirred at 50°C for a further 2.5 hours. Upon completion, the reaction was cooled to room temperature, filtered, and the solvent evaporated *in vacuo*. The residue was redissolved in ethyl acetate and the organic layer successively washed with $Na_2S_2O_3$ (0.1 M, 3 × 25 mL) and brine (3 × 25 mL), dried over $MgSO_4$, filtered and concentrated *in vacuo*. The crude product was purified by column chromatography using ethyl acetate/hexane gradient as an eluent (100/0 to 80/20). Fmoc-ITyr(^{t}Bu)-OMe **30** was isolated as a white crystalline solid (0.65 g, 1.1 mmol, 54%). Spectroscopic data were consistent with those previously reported.[6]

^{1}H NMR (400 MHz, DMSO-d_6): δ = 7.81-7.90 (3H, m, Fmoc & N<u>H</u>), 7.66 (1H, d, *J* = 2.1 Hz, Ar<u>H</u>2), 7.60 (2H, dd, *J* = 7.6, 3.7 Hz, Fmoc), 7.37 (2H, dd, *J* = 7.6, 1.4 Hz, Fmoc), 7.23-7.33 (2H, m, Fmoc), 7.14 (1H, dd, *J* = 8.4, 2.1 Hz, Ar<u>H</u>6), 6.97 (1H, d, *J* = 8.4 Hz, Ar<u>H</u>5), 4.07-4.24 (4H, m, Fmoc & <u>H</u>$_α$), 3.57 (3H, s, O<u>Me</u>), 2.94 (1H, dd, *J* = 13.8, 5.0 Hz, <u>H</u>$_β$), 2.77 (1H, dd, *J* = 13.8, 10.3 Hz, <u>H</u>$_β$), 1.29 (9H, s, O^{t}Bu).

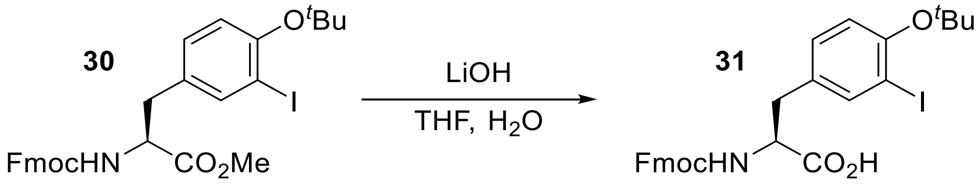

An aqueous solution of lithium hydroxide (0.1 M, 3.5 mL, 0.35 mmol) was added to a solution of **30** (105 mg, 0.17 mmol) in THF (5 mL) at 0 °C. The reaction was stirred for 30 min, and then another portion of lithium hydroxide solution (3.5 mL) was added. After stirring for 30 min, the reaction was acidified with HCl (0.1 M, 20 mL) and the aqueous extracted with ethyl acetate (3 × 20 mL). The combined organics were dried over $MgSO_4$, filtered and concentrated *in vacuo*. Fmoc-ITyr(^{t}Bu)-OH **31** was isolated as a white crystalline solid (88 g, 0.15 mmol, 86%). Spectroscopic data were consistent with those previously reported.[6]

^{1}H NMR (400 MHz, DMSO-d_6): δ = 7.76-7.89 (3H, m, Fmoc & NH), 7.68 (1H, d, *J* = 2.2 Hz, ArH2), 7.52-7.67 (2H, m, Fmoc), 7.37 (2H, dd, *J* = 7.6, 1.4 Hz, Fmoc), 7.25-7.30 (2H, m, Fmoc), 7.16 (1H, dd, *J* = 8.5, 1.4 Hz, ArH6), 6.97 (1H, d, *J* = 8.5 Hz, ArH5), 4.07-4.22 (4H, m, Fmoc & $H_α$), 2.97 (1H, dd, *J* = 13.8, 4.3 Hz, $H_β$), 2.73 (1H, dd, *J* = 13.8, 10.6 Hz, $H_β$), 1.30 (9H, s, O*t*Bu).

3. cSMCC on single amino acids

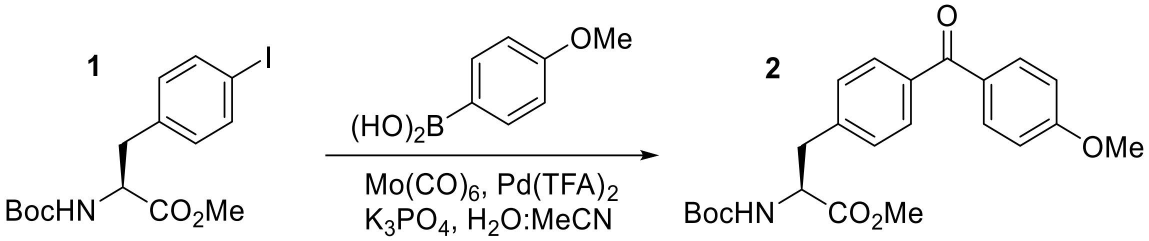

To a mixture of 'Pd(TFA)$_2$' (3.3 mg, 0.01 mmol, 2 mol%), Mo(CO)$_6$ (66 mg, 0.25 mmol), K_3PO_4 (318 mg, 1.5 mmol), **1** (203 mg, 0.50 mmol) and 4-methoxyphenyl boronic acid (113 mg, 0.75 mmol) was successively added water (0.5 mL) and acetonitrile (0.5 mL). The mixture was stirred for 24 h, and then diluted with brine (15 mL). The aqueous was extracted with ethyl acetate (3 × 20 mL) and the combined organics dried with Na_2SO_4, filtered, and concentrated *in vacuo*. The residue was purified by flash column chromatography, eluting with 15% EtOAc:Hexane. Pure fractions were concentrated *in vacuo* to give the product as a brown oil (175 mg, 0.42 mmol, 85%).

R_f: 0.26 (15% EtOAc:Petrol, UV active); **^{1}H NMR** (400 MHz, $CDCl_3$): δ = 7.74-7.85 (2H, m, anisyl-ArH3), 7.68 (2H, d, *J* = 8.2 Hz, phenyl-ArH2), 7.22 (2H, d, *J* = 8.2 Hz, phenyl-ArH3), 6.90-6.96 (2H, m, anisyl-ArH2), 5.03 (1H, br d, *J* = 8.3 Hz, -NH), 4.62 (1H, ddd, *J* = 8.3, 6.3, 5.7 Hz, $H_α$), 3.88 (3H, s, Ph-OMe), 3.73 (3H, s, -CO_2Me), 3.21 (1H, dd, *J* = 13.7, 5.7 Hz, $H_β$), 3.09 (1H, dd, *J* = 13.7, 6.3 Hz, $H_β$), 1.41 (9H, s, Boc); **^{13}C NMR** (101 MHz, $CDCl_3$): δ = 195.30 ($C=O_{ketone}$), 172.17 (-CO_2Me), 163.31 (anisyl-ArC1), 155.14 (-$CO_2{}^t$Bu), 140.62 (phenyl-ArC1), 137.09 (phenyl-ArC4), 132.61 (anisyl-ArC3), 130.24 (anisyl-ArC4), 130.17 (phenyl-ArC2), 129.32 (phenyl-ArC3), 113.66 (anisyl-ArC2), 80.21 (-CMe_3), 55.61 (-PhOMe), 54.34 ($C_α$), 52.50 (-CO_2Me), 38.45 ($C_β$), 28.38 (-CMe_3); **HRMS**: *m/z* (ESI$^+$) calc. for $C_{23}H_{27}NO_6$ $[M+Na]^+$ = 436.1736, obs. = 436.1731; **IR** ($υ_{max}$/ cm^{-1}): 3432 (N–H *amide*), 3075 (C–H), 3041 (C–H), 2990 (br, C–H), 1744

($\upsilon_{C=O}$ stretch, methyl ester), 1713 ($\upsilon_{C=O}$ stretch, carbamate), 1652 ($\upsilon_{C=O}$ stretch, ketone), 1603, 1368, 1305, 1172.

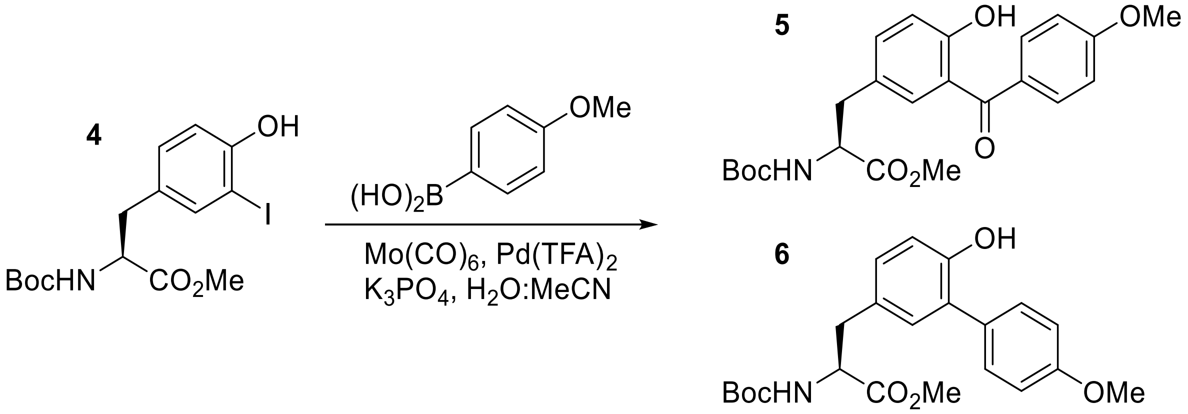

N-Boc-3-iodo-L-tyrosine methyl ester **4** (100 mg, 0.24 mmol), 4-methoxyphenylboronic acid (55 mg, 0.36 mmol), '$Pd(TFA)_2$' (4 mg, 0.012 mmol), $Mo(CO)_6$ (32 mg, 0.12 mmol) and K_3PO_4 (151.2 mg, 0.71 mmol, 3.0 Equiv.) were added to an oven-dried round bottom flask under air. Water (0.4 mL) and acetonitrile (1.6 mL) were then added successively, and the reaction mixture was stirred for 20 h. The mixture was then diluted with brine (20 mL) and extracted with EtOAc (3 × 20 mL). The combined organic layers were dried over $MgSO_4$, filtered, and concentrated *in vacuo*. The residue was purified by flash column chromatography, eluting with 0-25% EtOAc:Petrol. Fractions containing an inseparable mixture of **5** and **6** were concentrated *in vacuo* (76 mg, (**5**):(**6**) = 2:1, 51% yield **5**, 26% yield **6**).

Carbonylative Suzuki-Miyaura product (**5**)

^{1}H NMR (400 MHz, $CDCl_3$): δ 11.84 (1H, s, -OH), 7.71 (2H, ddd, *J* = 8.7, 2.4, 2.4 Hz, ArOMe-H2), 7.37 (2H, ddd *J* = 8.7, 2.4, 2.4 Hz, ArOMe-H3), 7.26 (1H, dd, *J* = 8.0, 4.0 Hz, ArOH-H6), 6.98-6.93 (1H, m, ArOH-H2), 6.87 (1H, d, *J* = 8.0 Hz, ArOH-H5), 4.99 (1H, br d, *J* = 8.5 Hz, -NH), 4.54 (1H, ddd, *J* = 8.5, 6.6, 6.4 Hz, H_α), 3.90 (3H, s, -OMe), 3.66 (3H, s, -OMe), 3.00 (1H, dd, *J* = 14.0, 6.4 Hz, H_β), 2.93 (1H, dd, *J* = 14.0, 6.6 Hz, H_β), 1.36 (9H, s, Boc); **^{13}C NMR** (100 MHz, $CDCl_3$): δ 199.9, 172.3, 163.1, 159.4, 155.2, 137.0, 133.9, 129.6, 129.3, 128.0, 119.3, 118.6, 113.9, 80.2, 55.6, 54.4, 52.4, 37.6, 28.3; **HRMS**: *m/z* (ESI^+): Calcd. for $C_{23}H_{27}NNaO_7$ $[M + Na]^+$ = 452.1680; Obs. = 452.1683.

Suzuki-Miyaura product (**6**)

^{1}H NMR (400 MHz, $CDCl_3$): δ 7.37 (2H, ddd, *J* = 8.5, 2.4, 2.4 Hz, ArOMe-H2), 7.00 (2H, ddd, *J* = 8.5, 2.4 Hz, 2.4 Hz, ArOMe-H3), 6.98-6.93 (2H, m, ArOH-H2 & H6), 6.87 (1H, d, *J* = 8.0 Hz, ArOH-H5), 5.39 (1H, br s, -OH), 4.99 (1H, br d, *J* = 8.5 Hz, -NH), 4.56 (1H, ddd, *J* = 8.5, 6.5, 5.9 Hz $H_α$), 3.85 (3H, s, -OMe), 3.71 (3H, s, -OMe), 3.08 (1H, dd, *J* = 13.9, 6.5 Hz $H_β$), 3.01 (1H, dd, *J* = 13.9, 5.9 Hz, $H_β$), 1.41 (9H, s, Boc); **^{13}C NMR** (101 MHz, $CDCl_3$): δ 172.6, 162.0, 155.3, 151.8, 132.0, 131.3, 130.3, 129.3, 128.1, 126.0, 116.0, 114.7, 80.1, 55.5, 54.6, 52.4, 37.6, 28.4; **HRMS:** *m/z* (ESI^+) calcd. for $C_{22}H_{27}NNaO_6$ $[M + Na]^+$ = 424.1731; obs. = 424.1735.

5. Optimisation of cSMCC conditions on peptides

General procedure for small scale cSMCC modification of peptides: A microcentrifuge tube was charged with molybdenum hexacarbonyl (0.8 mg, 3.0 µmol, 2 equiv.). Solutions of H_2N-*p*IPhe-EEAA-$CONH_2$ **7** (1.0 mg, 1.5 µmol) in PBS (450 µL, pH 8.0), phenylboronic acid (1.4 mg, 12 µmol, 8 equiv.) in acetonitrile (600 µL), and 'Pd$(TFA)_2$' (0.2 mg, 0.6 µmol, 0.4 equiv.) in PBS (450 µL, pH 8.0) were then sequentially added, and the tube was sealed and shaken at 37 °C for 20 h. A portion of the reaction mixture (200 µL) was then diluted with methanol (200 µL) and water (200 µL), sonicated for 30 s, then filtered through a 0.45 µM filter. The filtrate was then analysed by LC-MS. Conversions were approximated by integrating the areas of relevant base peak chromatogram peaks in the LC. Though this is a qualitative measurement due to potential differences in the ionisability of different species, these differences are likely to be small since the peptide backbone remains consistent, allowing comparative measurements.

5.1 Variation of palladium source

Run as described in the general procedure with either 7 equiv. of 'Pd$(TFA)_2$', Pd$(OPiv)_2$, 'Pd$(OAc)_2$', or 'Pd-ADHP' complex Pd$(\mathbf{L1})_1$ prepared as described,[2] in the presence of 24 equiv. $Mo(CO)_6$. Under these conditions the degree of SMCC product was higher, allowing the variations between experiments to be more easily observed.

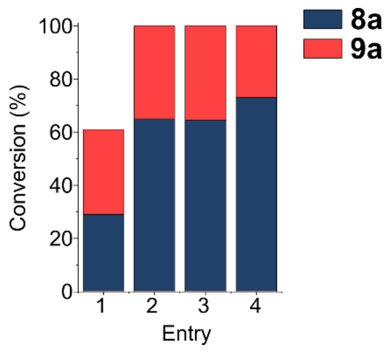

Entry	Pd(L)$_2$	8a (%)	9a (%)
1	Pd(OPiv)$_2$	29	32
2	Pd(OAc)$_2$	65	35
3	Pd(TFA)$_2$	64	36
4	Pd(**L1**)$_2$	73	28

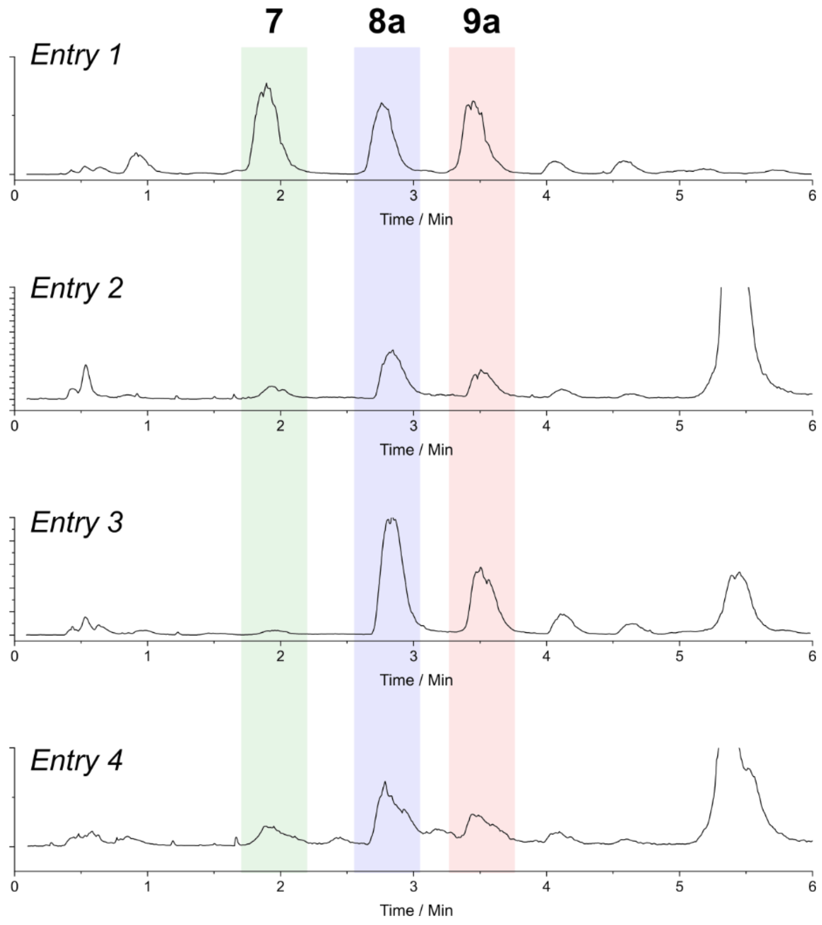

Figure S13: Base peak intensity LC traces with varying palladium source.

5.2 Variation of carbonyl source ratio

Run as described in the general procedure with 24 equiv. of either Mn(CO)$_6$, Mn$_2$(CO)$_{10}$, Fe$_2$(CO)$_9$, or Cr(CO)$_6$ in the presence of 7 equiv. of 'Pd(TFA)$_2$'.

Entry	CO source	8a (%)	9a (%)
1	-	0	92
2	Mo(CO)$_6$	72	26
3	Mn$_2$(CO)$_{10}$	28	57
4	Fe$_2$(CO)$_9$	39	56
5	Cr(CO)$_6$	0	57

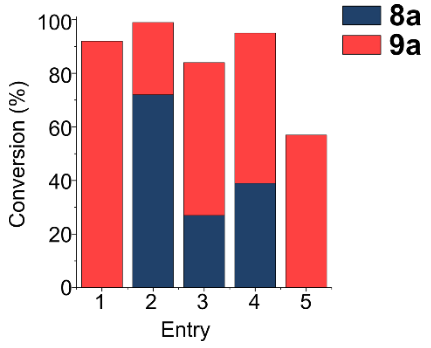

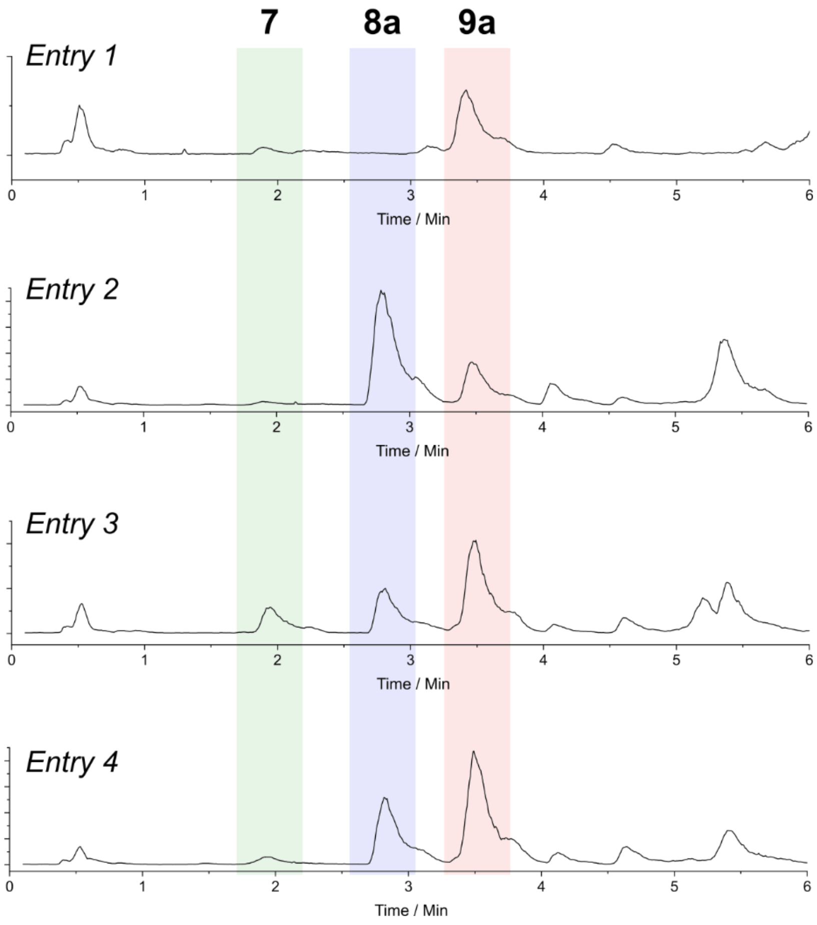

Figure S14: Base peak intensity LC traces with varying carbonyl source.

5.3 Variation of $Mo(CO)_6$ loading

Run as described in the general procedure altering the loadings of $Mo(CO)_6$ as shown in the table below.

Entry	$Mo(CO)_6$ equiv	8a (%)	9a (%)
1	24	81	1
2	12	68	1
3	6	67	1
4	3	64	1
5	2	65	1
6	1	64	3
7	0.5	64	1
8	0.1	24	1
9	0	0	11

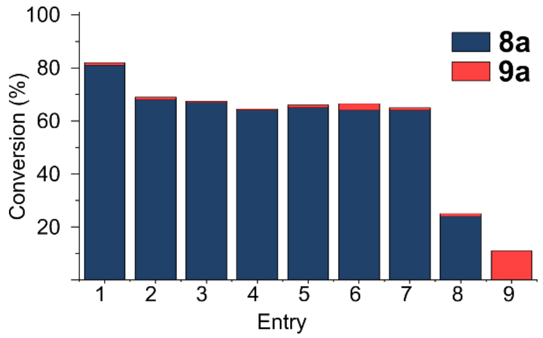

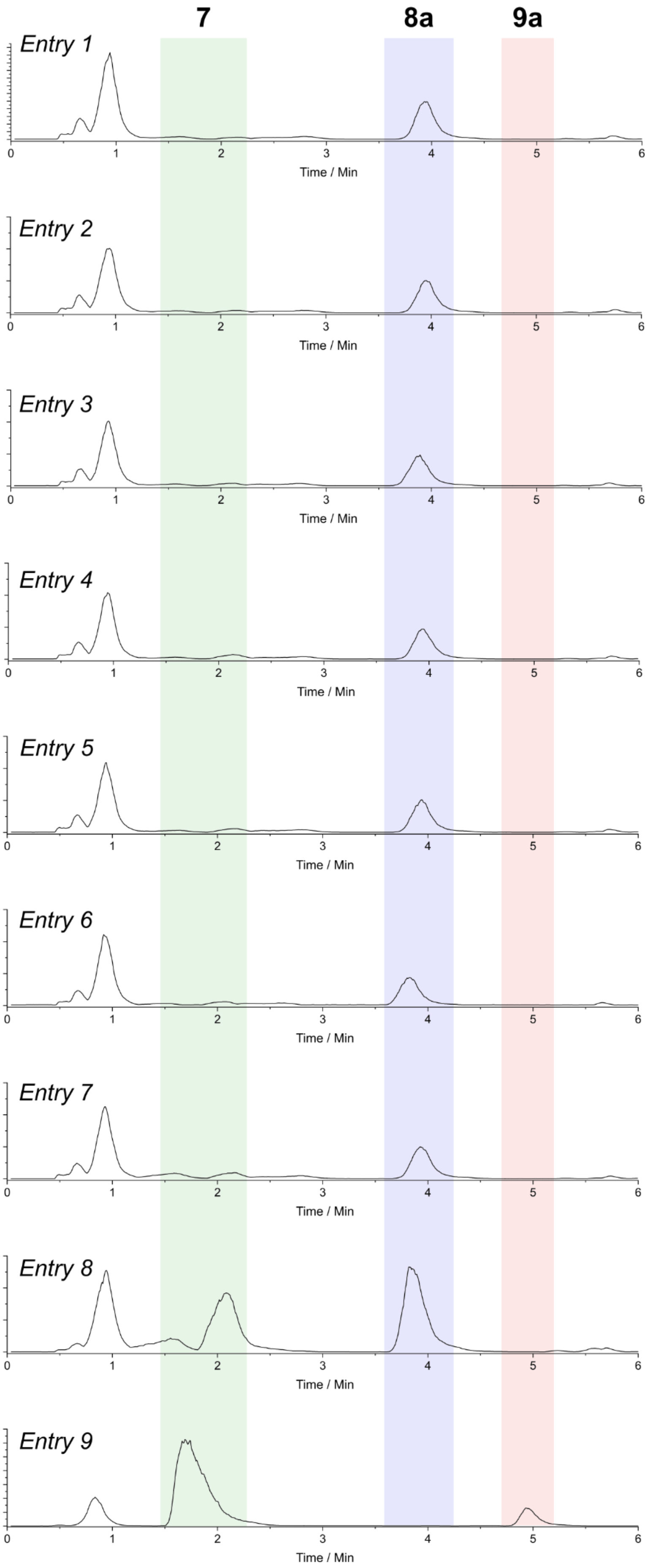

Figure S15: Base peak intensity LC traces with varying $Mo(CO)_6$

5.4 Variation of Pd(TFA)$_2$ loading

Run as described in the general procedure altering the loadings of 'Pd(TFA)$_2$' as shown in the table below.

Entry	Pd(TFA)$_2$ equiv	8a (%)	9a (%)
1	0.8	68	2
2	0.4	67	1
3	0.2	60	1
4	0.1	53	1
5	0	0	0

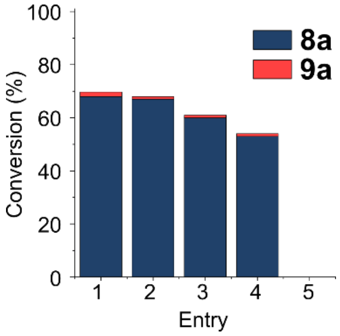

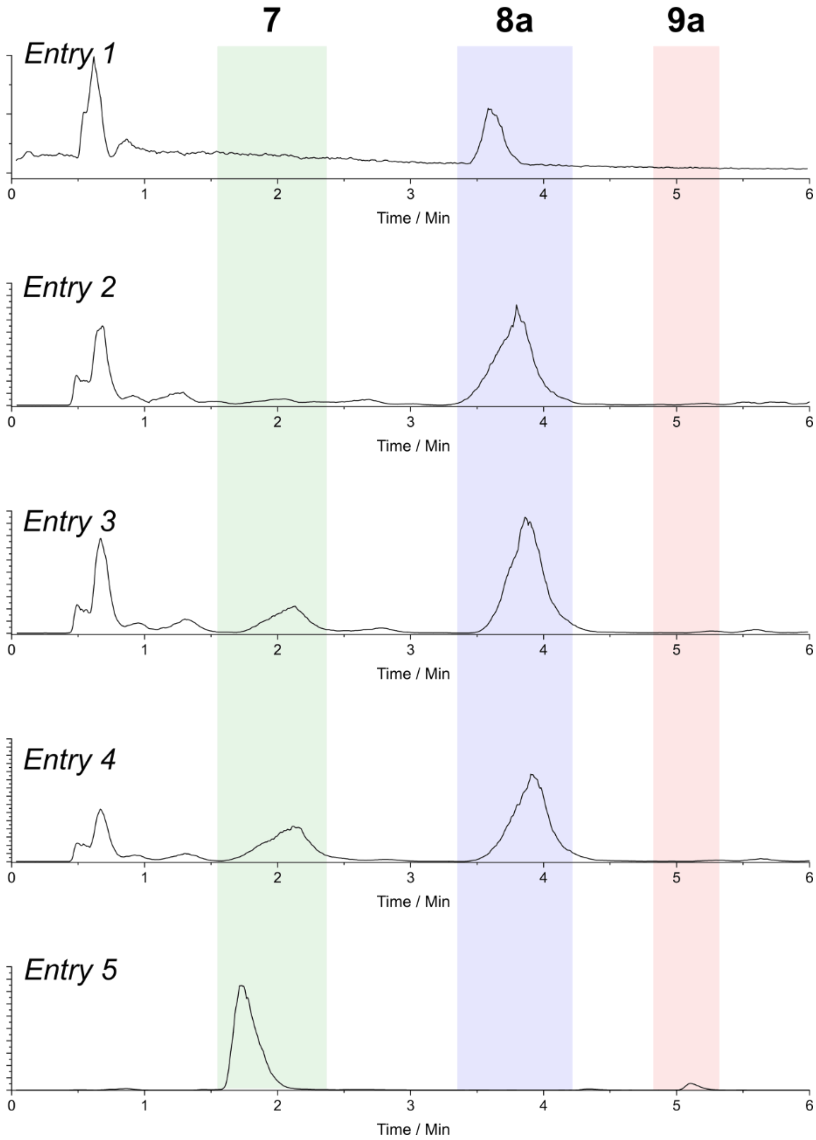

Figure S16: Base peak intensity LC traces with varying Pd(TFA)$_2$ loading.

5.5 Variation of boronic acid loading

Run as described in the general procedure altering the loadings of phenylboronic acid as shown in the table below.

Entry	Boronic acid equiv	8a (%)	9a (%)	10a (%)
1	20	80	1	14
2	16	79	1	15
3	12	73	1	20
4	8	67	1	25
5	4	48	1	41
6	2	27	1	45
7	0	0	0	71

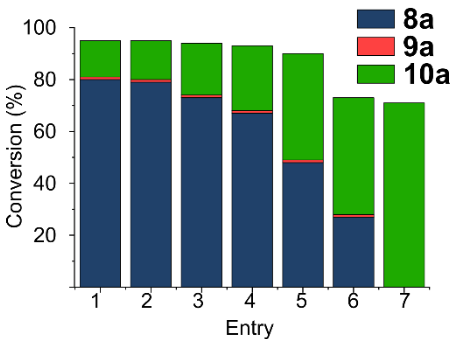

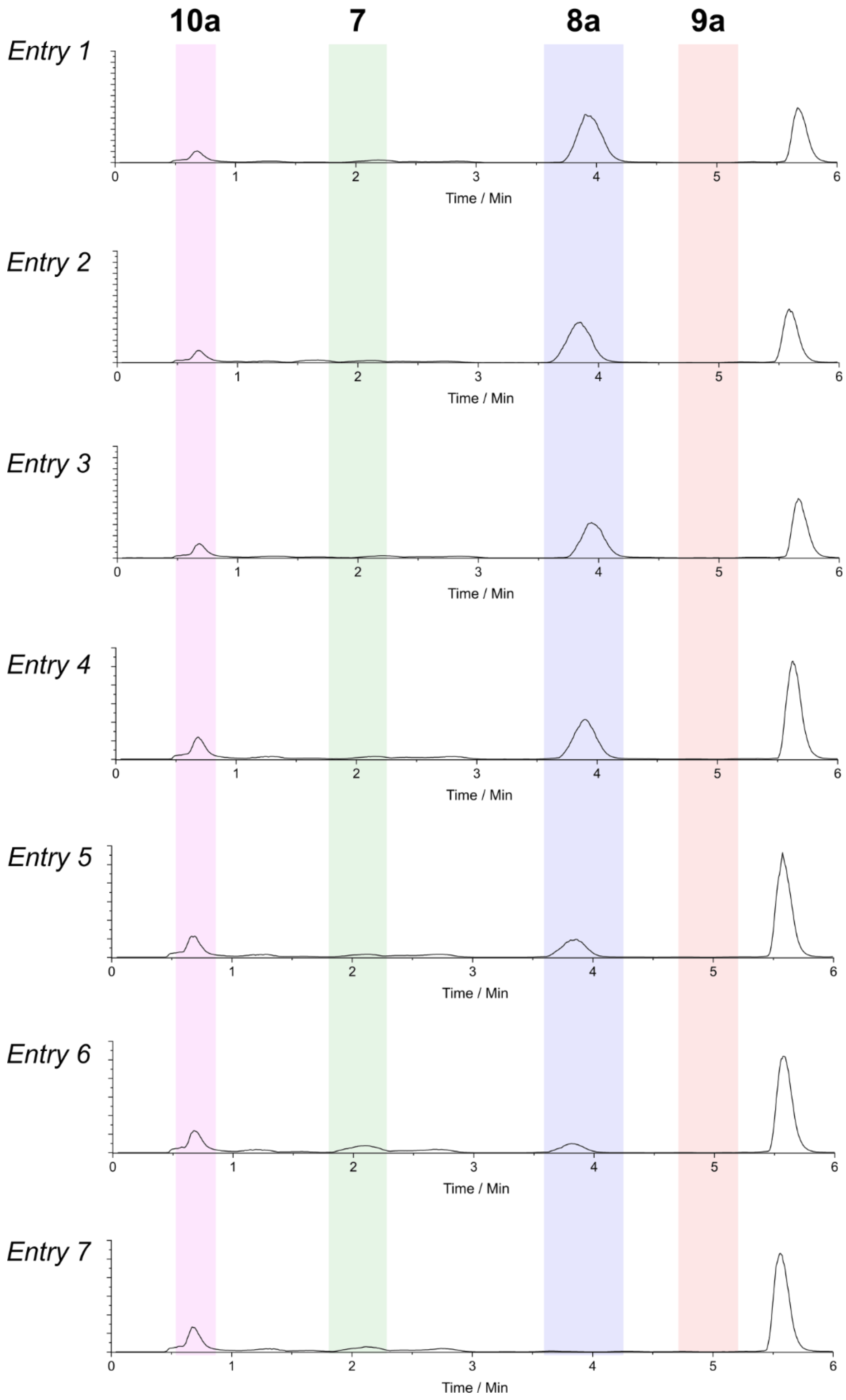

Figure S17: Base peak intensity LC traces with varying boronic acid loading.

5.6 Variation of water:acetonitrile ratio

Run as described in the general procedure varying the PBS:acetonitrile ratio (0, 10, 20, 30, 40, 50, or 100% MeCN) while maintaining reaction volume.

Entry	MeCN%	8a (%)	9a (%)
1	0%	10	89
2	10%	54	21
3	20%	89	5
4	30%	83	1
5	40%	69	2
6	50%	65	3
7	100%	0	1

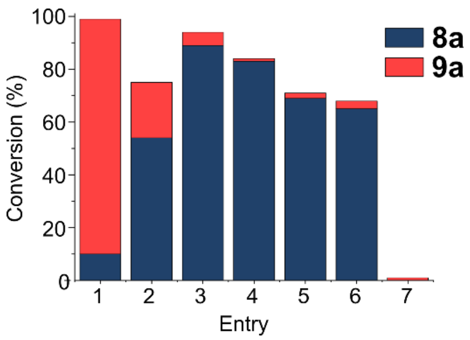

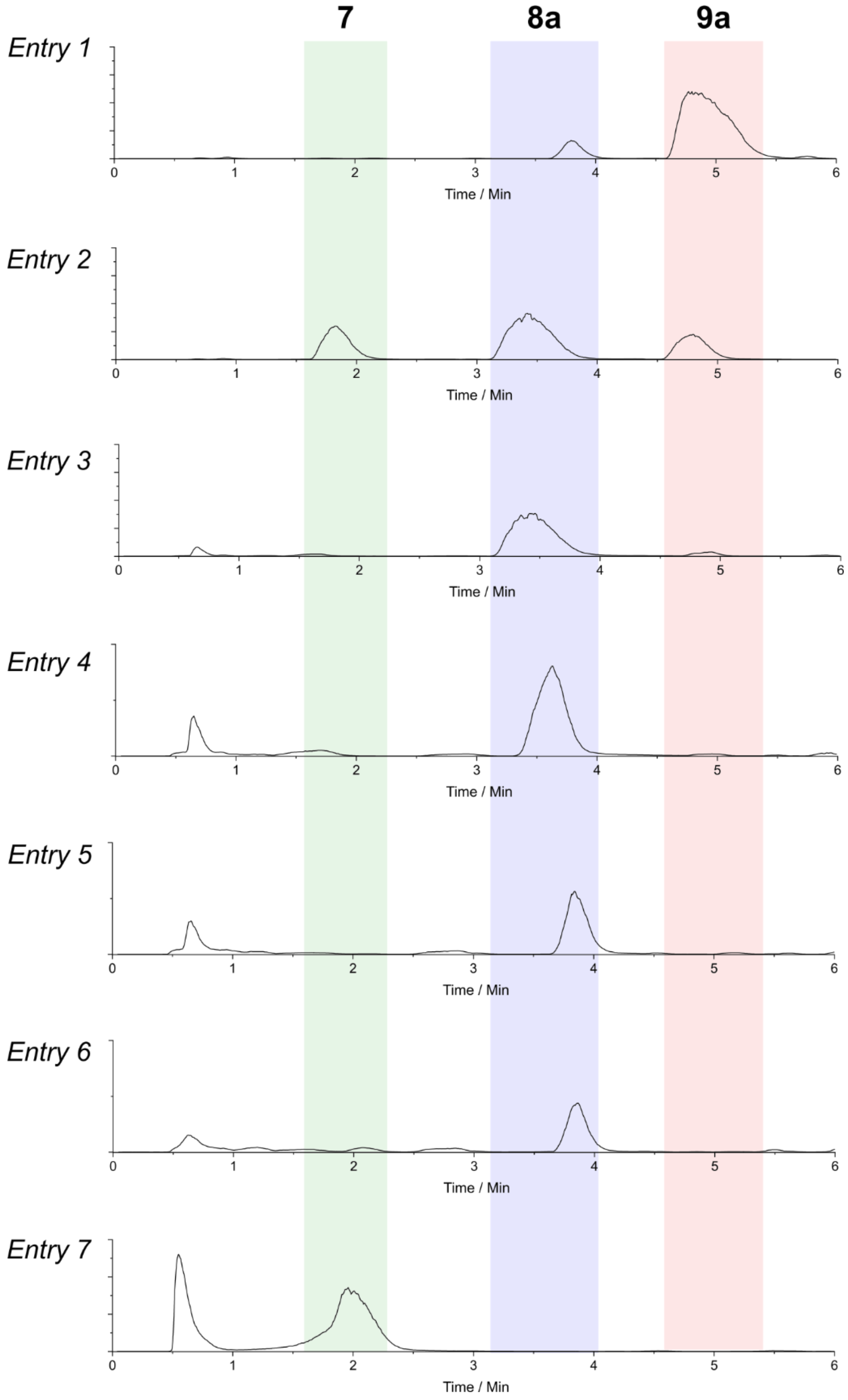

Figure S18: Base peak intensity LC traces with varying acetonitrile content.

5.7 Variation of boronic acid

Run as described in the general procedure with the boronic acids shown below.

Entry	Boronic acid	cSMCC (%)	SMCC (%)
1	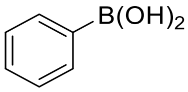	**8a,** 60	**9a,** 1
2	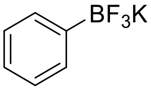	**8a,** 59	0
3	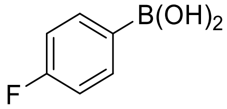	**8d,** 80	**9d,** 2
4	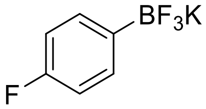	**8d,** 66	0
5	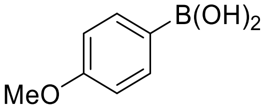	**8b,** 86	**9b,** 1
6	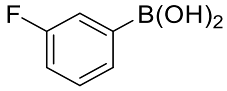	**8k,** 82	**9k,** 1
7	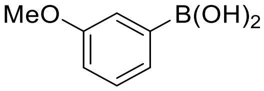	**8c,** 79	**9c,** 2
8	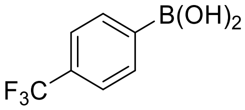	**8l,** 83	**9l,** 1
9	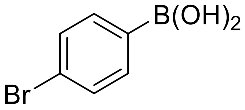	**8e,** 78	0
10	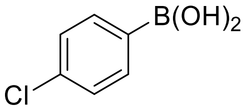	**8m,** 81	**9m,** 1
11	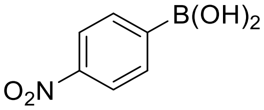	**8n,** 75	0
12	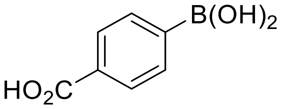	**8o,** 49	0
13	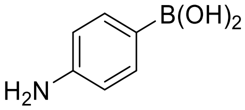	**8i,** 27	0
14	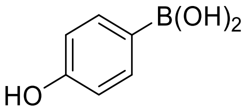	**8h,** 81	0
15	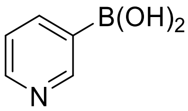	**8g,** 0	0

16	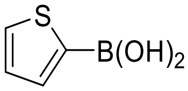	**8f,** 49	**9f,** 8
17	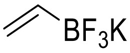	**8j,** 35	**9j,** 37

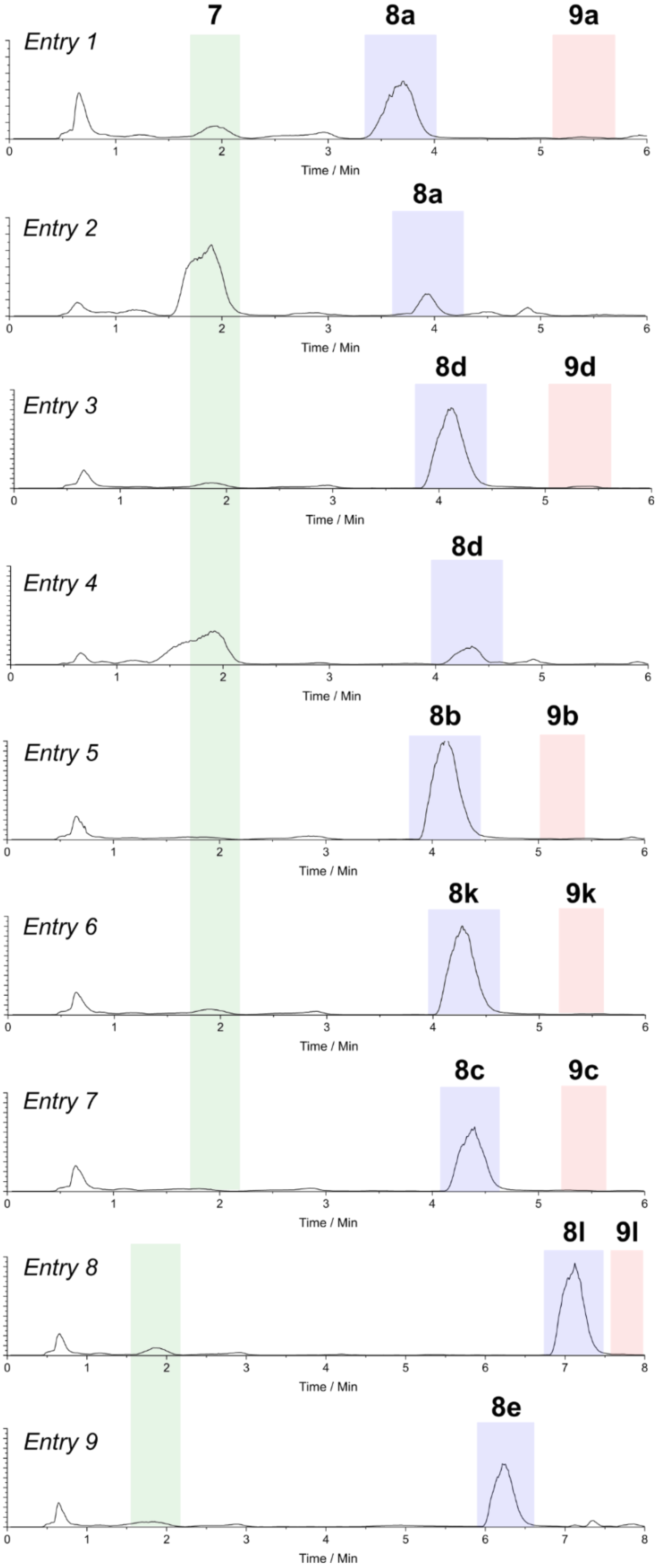

Figure S19: Base peak intensity LC traces with varying boronic acid.

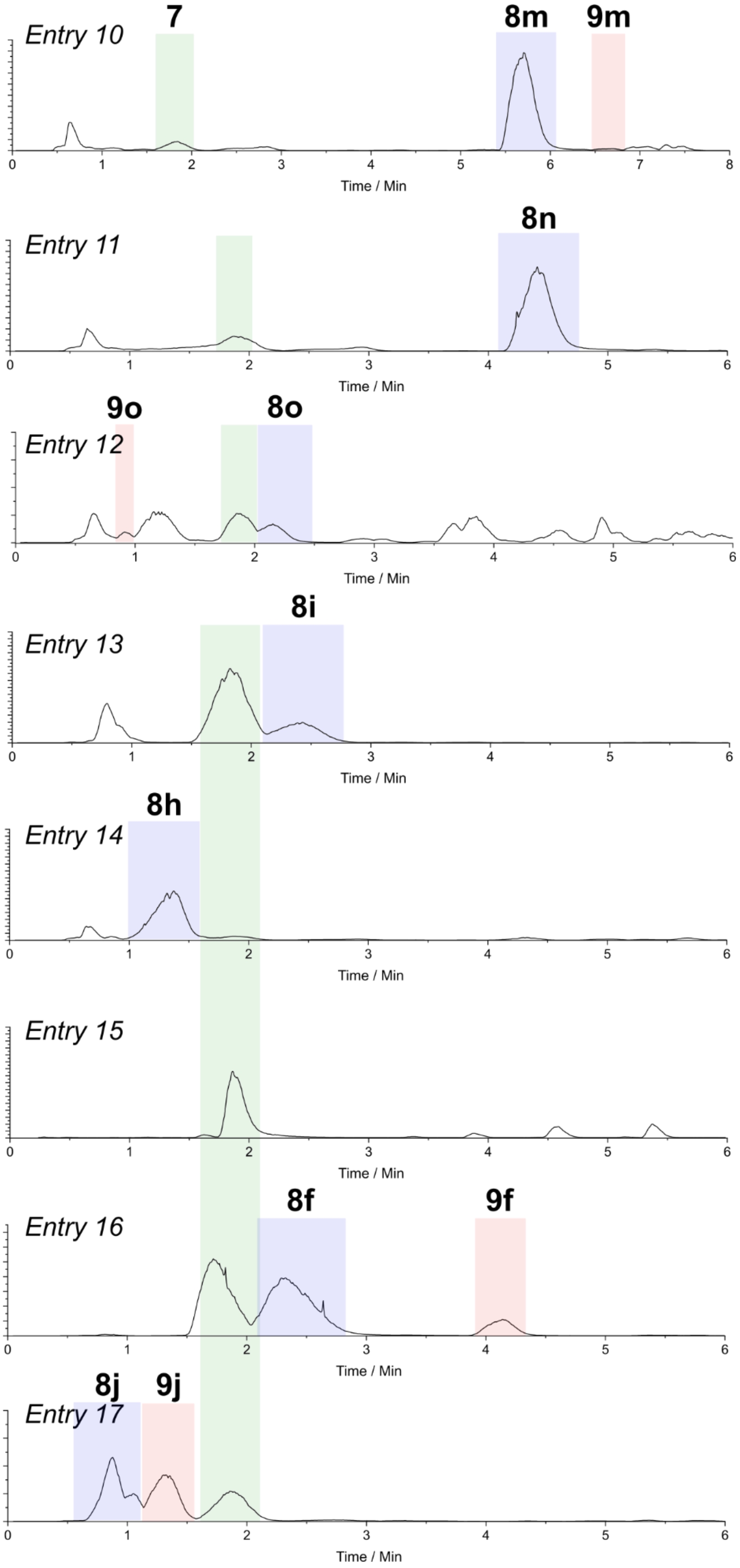
Entry 10
7
8m
9m
Time / Min
Entry 11
8n
Time / Min
Entry 12
9o
8o
Time / Min
Entry 13
8i
Time / Min
Entry 14
8h
Time / Min
Entry 15
Time / Min
Entry 16
8f
9f
Time / Min
Entry 17
8j
9j
Time / Min

5.8 Variation of buffer

Run as described in the general procedure in the buffers (all at pH 8) shown below. Reactions were run at r.t. to allow differences. between experiments to be observed.

Entry	Buffer	8a (%)	9a (%)
1	K^+ phosphate (500 mM)	73	1
2	PBS (500 mM)	60	1
3	HEPES (500 mM)	55	1
4	TAPS (500 mM)	56	4
5	Borate (pH 9, 500 mM)	28	7

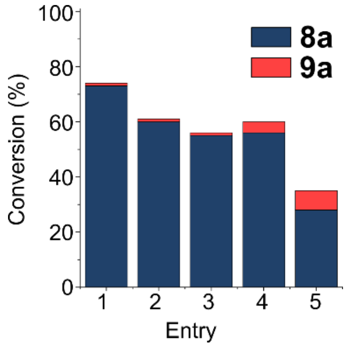

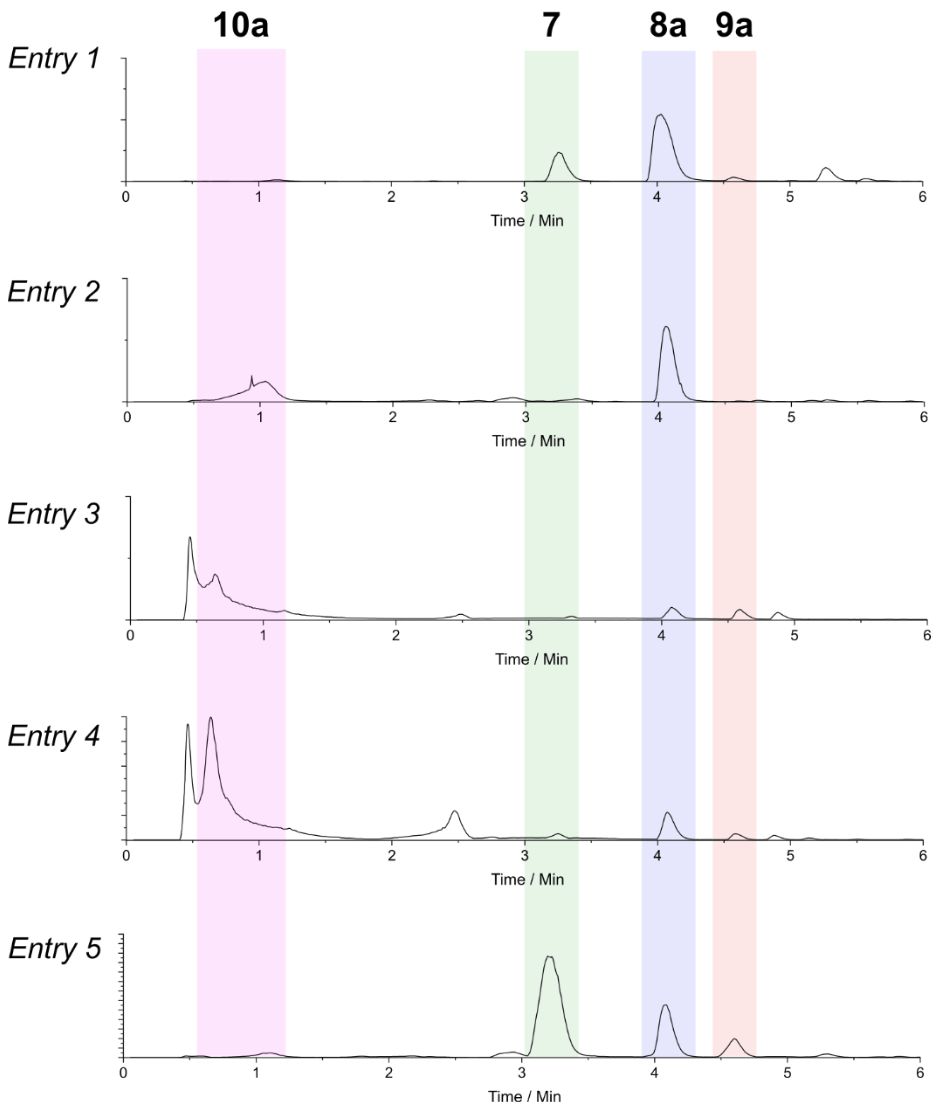

Figure S20: Base peak intensity LC traces with varying buffer.

5.9 Variation of temperature

Run as described in the general procedure varying the reaction temperature as shown below.

Entry	Temperature/ °C	8a (%)	9a (%)
1	5	6	5
2	10	24	2
3	20	29	0
4	25	65	0
5	30	62	5
6	35	56	2
7	40	81	17

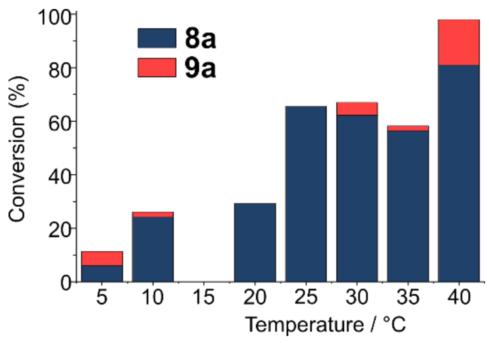

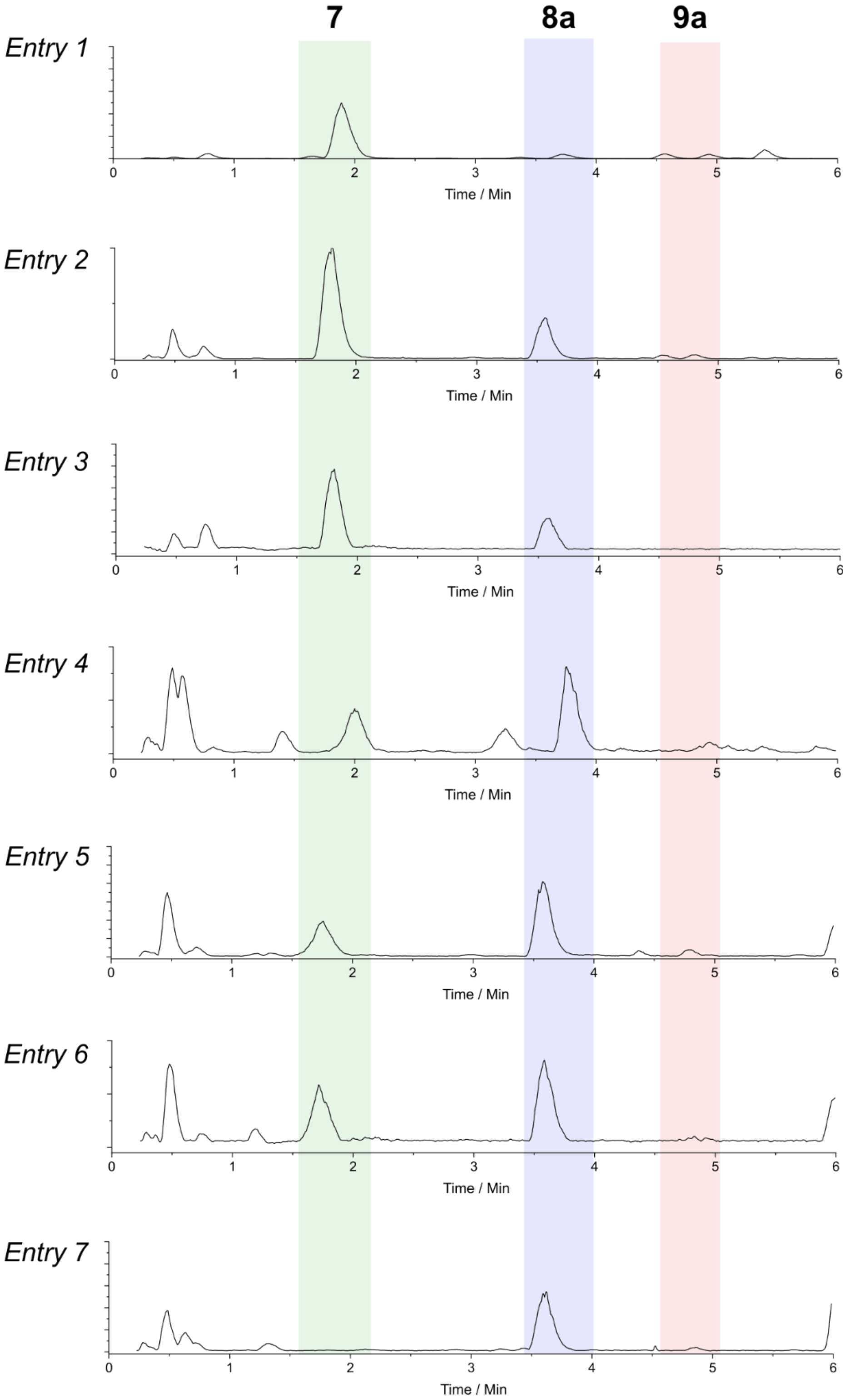

Figure S21: Base peak intensity LC traces with varying temperature.

5.10 Timescreen

Run as described in the general procedure on a ×10 scale. At intervals of 15-120 min 40 µL aliquots were removed and added to a solution of cysteine hydrochloride (0.4 M, 5 µL, 10 equiv. w.r.t. peptide) in PBS (pH 8.2) to quench the reaction. Relative

intensities of the base peak chromatogram for the starting peptide **7** and cSMCC product **8a** were plotted as a function of time

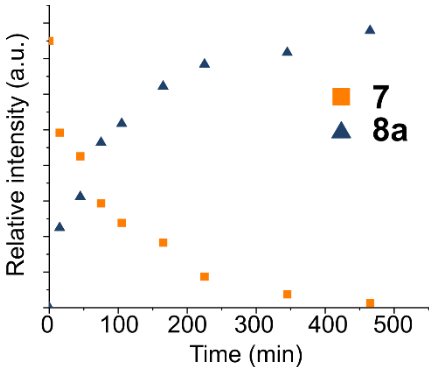

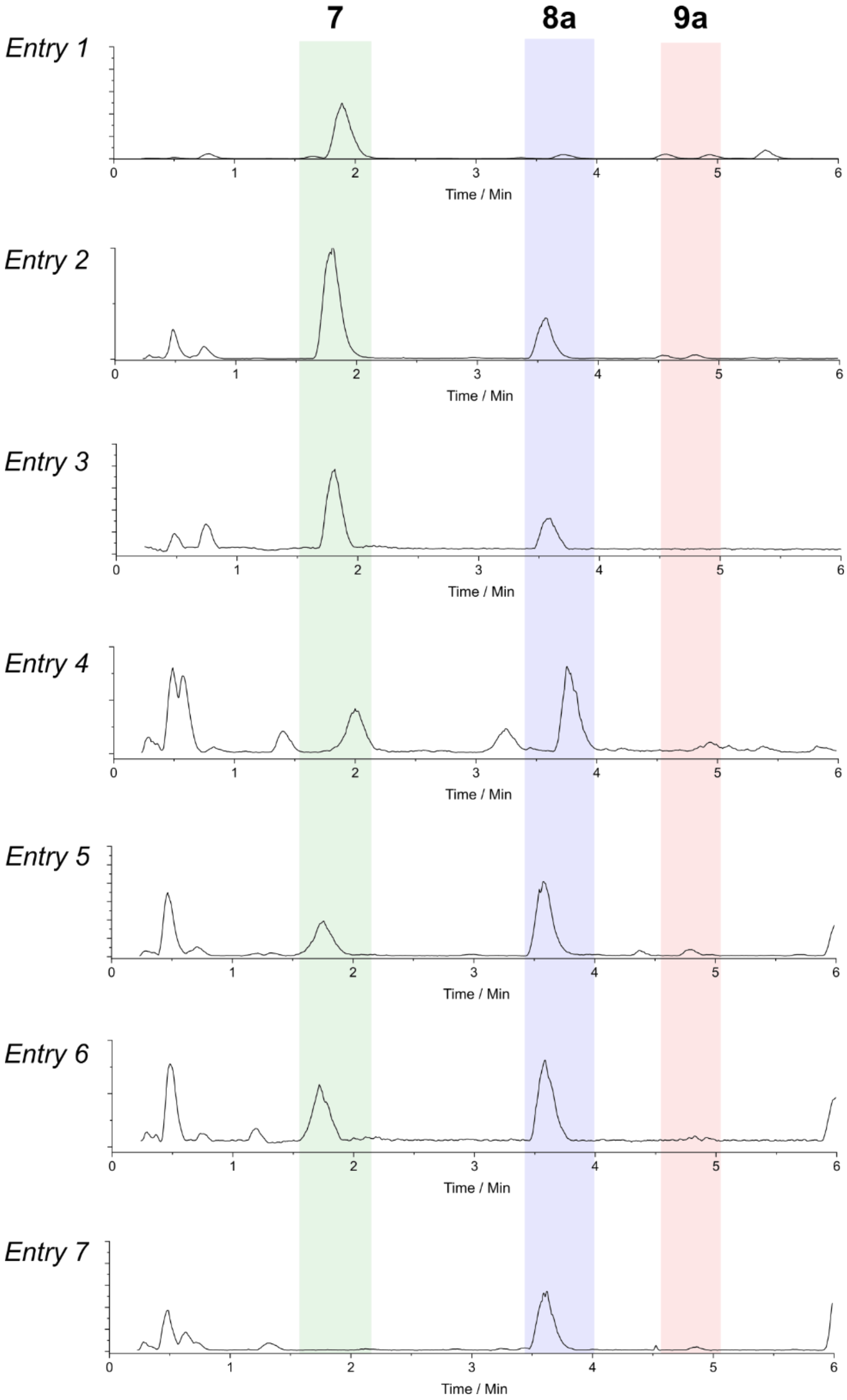

Figure S22: Base peak intensity LC traces after varying reaction time.

5.11 Alternative peptide substrates

Run as described in the general procedure with the peptides shown below.

Entry	Peptide	cSMCC (%)	SMCC (%)
1	H_2N-AEQRE-***p*IPhe**-LERKARESA-$CONH_2$ **20**	**12,** 56	2
2	H_2N-SYSMEH-***p*IPhe**-RYG-$CONH_2$ **22**	**14,** 89	0
3	H_2N-SYSMEH-***p*IPhe**-RYG-$CONH_2$ **22**	**14,** 91	0
4	H_2N-CDPGYIGSR-***p*IPhe**-$CONH_2$ **15**	0	0
5	H_2N-LP-**3ITyr**-TG-$CONH_2$ **23**	**16,** 28	1
6	H_2N-SL-***p*BrPhe**-RAG-CO_2H **25**	0	0

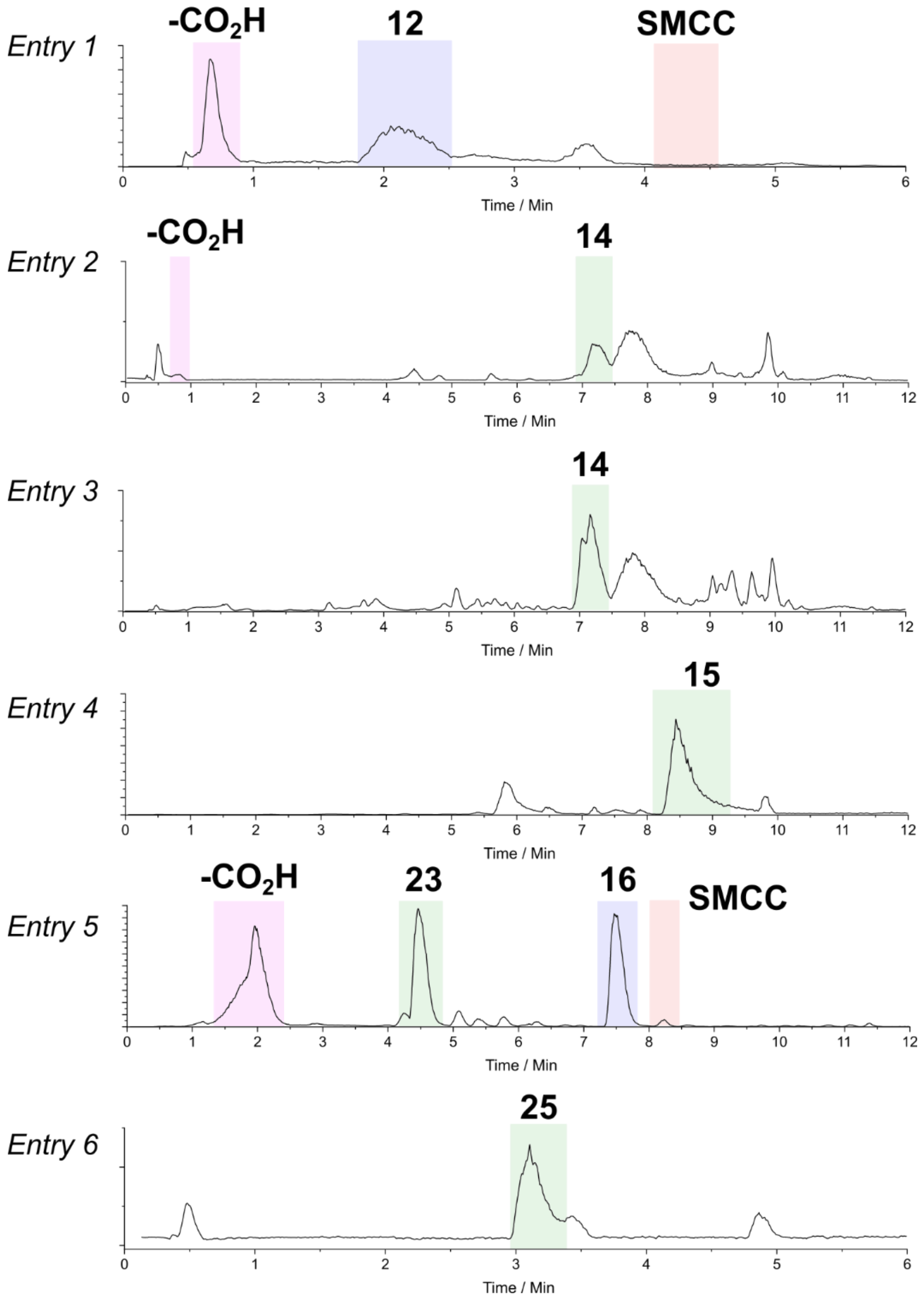

Figure S23: Base peak intensity LC traces with varying peptide.

5.12 Reduction with $NaBH_4$

Following completion of the reaction run as described above, the mixture was centrifuged (10 min, 10,000 rpm) and the supernatant collected. Sodium borohydride was then added and the mixture incubated at r.t. for 1 h. The mixture was then directly analysed by LC-MS, which showed clean reduction of the diaryl ketone to the corresponding alcohol.

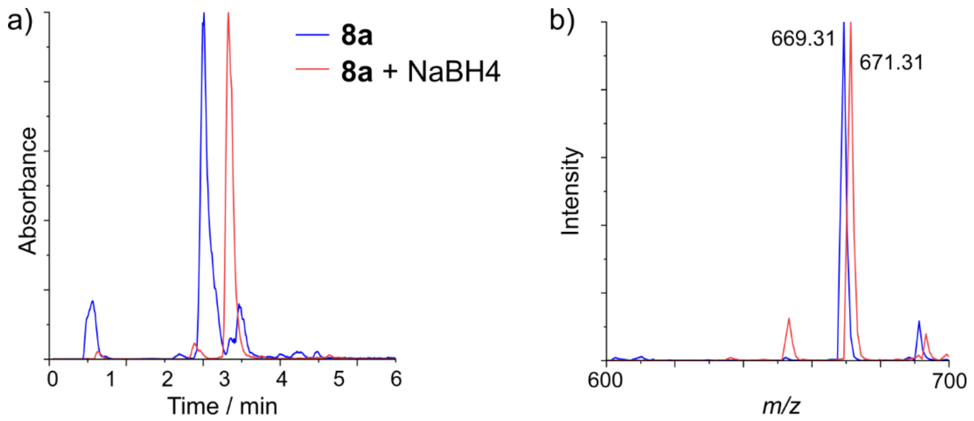

Figure S24: a) LC trace of reaction mixture before (blue) and after (red) treatment with sodium borohydride, BPC; c) Mass spectrum of **8a**.

6. Peptide iodination with Barluenga's reagent

Barluenga's reagent (IPy_2BF_4, 87 mg, 233 µmol) was added to a solution of tyrosine-containing peptide LPYTG **33** (85 mg, 155 µmol) in a mixture of trifluoroacetic acid (1 mL) and DCM (10 mL). The mixture was stirred for 30 min then reduced to ~2 mL in volume. The remaining solution was added dropwise to ice-cold diethyl ether (50 mL), and the resultant precipitate collected by centrifugation (2 × 10 min, 3000 rpm). The pellet was air-dryed for 5 min then resuspended in water (20 mL) and lyophilised. LC-MS analysis revealed 91% conversion to the mono-iodinated peptide **23**, 1% di-iodinated. The product was used directly in subsequent cSMCCs.

7. Large scale peptide modification

General cSMCC procedure: A microcentrifuge tube was charged with peptide (19 µmol, 1 equiv.) and $Mo(CO)_6$ (10 mg, 38 µmol, 2 equiv.), and stock solutions of boronic acid (152 µmol, 8 equiv.) in acetonitrile (800 µL) and 'Pd(TFA)$_2$' (2.5 mg, 7.6 µmol, 0.4 equiv) in potassium phosphate buffer (1200 µL, 250 mM, pH 8.0) were sequentially added. The tube was then sealed and incubated at 37 °C (1000 rpm) for 24 h. After cooling to r.t., the mixture was centrifuged (10 min, 10,000 rpm) and the supernatant collected. The pellet was resuspended in water (1000 µL) and then re-centrifuged (10 min, 10,000 rpm). The supernatants were combined, SiliaMetS DMT (~16 mg) palladium scavenger beads were added, and the mixture stirred in the dark for 2 h.

The mixture was then filtered through a 0.45 µm filter and the solution purified either via automated reverse-phase flash column chromatography, as described in Section 1, or using a using a Supelco Discovery DSC-18 SPE tube (bed wt. 5g, vol. 20 mL). The SPE cartridge was conditioned in 90% methanol in water, equilibrated in water + 0.1% TFA (*v/v*), and the peptide loaded in a minimum volume of water + 0.1% TFA (*v/v*). The peptide was eluted using a stepwise gradient of acetonitrile in water (0.1% TFA *v/v*), starting at either 2.5 or 5% MeCN, increasing in increments of 2.5 or 5% depending on the retention time of the peptide as observed by LC-MS. Fractions containing the peptide were combined, the organic fraction removed *in vacuo*, and the remaining aqueous solution removed via lyophilisation to provide the pure peptide.

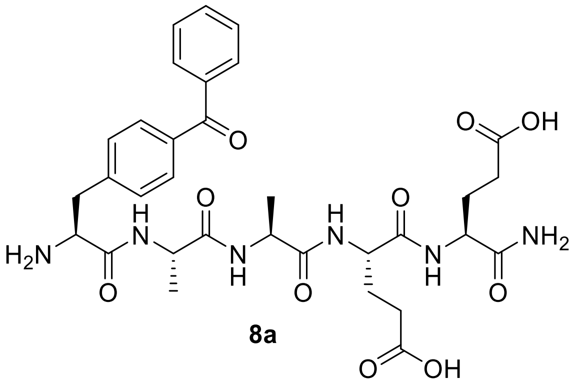

8a

Yield of 10.4 mg, 15.6 µmol (81%).

HRMS (ESI$^+$): *m/z* Calcd for $C_{32}H_{41}N_6O_{10}^+$: 669.2879 [M+H]$^+$; Observed: 669.2912.

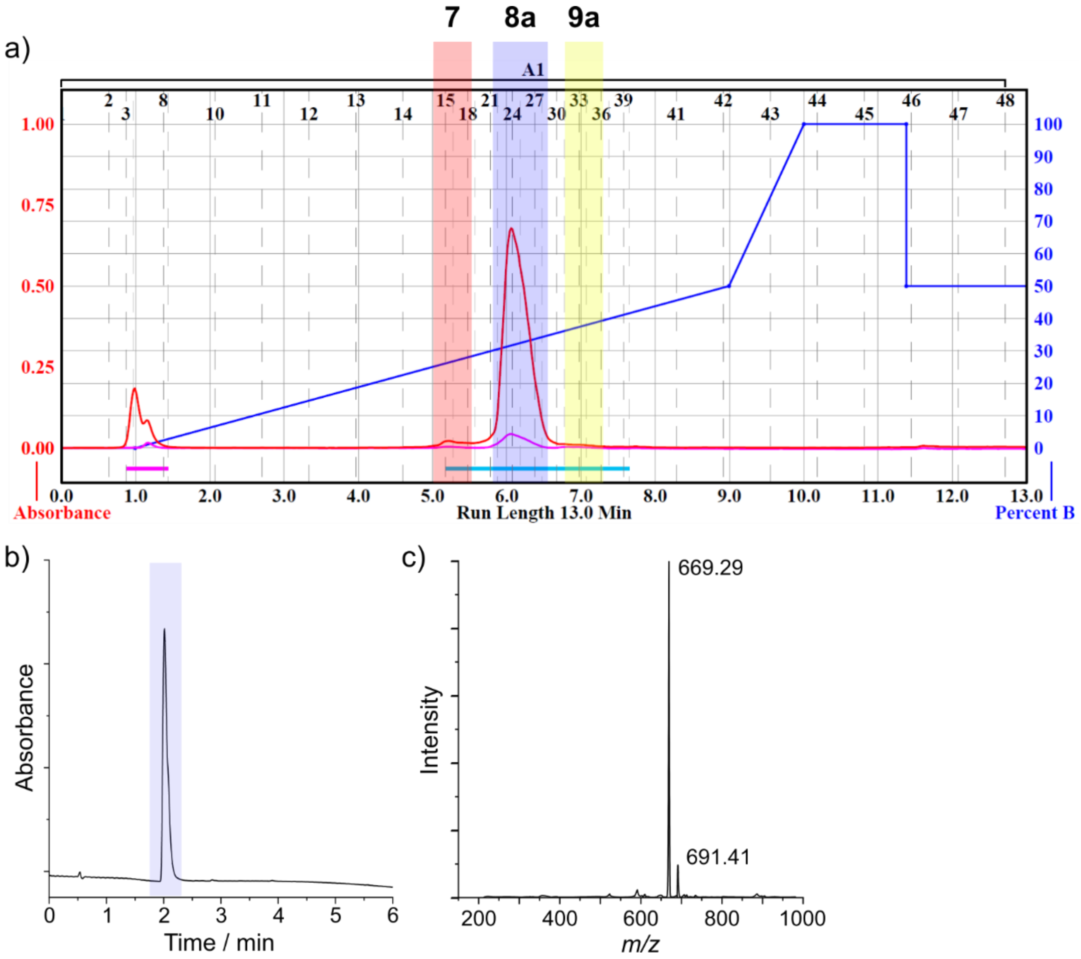

Figure S25: a) Reverse-phase chromatography trace, with absorbance monitored at 280 nm; b) LC trace of **8a** after purification, absorbance at 280 nm; c) Low resolution mass spectrum ([M+H]$^+$) of **8a**.

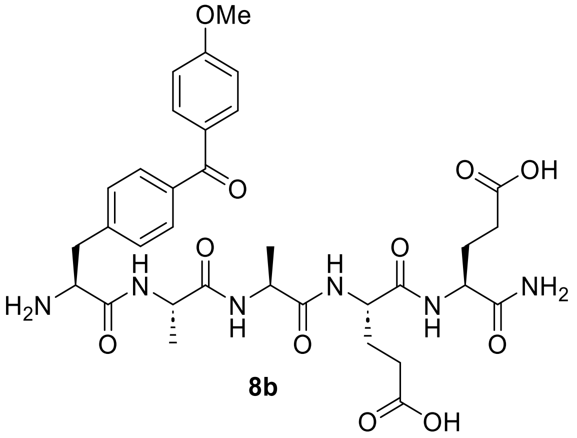

8b

Yield of 5.2 mg, 7.4 µmol (34%).

HRMS (ESI$^+$): *m/z* Calcd for $C_{33}H_{42}N_6NaO_{11}^+$: 721.2804 [M+Na]$^+$; Observed: 721.2794.

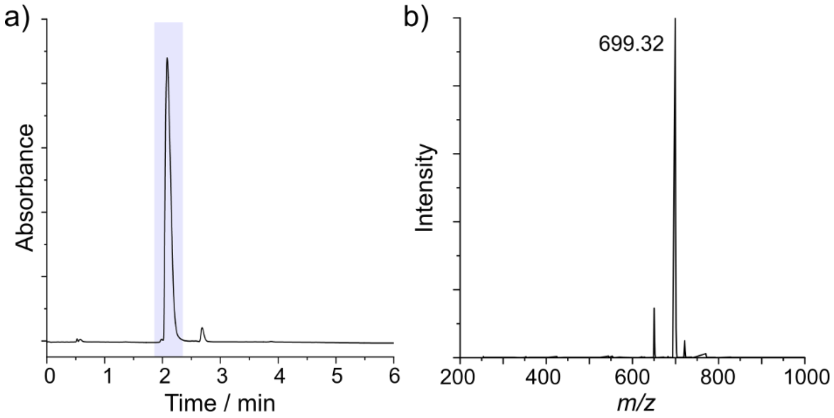

Figure S26: a) LC trace of **8b** after purification, absorbance at 280 nm; b) Low resolution mass spectrum ([M+H]$^+$) of **8b**.

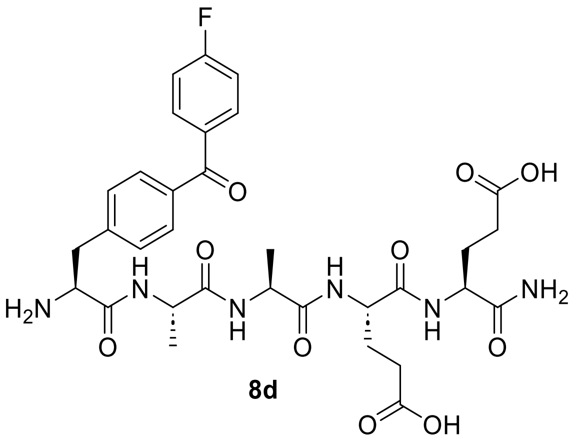

8d

Yield of 4.8 mg, 7.0 µmol (32%).

HRMS (ESI$^+$): *m/z* Calcd for $C_{32}H_{39}FN_6NaO_{10}^+$: 709.2604 [M+Na]$^+$; Observed: 709.2616.

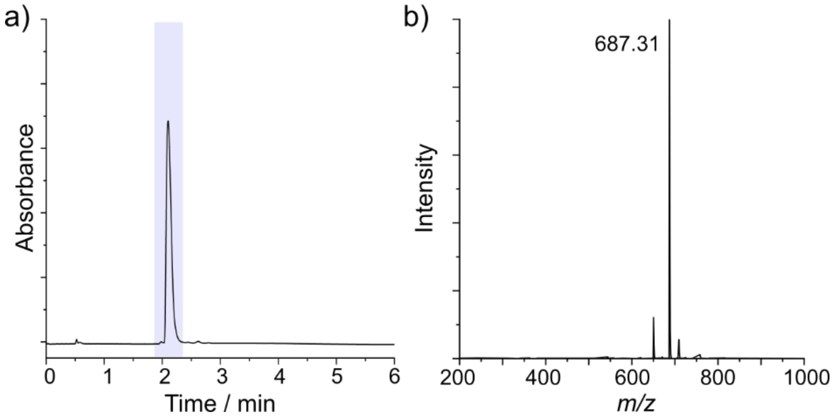

Figure S27: a) LC trace of **8d** after purification, absorbance at 280 nm; b) Low resolution mass spectrum ([M+H]$^+$) of **8d**.

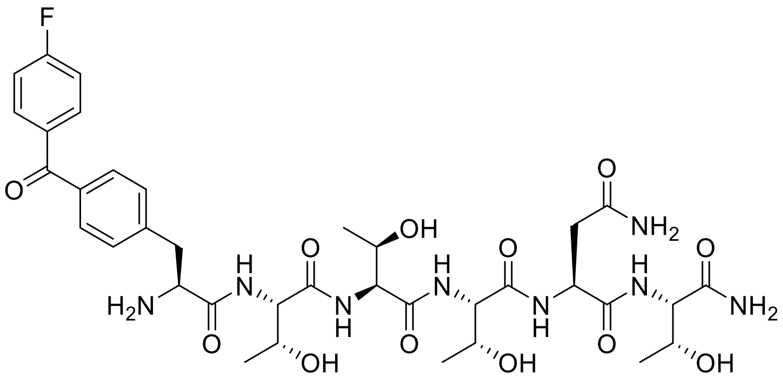

11

Yield of 5.4 mg, 6.7 µmol (36%).

HRMS (ESI+): *m/z* Calcd for $C_{36}H_{50}FN_8O_{12}^+$: 805.3527 $[M+H]^+$; Observed: 805.3553.

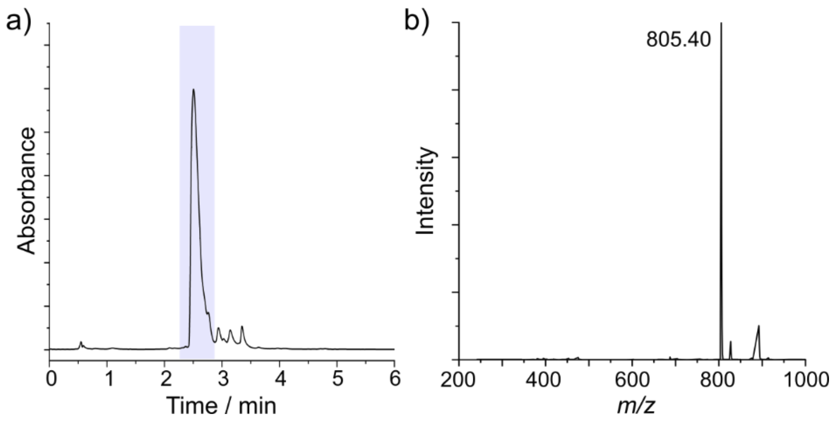

Figure S28: a) LC trace of **11** after purification, absorbance at 280 nm; b) Low resolution mass spectrum ($[M+H]^+$) of **11.**

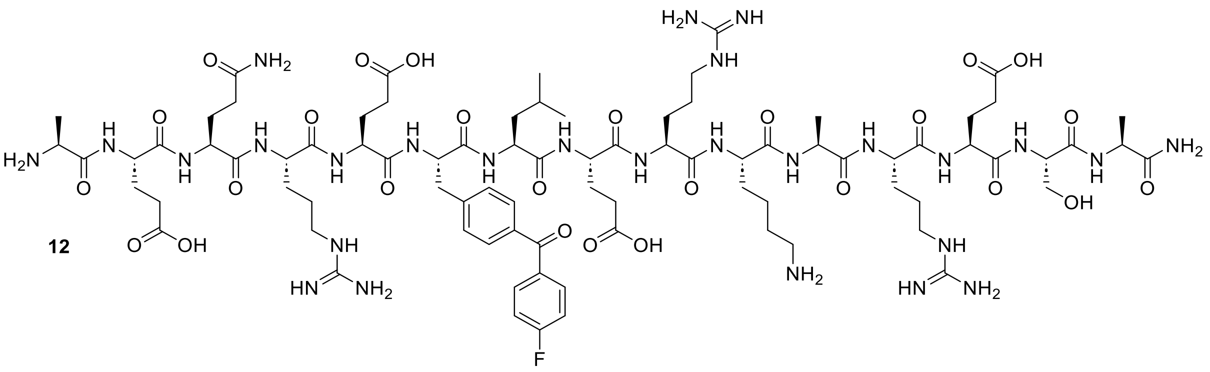

Yield of 4.9 mg, 2.5 µmol (23%).

LRMS (ESI$^+$): *m/z* Calcd for $C_{83}H_{133}FN_{27}O_{26}^{3+}$: 647.66 [M+3H]$^+$; Observed: 647.97.

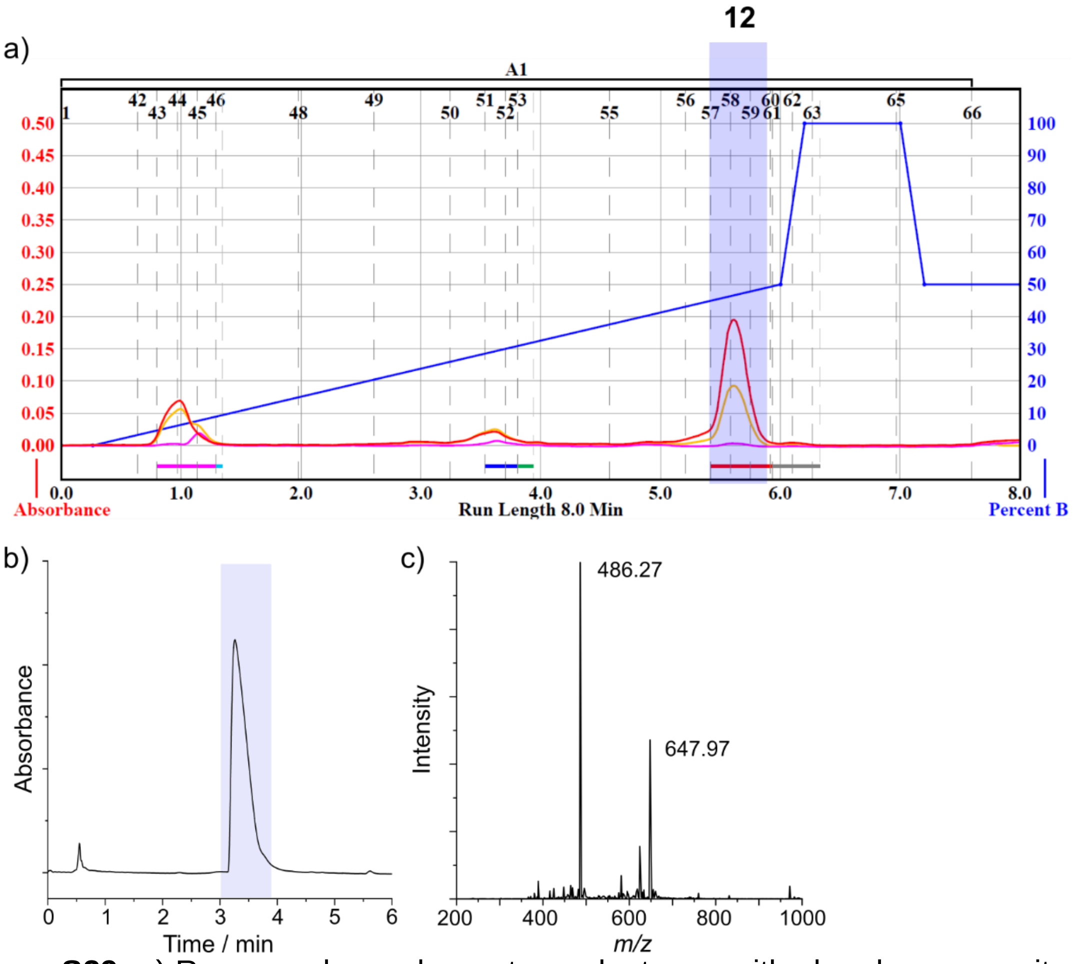

Figure S29: a) Reverse-phase chromatography trace, with absorbance monitored at 280 nm; b) LC trace of **12** after purification, absorbance at 280 nm; c) Low resolution mass spectrum ([M+3H]$^{3+}$, [M+4H]$^{4+}$) of **12**.

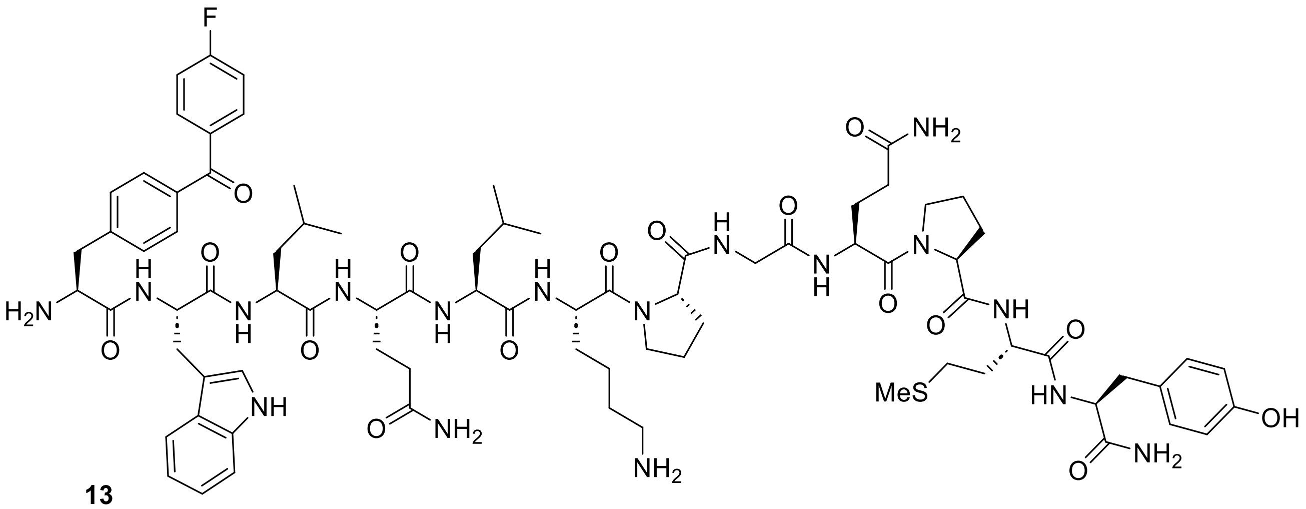

13

Yield of 8.4 mg, 5.2 µmol (66%).

HRMS (ESI$^+$): *m/z* Calcd for $C_{81}H_{111}FN_{17}O_{16}S^+$: 1628.8094 [M+H]$^+$; Observed: 1628.8117.

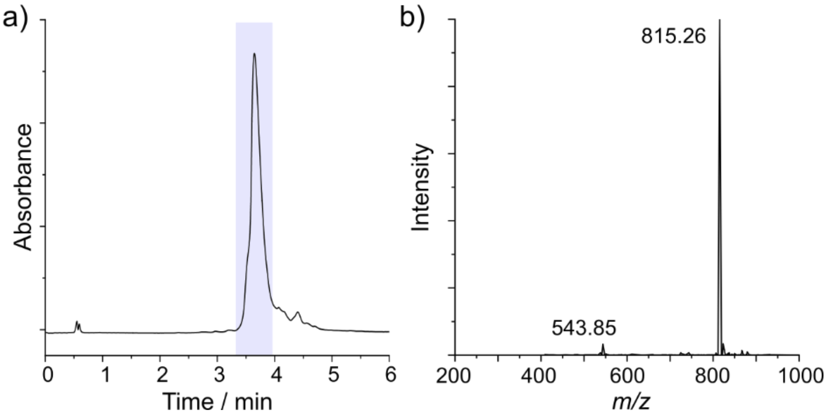

Figure S30: a) LC trace of **13** after purification, absorbance at 280 nm; b) Low resolution mass spectrum ([M+2H]$^{2+}$) of **13**.

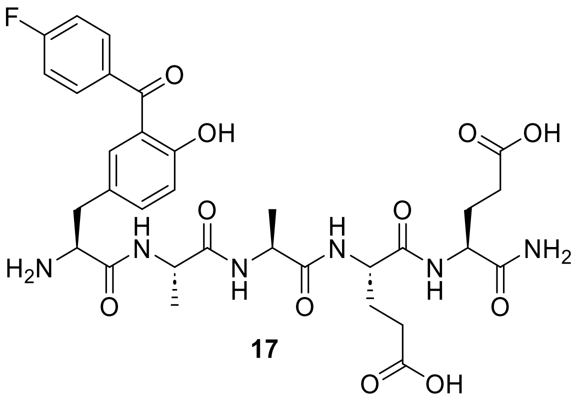

17

Yield of 8.1 mg, 11.6 µmol (61%).

HRMS (ESI$^+$): *m/z* Calcd for $C_{32}H_{38}FN_6O_{11}^+$: 703.2588 [M+H]$^+$; Observed: 703.2581.

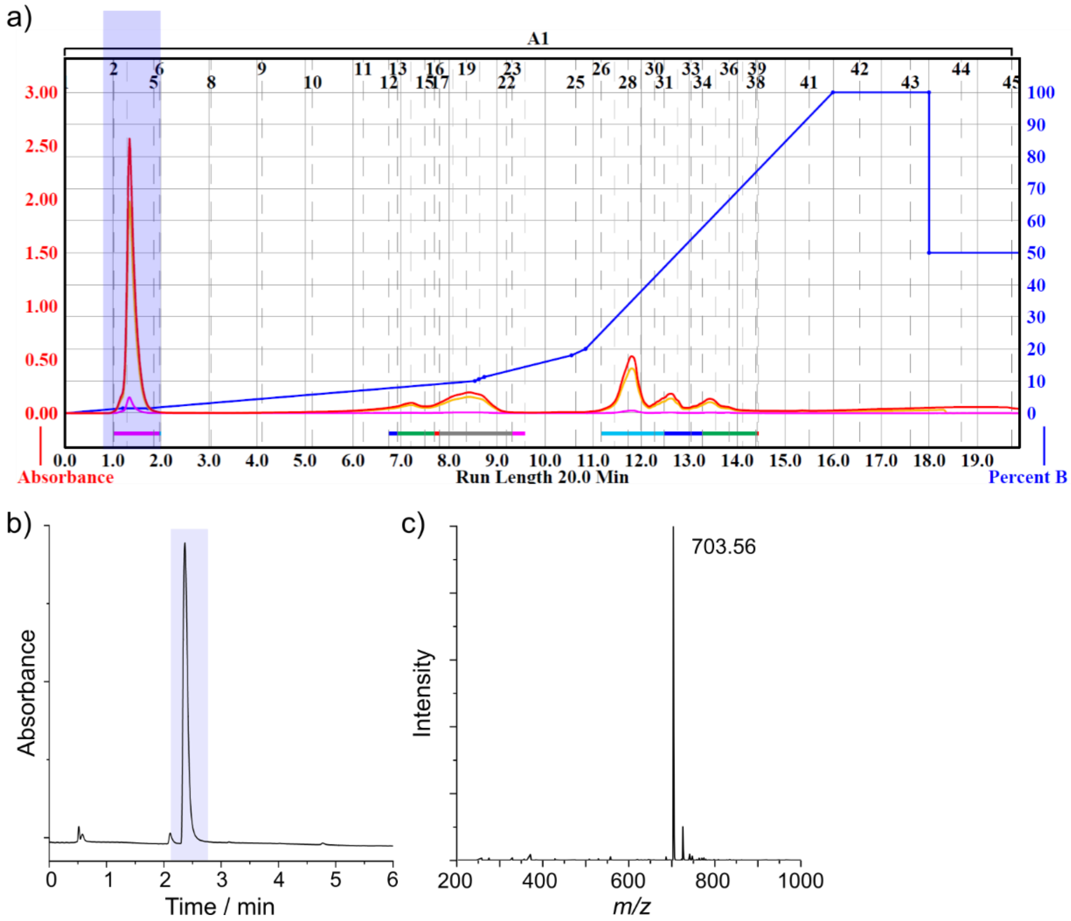

Figure S31: a) Reverse-phase chromatography trace, with absorbance monitored at 280 nm; b) LC trace of **17** after purification, absorbance at 280 nm; c) Low resolution mass spectrum ([M+H]$^+$) of **17**.

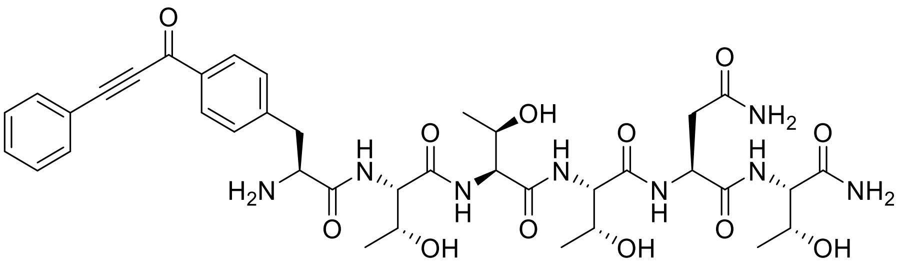

18

Phenylacetylene was used in place of the phenylboronic acid Yield of 10.9 mg, 13.5 µmol (71%).

HRMS (ESI$^+$): *m/z* Calcd for $C_{38}H_{50}N_8NaO_{12}^+$: 833.3440 [M+Na]$^+$; Observed: 833.3476.

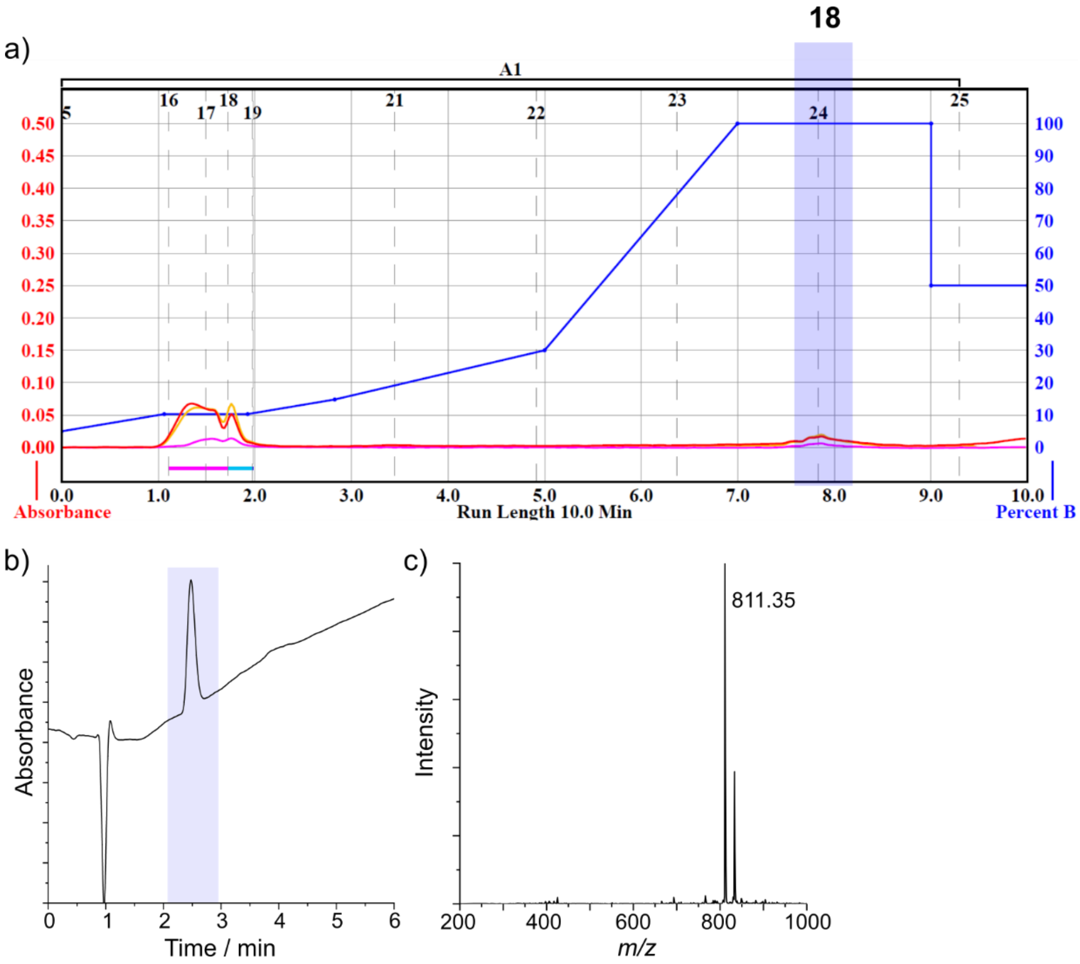

Figure S32: a) Reverse-phase chromatography trace, with absorbance monitored at 220 nm; b) LC trace of **18** after purification, absorbance at 220 nm; c) Low resolution mass spectrum ([M+H]$^+$) of **18**.

8. Peptide MS/MS analysis

Peptides with a molecular weight of >1500 Da were subject to further characterisation by MS/MS analysis.

A 1 µL aliquot of each peptide was applied directly to the ground steel MALDI target plate, followed immediately by an equal volume of a freshly-prepared 5 mg mL^{-1} solution of 4-hydroxy-α-cyano-cinnamic acid in 50% aqueous (v/v) acetonitrile containing 0.1% trifluoroacetic acid (v/v). Positive-ion MALDI mass spectra were obtained using a Bruker UltrafleXtreme in reflectron mode, equipped with a Nd:YAG smart beam laser. MS spectra were acquired over a mass range of *m/z* 800-4000. Final mass spectra were externally calibrated against an adjacent spot containing 6 peptides of known mass. For each spot the ten most intense ions, with S/N greater than 8, were selected for fragmentation, which was performed in LIFT mode without the introduction of a collision gas.

Bruker FlexAnalysis software (version 3.4) was used for spectral processing and peak list generation. Monoisotopic masses were obtained using a SNAP averaging algorithm (C 4.9384, N 1.3577, O 1.4773, S 0.0417, H 7.7583) and a S/N threshold of 2 for MS and 6 for MS^2. Tandem mass spectral data searched against a fasta database using a locally-running copy of the Mascot program (Matrix Science Ltd., version 3.0), through the Mascot Daemon interface (version 2.8).

Search criteria specified: Enzyme, NoCleave; Fixed modifications, None; Variable modifications, Oxidation (M); Amidated C-terminus; 4-iodo-phenylalanine; 4-(4-fluorobenzoyl) phenylalanine. Peptide tolerance, 100 ppm; MS/MS tolerance, 0.5 Da; Instrument, MALDI-TOF-TOF. Peptide matches were filtered to require an expect score of 0.05 or lower.

p-Iodophenylalanine residues within the sequences are annotated as ^{F}F. *p*-Fluoro-cSMCC labelled residues within the sequences are annotated as ^{FL}F.

RuBisCO binding domain peptide 20

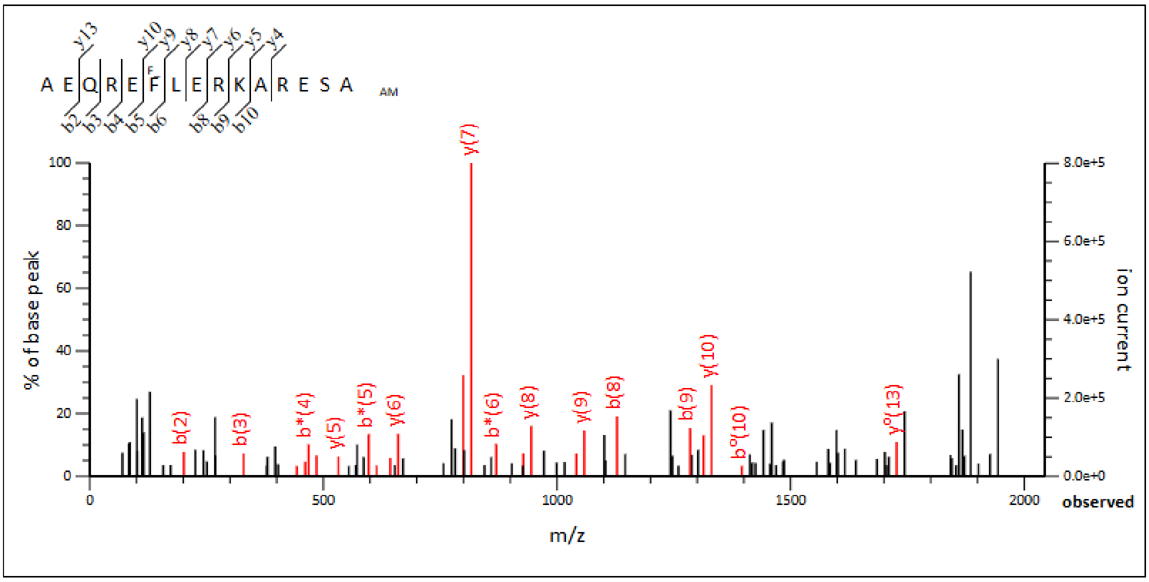

RuBisCO binding domain cSMCC product 12

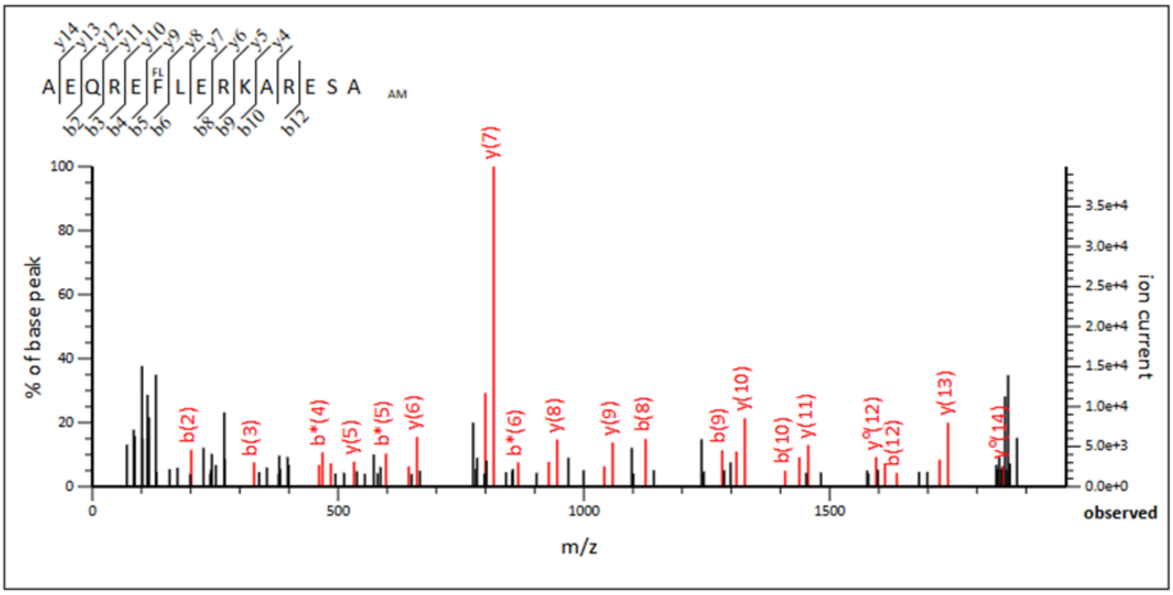

Alpha factor 21

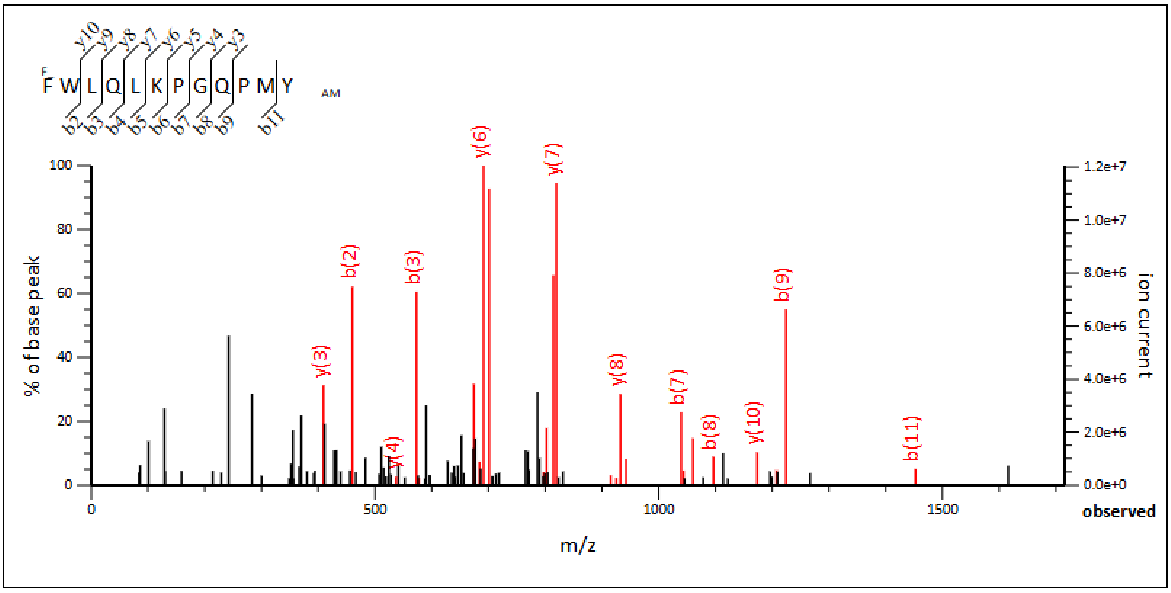

Alpha factor cSMCC product 13

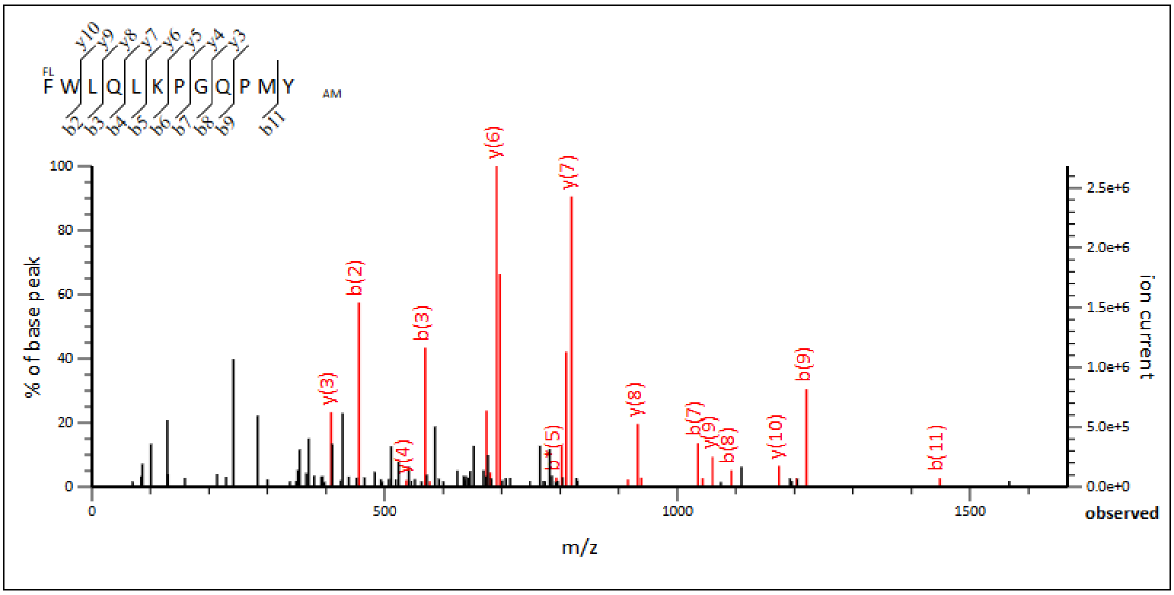

9. References

1 W. A. Carole and T. J. Colacot, *Chem. – Eur. J.*, 2016, **22**, 7686–7695.

2 C. D. Spicer and B. G. Davis, *Chem. Commun.*, 2011, **47**, 1698–1700.

3 F. Debaene, J. A. Da Silva, Z. Pianowski, F. J. Duran and N. Winssinger, *Tetrahedron*, 2007, **63**, 6577–6586.

4 Rajput, Sunnia, McLean, Kirsty J, Poddar, Harshwardhan, Selvam, Irwin R, Nagalingam, Gayathri, Triccas, James A, Levy, Colin W, Munro, Andrew W, and Hutton, Craig A, *J. Med. Chem.*, 2019, **62**, 9792–9805.

5 T. J. M. Byrne, M. E. Mylrea and J. D. Cuthbertson, *Org. Lett.*, 2023, **25**, 2361–2365.

6 A. M. Steer, H. L. Bolt, W. D. G. Brittain and S. L. Cobb, *Tetrahedron Lett.*, 2018, **59**, 2644–2646.

10. Peptide NMRs and HRMS

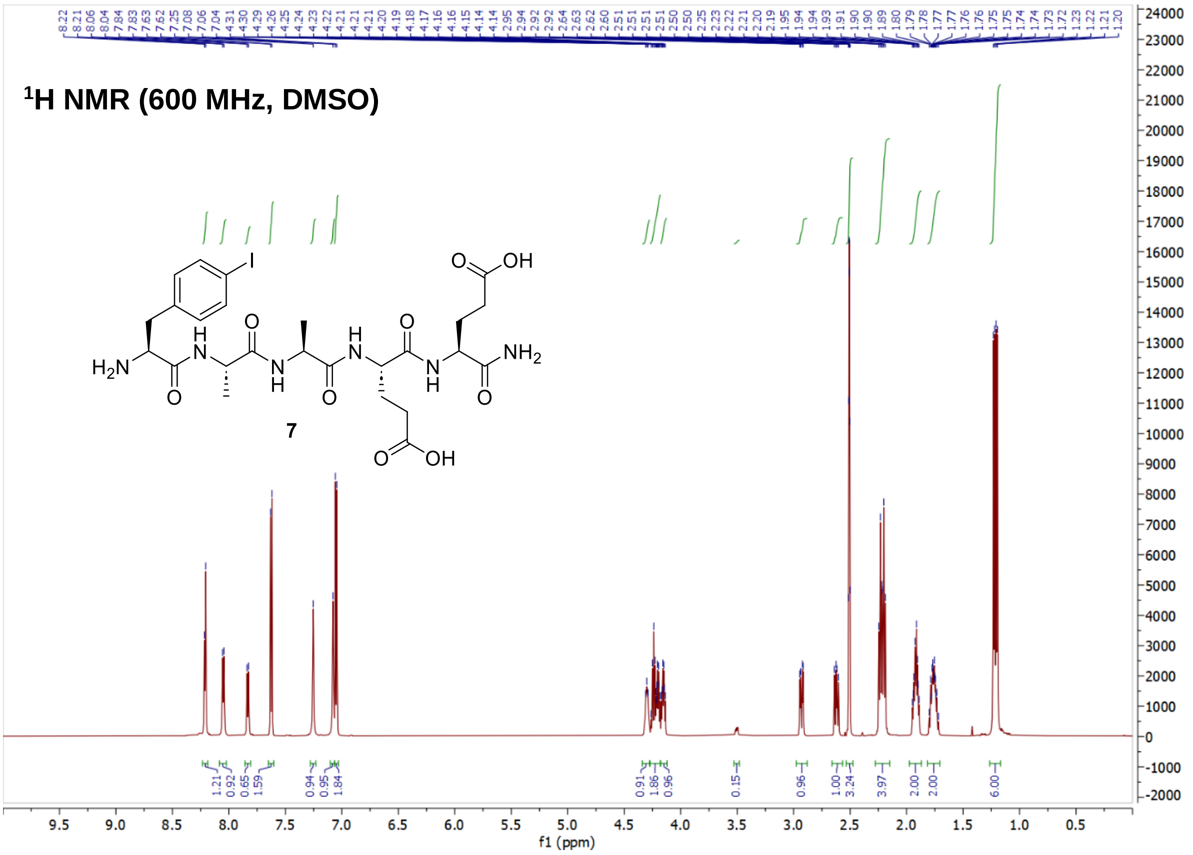

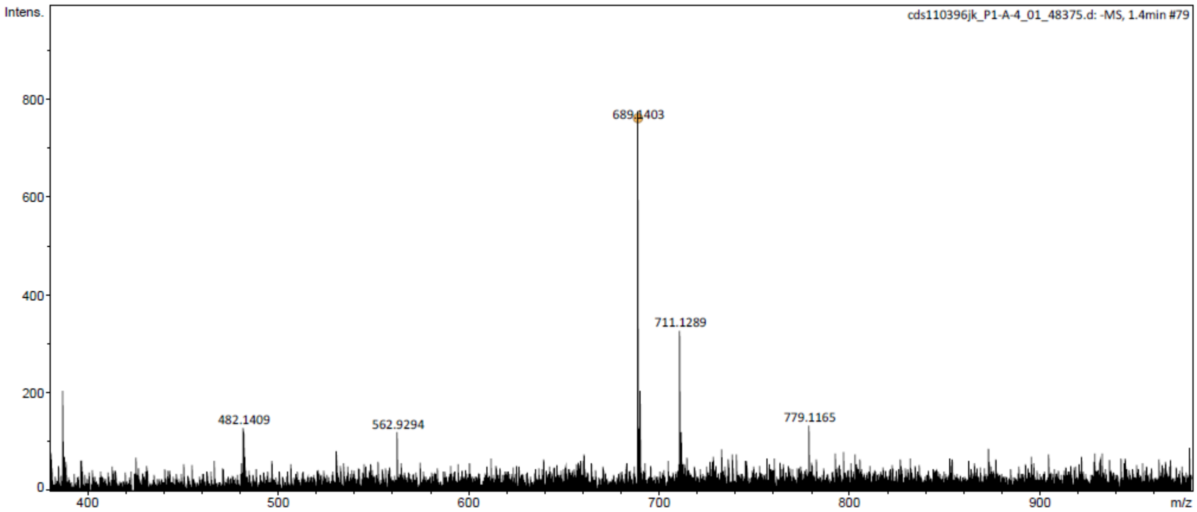

Meas. m/z	#	Ion Formula	m/z	err [ppm]	err [mDa]	mSigma	Mean err [ppm]
689.1403	1	C25H34IN6O9	689.1437	4.9	3.4	n.a.	2.2

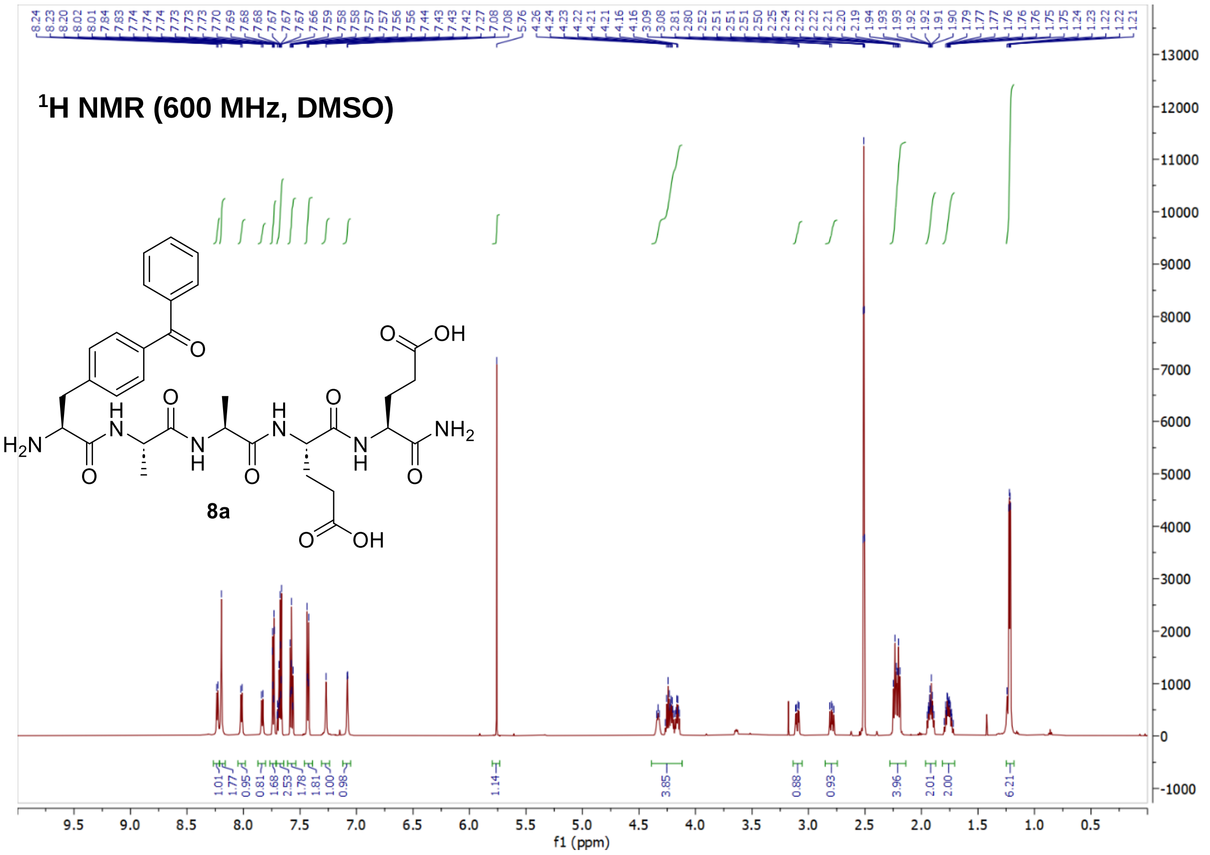

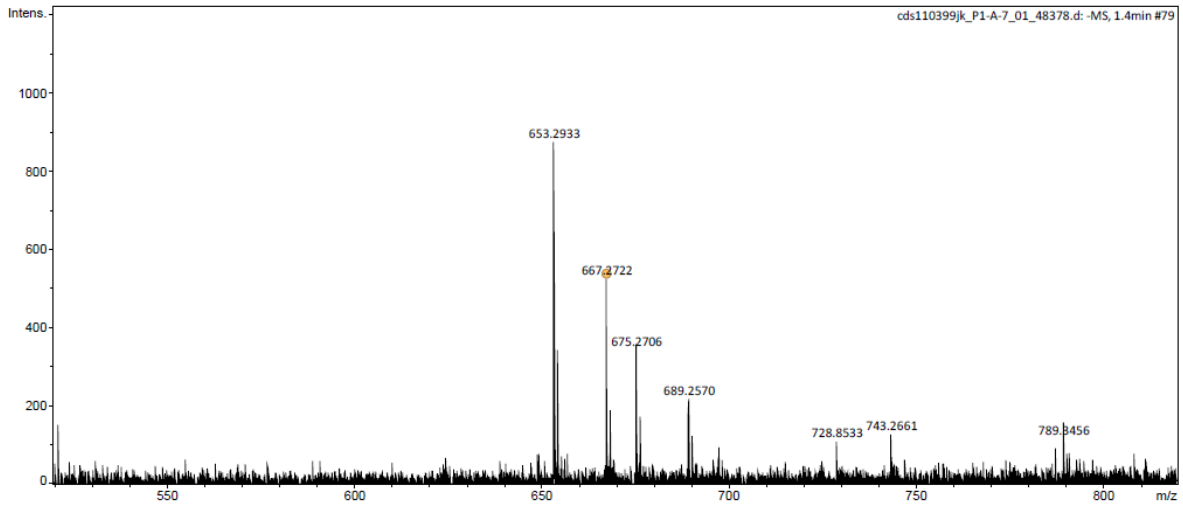

Meas. m/z	#	Ion Formula	m/z	err [ppm]	err [mDa]	mSigma	Mean err [ppm]
667.2722	1	C32H39N6O10	667.2733	1.7	1.1	46.0	1.0

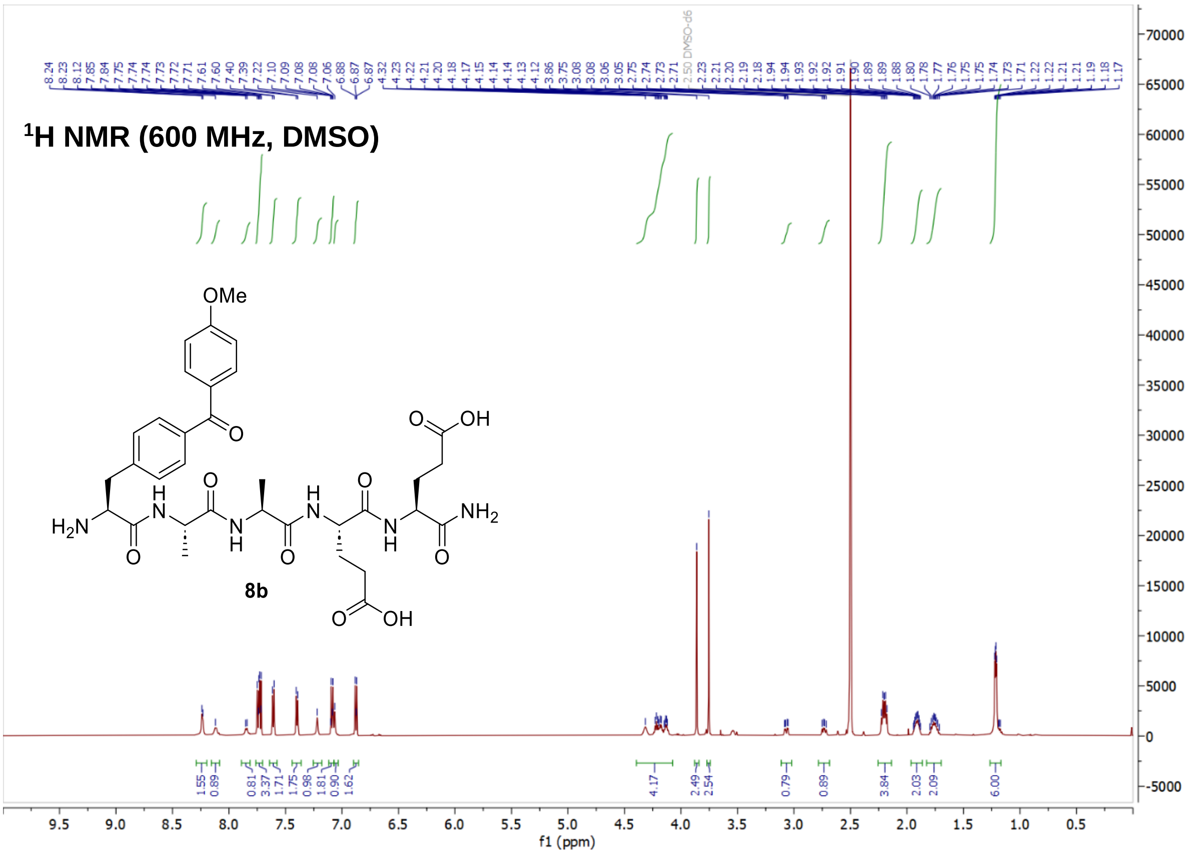

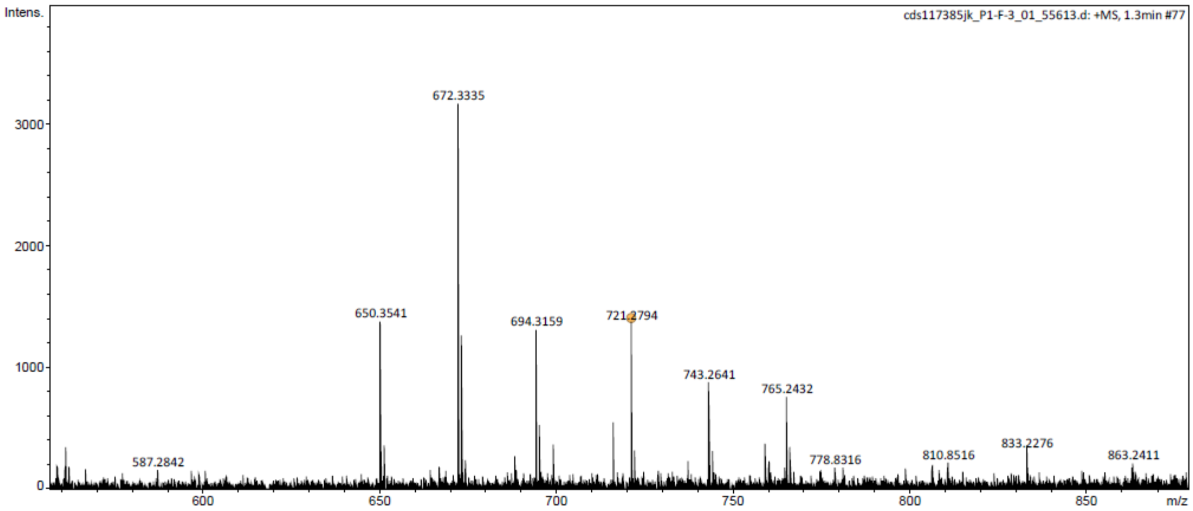

Meas. m/z	#	Ion Formula	m/z	err [ppm]	err [mDa]	mSigma	Mean err [ppm]
721.2794	1	C33H42N6NaO11	721.2804	1.4	1.0	91.4	-1.5

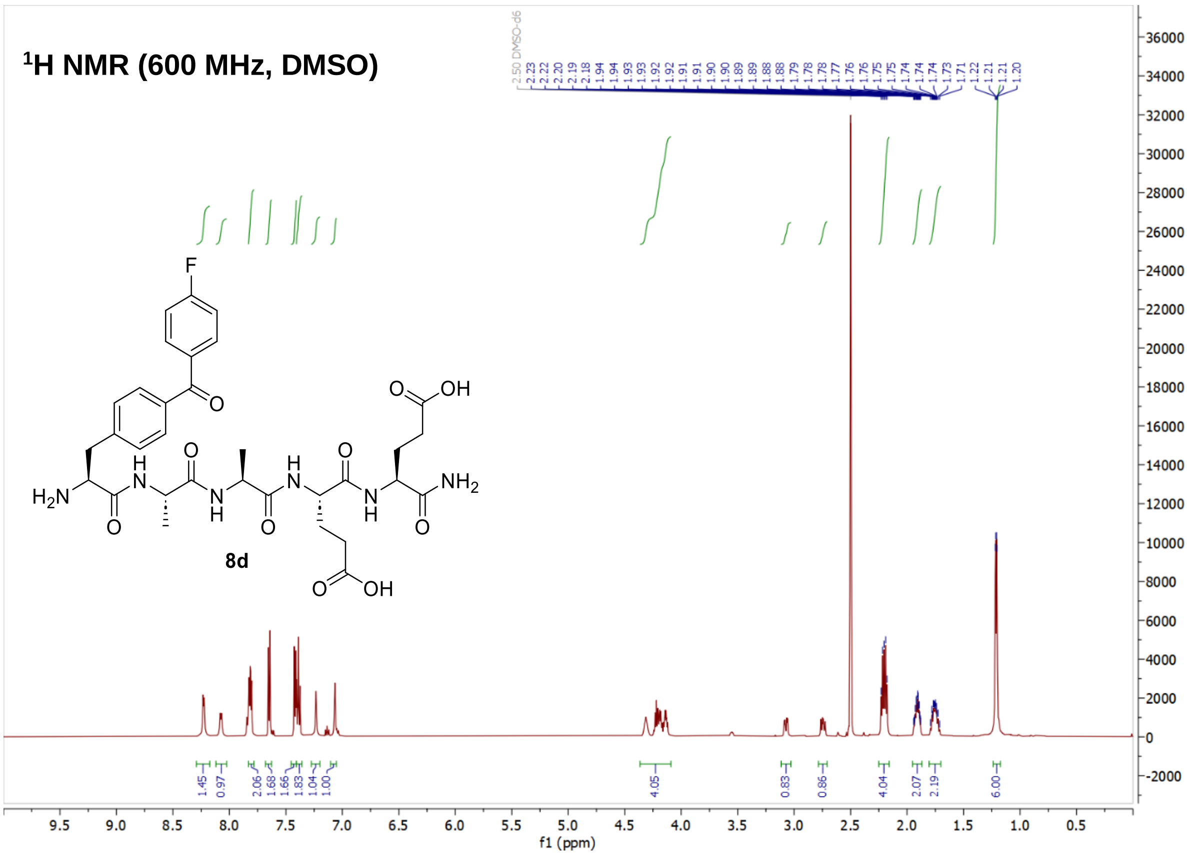

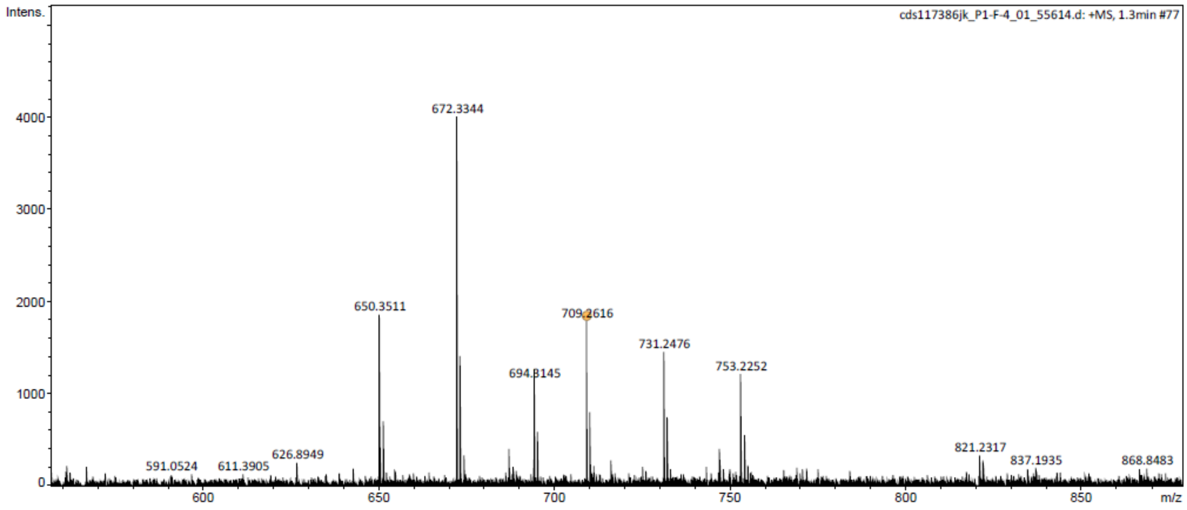

Meas. m/z	#	Ion Formula	m/z	err [ppm]	err [mDa]	mSigma	Mean err [ppm]
709.2616	1	C32H39FN6NaO10	709.2604	-1.7	-1.2	38.9	-3.7

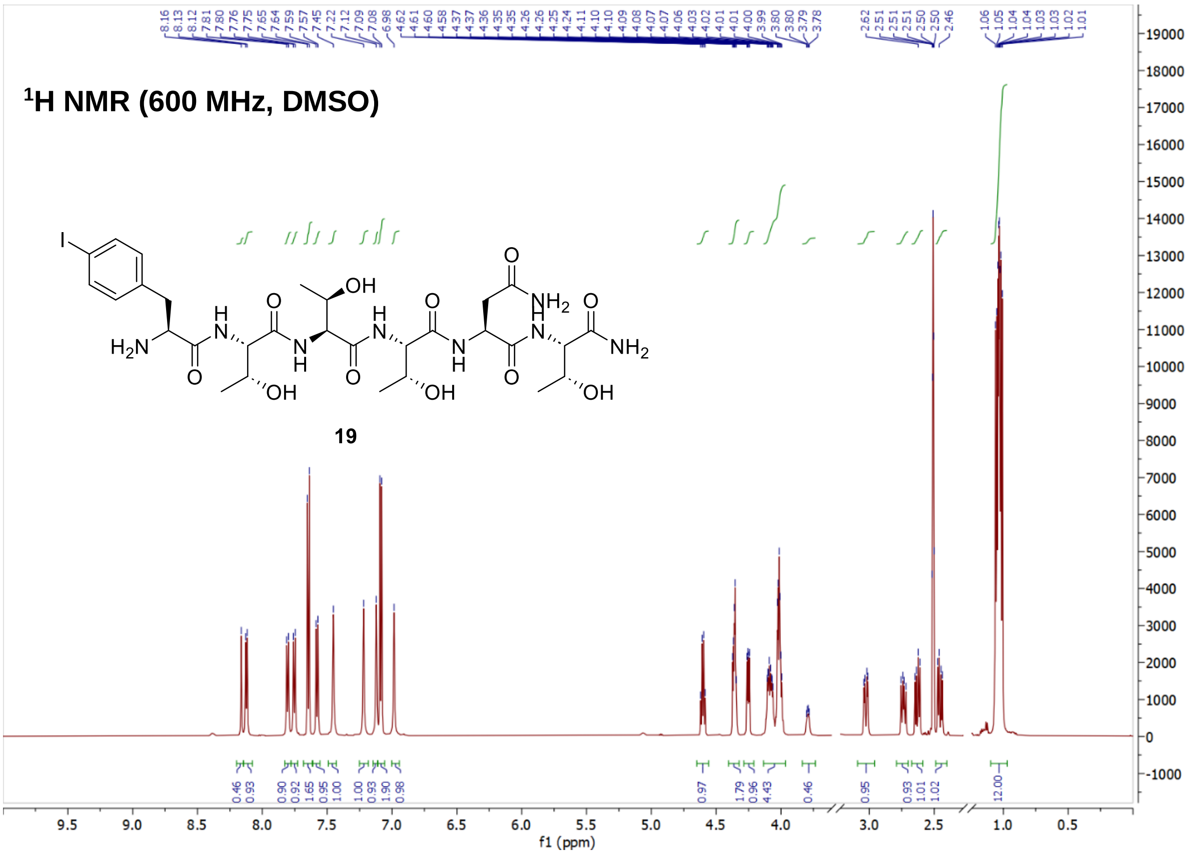

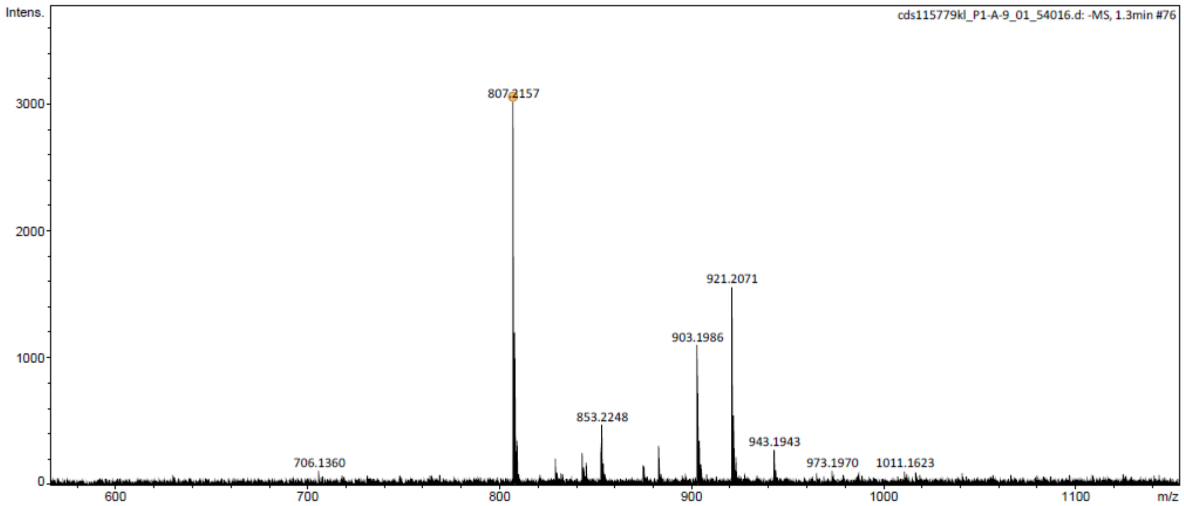

Meas. m/z	#	Ion Formula	m/z	err [ppm]	err [mDa]	mSigma	Mean err [ppm]
807.2157	1	C29H44IN8O11	807.2180	2.8	2.2	28.8	2.1

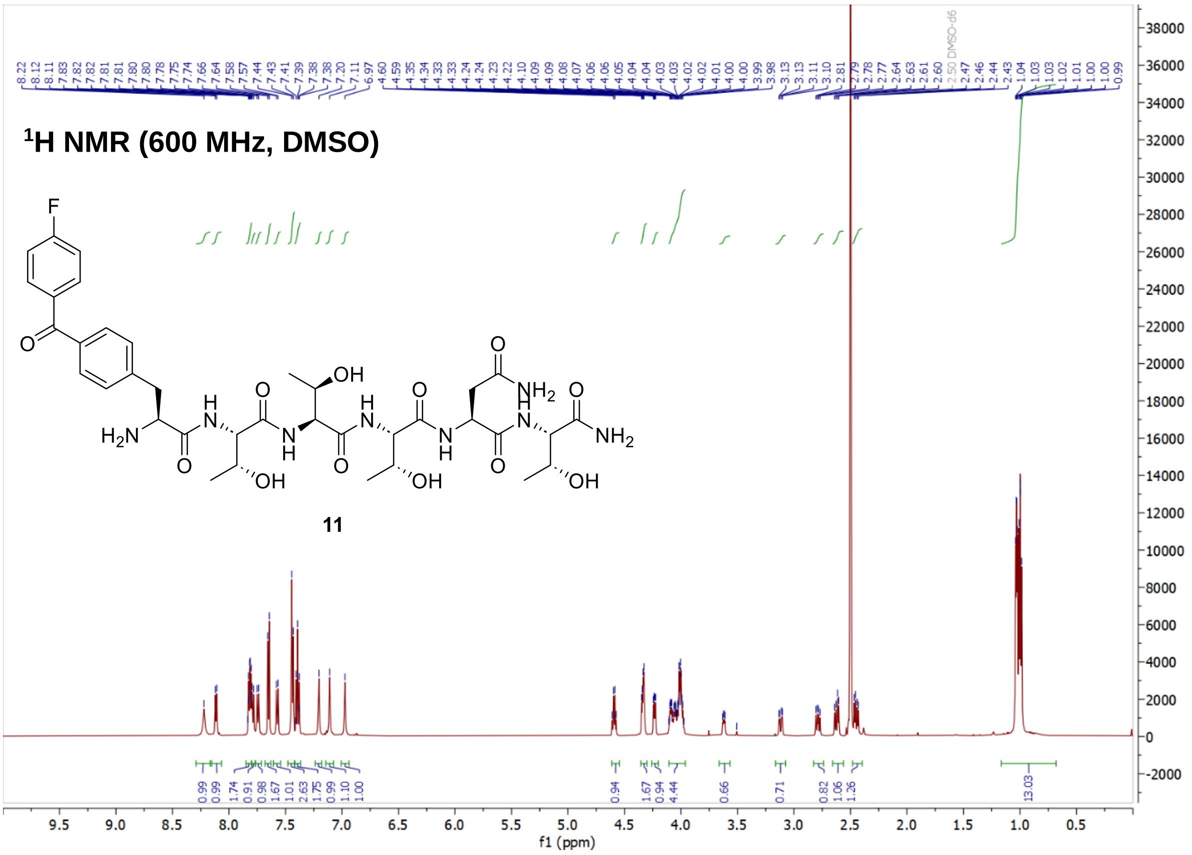

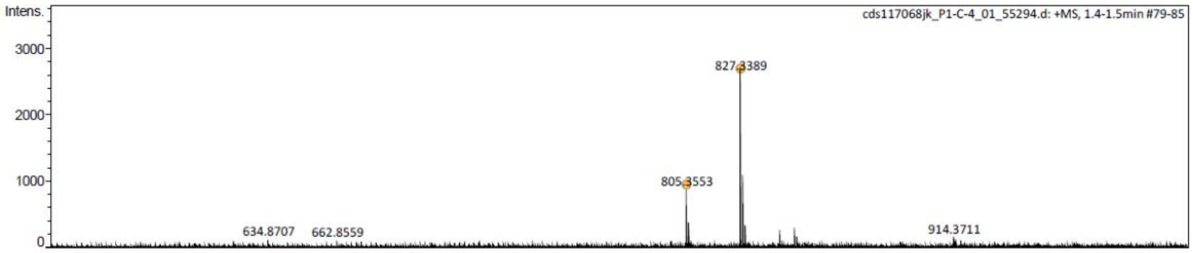

Meas. m/z	#	Ion Formula	m/z	err [ppm]	err [mDa]	mSigma	Mean err [ppm]
805.3553	1	C36H50FN8O12	805.3527	-3.2	-2.6	11.7	-3.1
827.3389	1	C36H49FN8NaO12	827.3346	-5.2	-4.3	15.1	-3.9

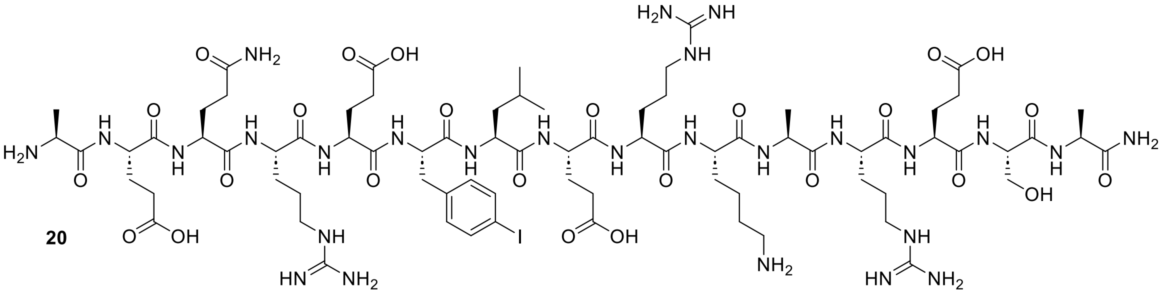

20

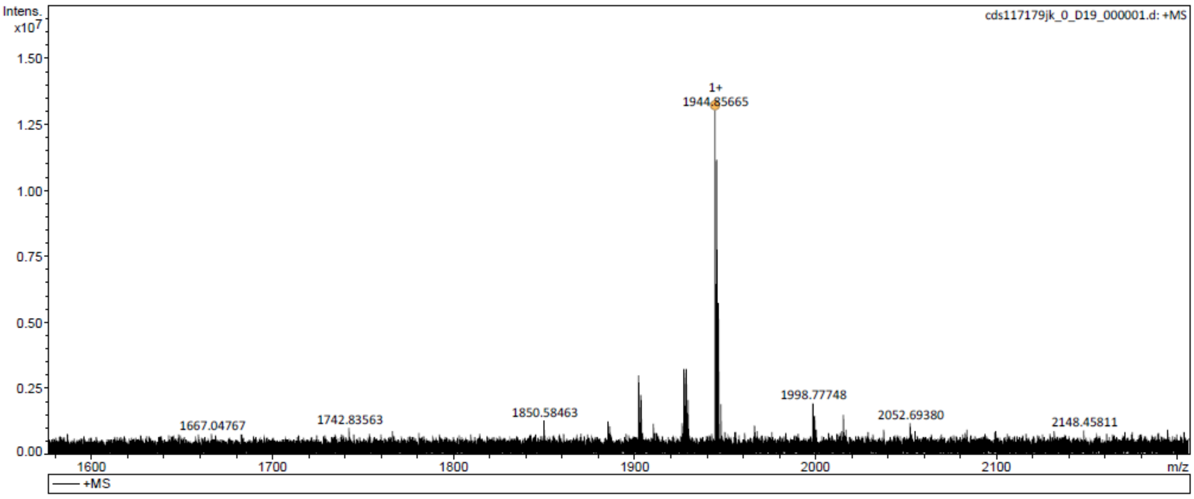

Meas. m/z	#	Ion Formula	m/z	err [ppm]	err [mDa]	mSigma	Mean err [ppm]
1944.856652	1	C76H127IN27O25	1944.853567	-1.6	-3.1	49.2	-1.2

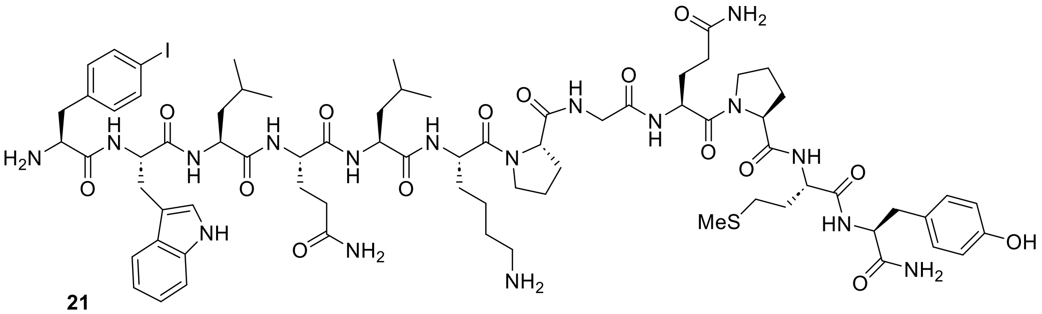

21

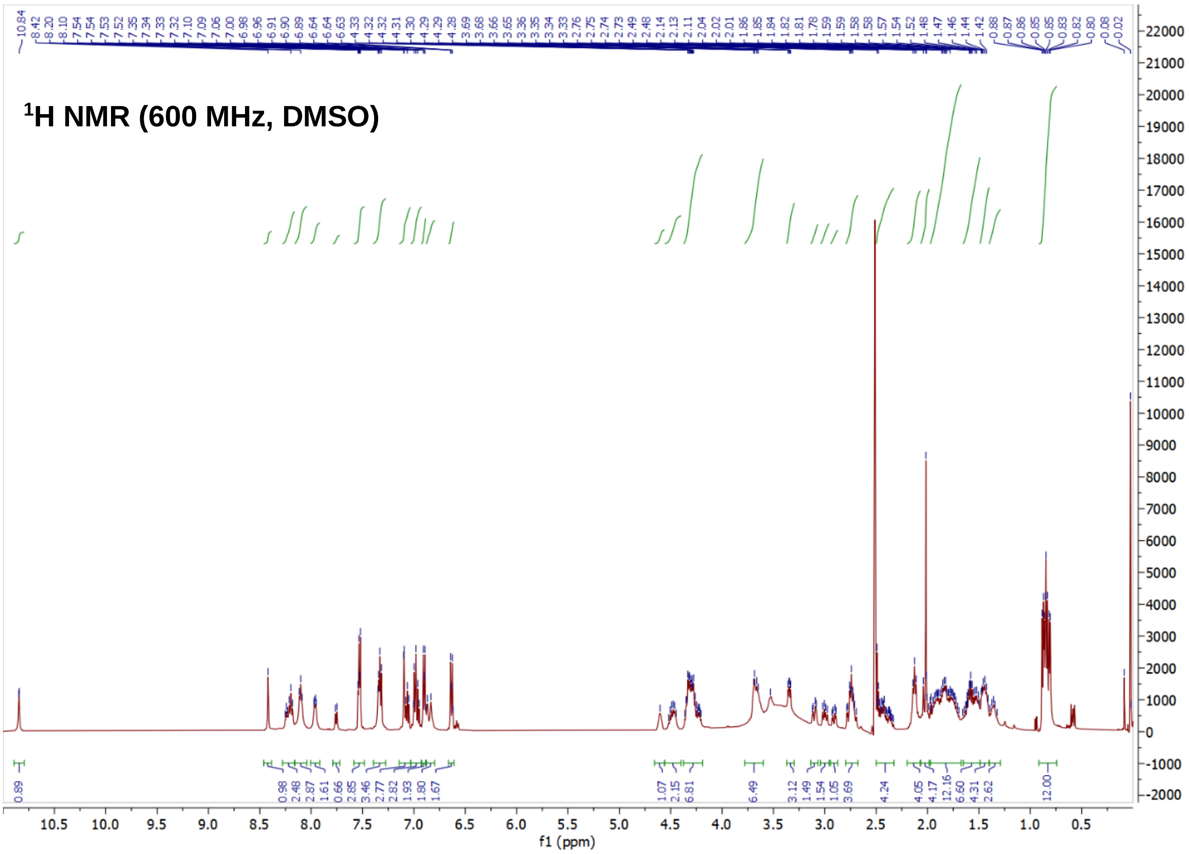

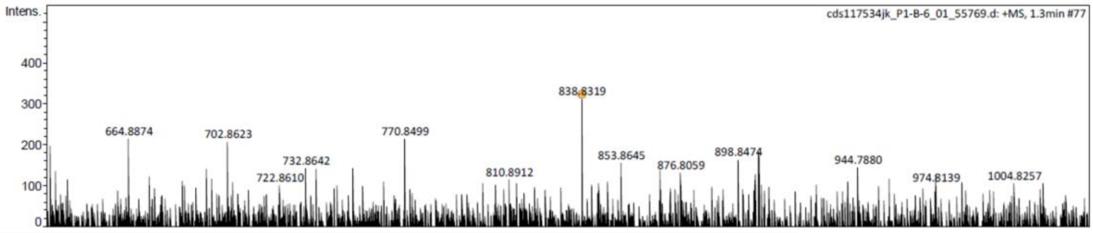

Meas. m/z	#	Ion Formula	m/z	err [ppm]	err [mDa]	mSigma	Mean err [ppm]
838.8319	1	C74H106IN17Na2O15S	838.8302	-2.0	-1.7	n.a.	-0.5

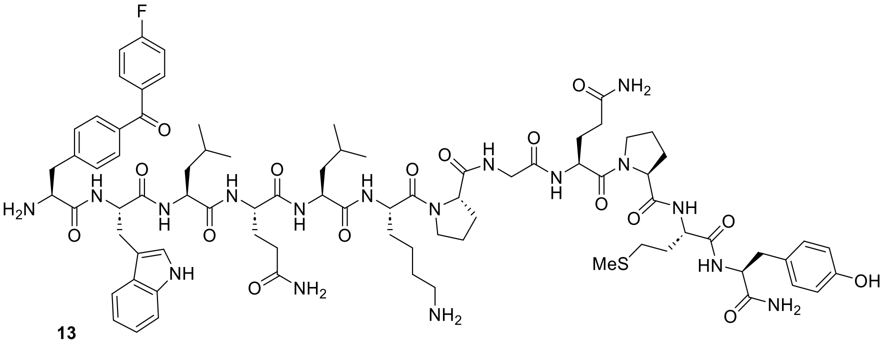

13

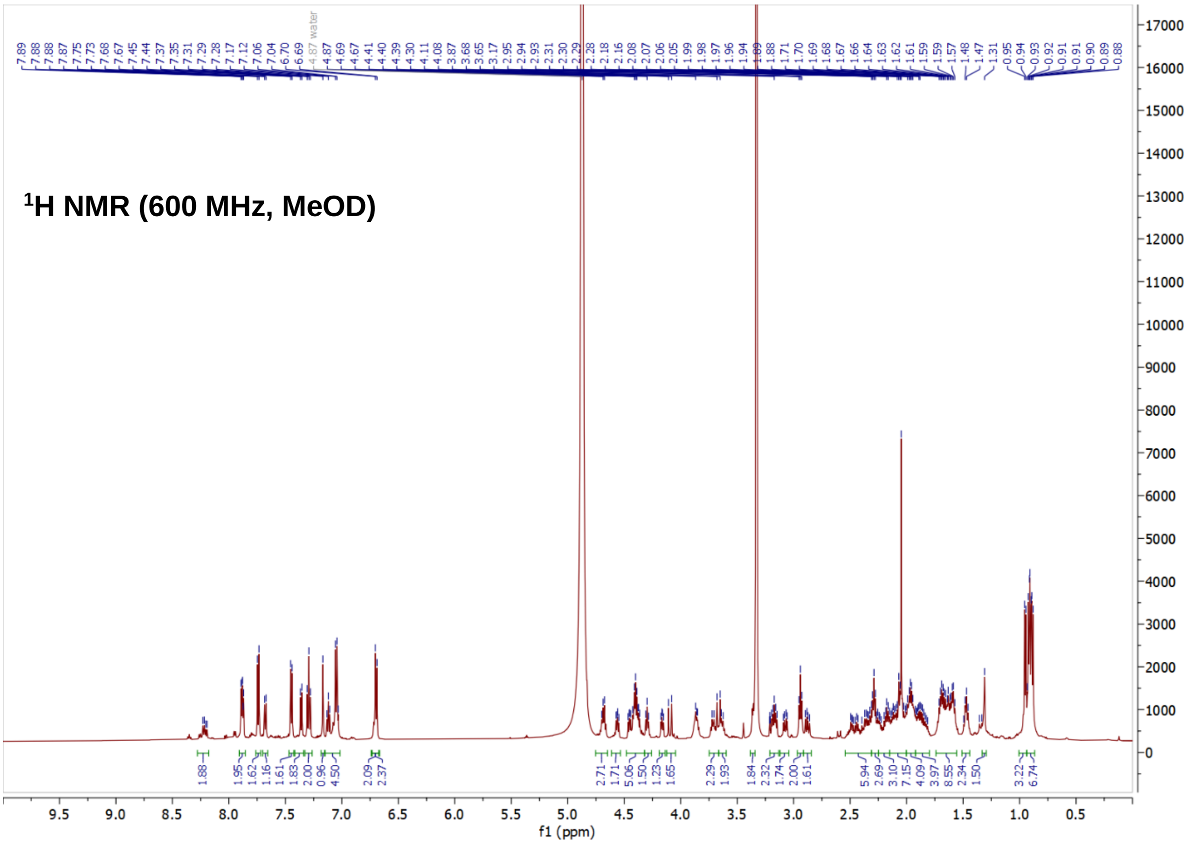

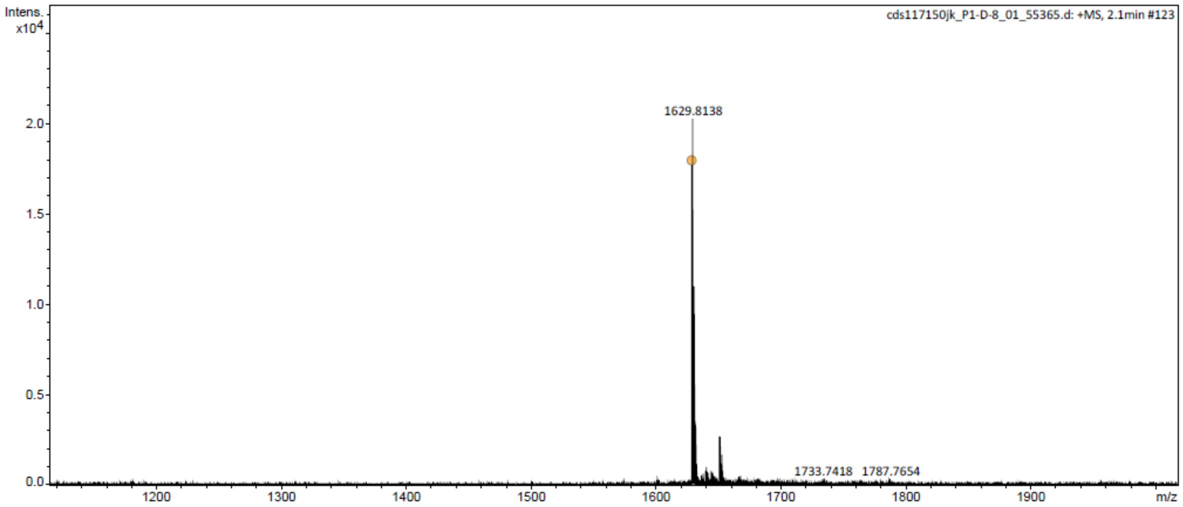

Meas. m/z	#	Ion Formula	m/z	err [ppm]	err [mDa]	mSigma	Mean err [ppm]
1628.8117	1	C81H111FN17O16S	1628.8094	-1.4	-2.3	58.0	-1.2

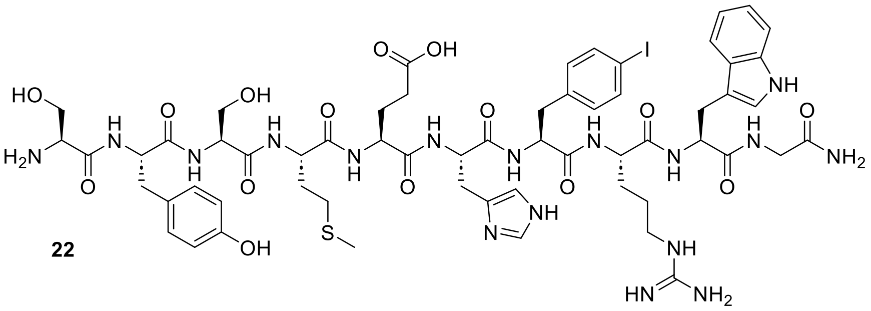

22

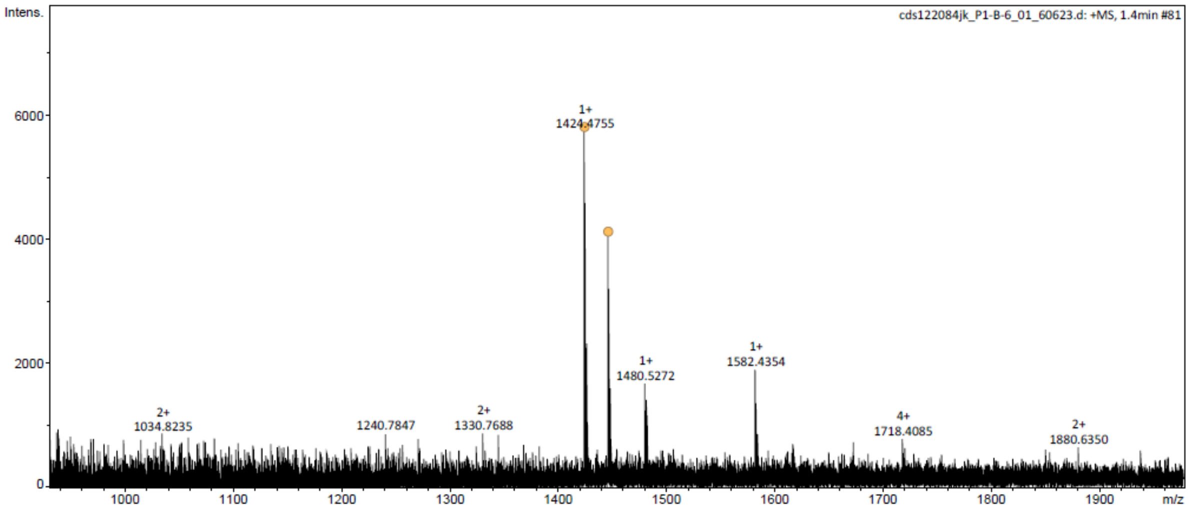

Meas. m/z	#	Ion Formula	m/z	err [ppm]	err [mDa]	mSigma	Mean err [ppm]
1424.4755	1	C59H79IN17O15S	1424.4701	-3.8	-5.4	53.0	-2.9
1446.4543	1	C59H78IN17NaO15S	1446.4521	-1.5	-2.2	58.0	-1.5

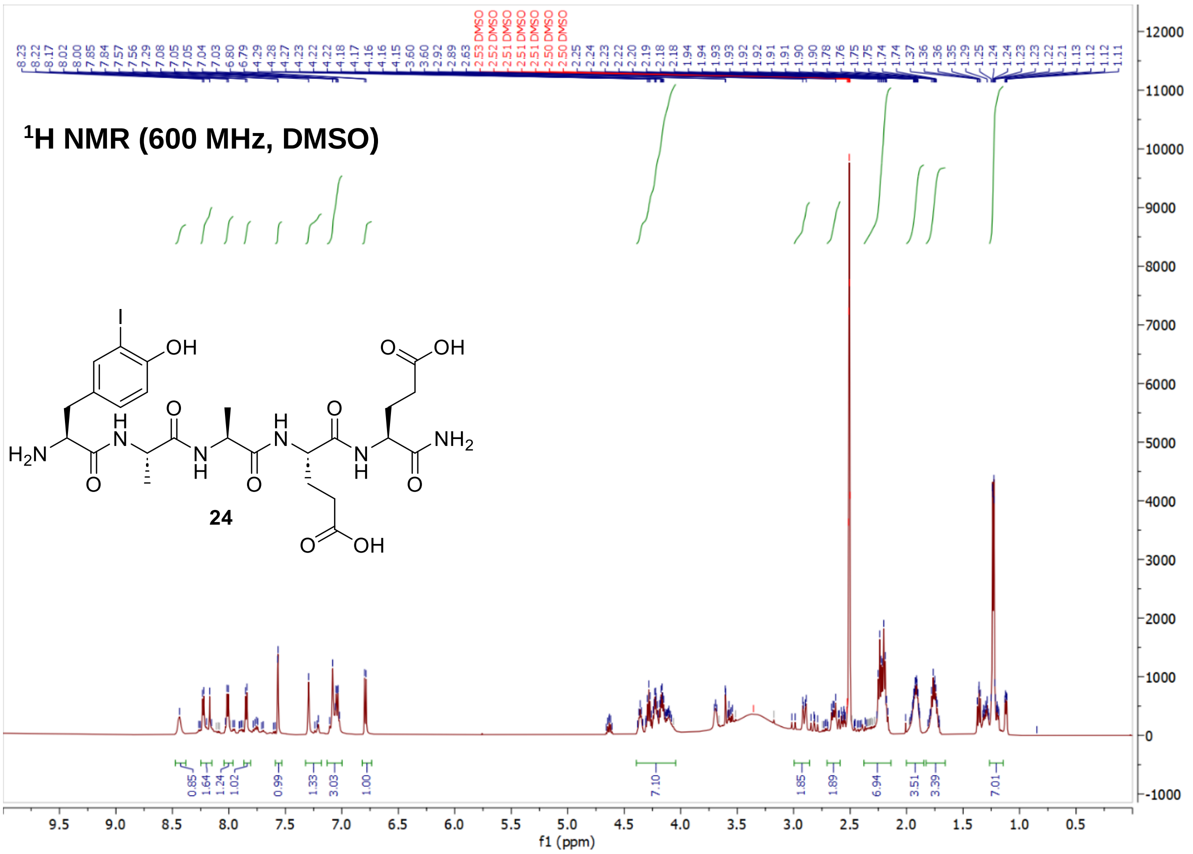

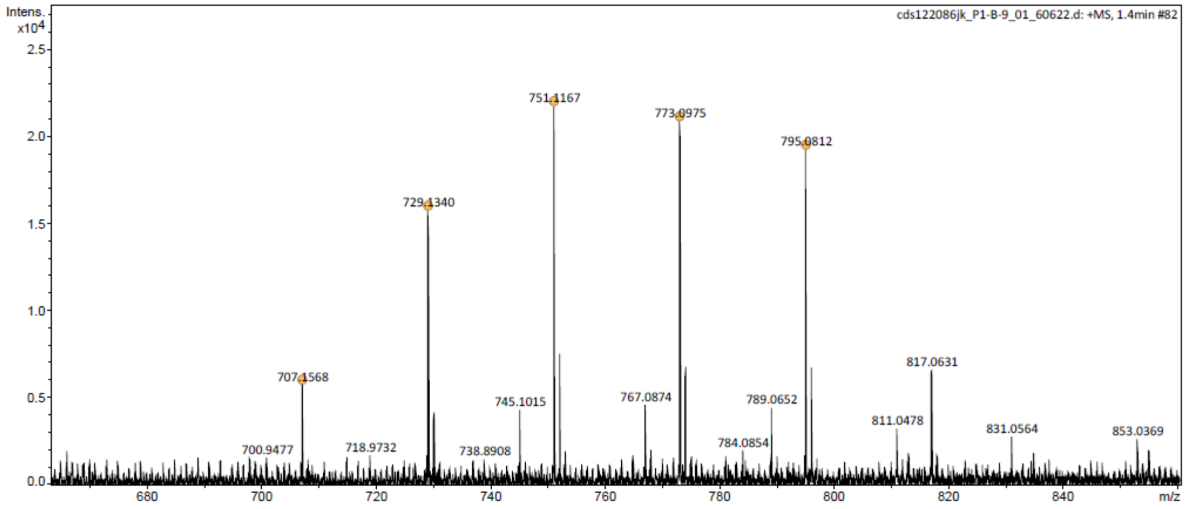

Meas. m/z	#	Ion Formula	m/z	err [ppm]	err [mDa]	mSigma	Mean err [ppm]
707.1568	1	C25H36IN6O10	707.1532	-5.0	-3.6	38.9	-2.7
729.1340	1	C25H35IN6NaO10	729.1352	1.5	1.1	19.6	0.9
751.1167	1	C25H34IN6Na2O10	751.1171	0.6	0.4	26.9	0.5
773.0975	1	C25H33IN6Na3O10	773.0990	2.0	1.5	15.0	2.4
795.0812	1	C25H32IN6Na4O10	795.0810	-0.3	-0.3	27.5	0.3

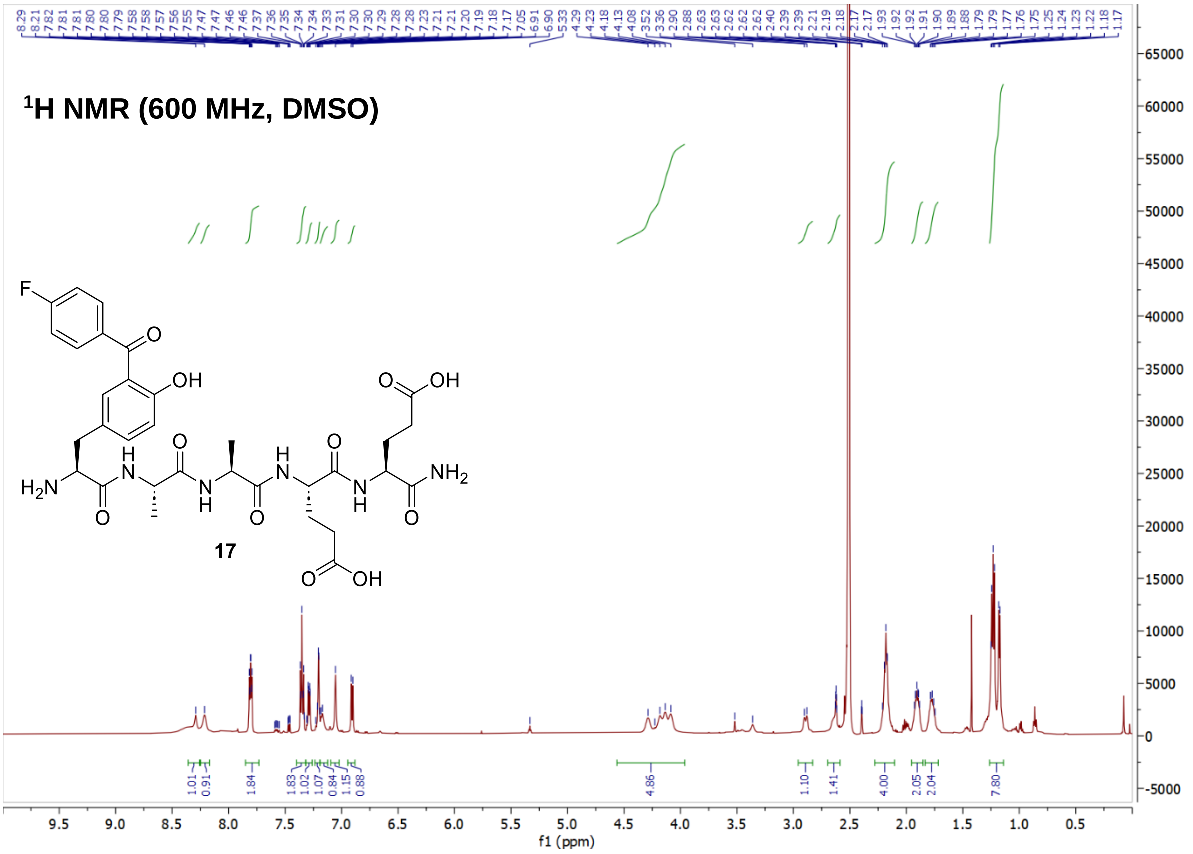

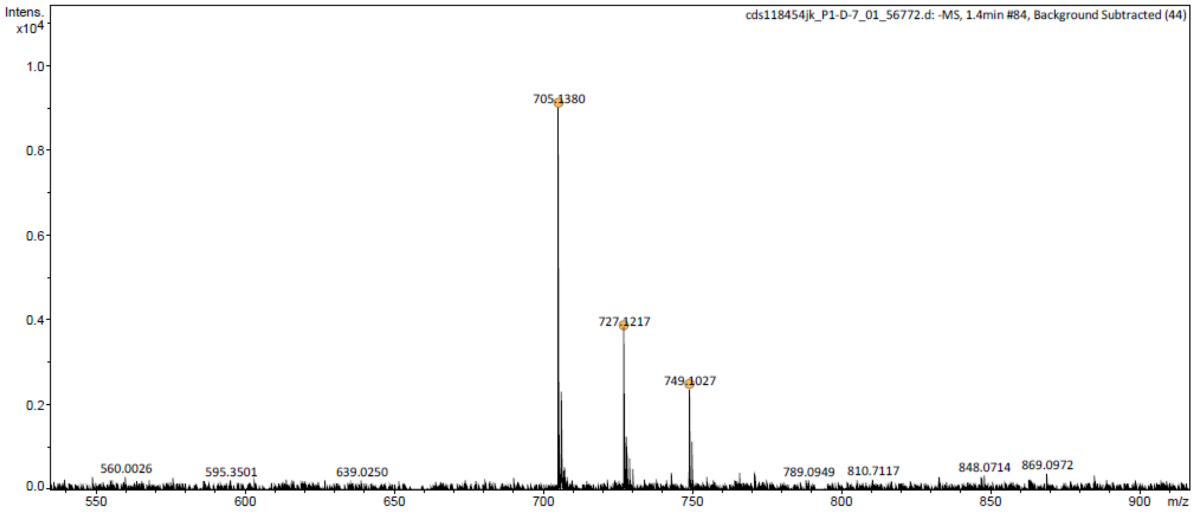

Meas. m/z	#	Ion Formula	m/z	err [ppm]	err [mDa]	mSigma	Mean err [ppm]
705.1380	1	C25H34IN6O10	705.1387	0.9	0.6	39.1	1.2
727.1217	1	C25H33IN6NaO10	727.1206	-1.5	-1.1	164.1	-2.5
749.1027	1	C25H32IN6Na2O10	749.1026	-0.3	-0.2	94.2	2.6

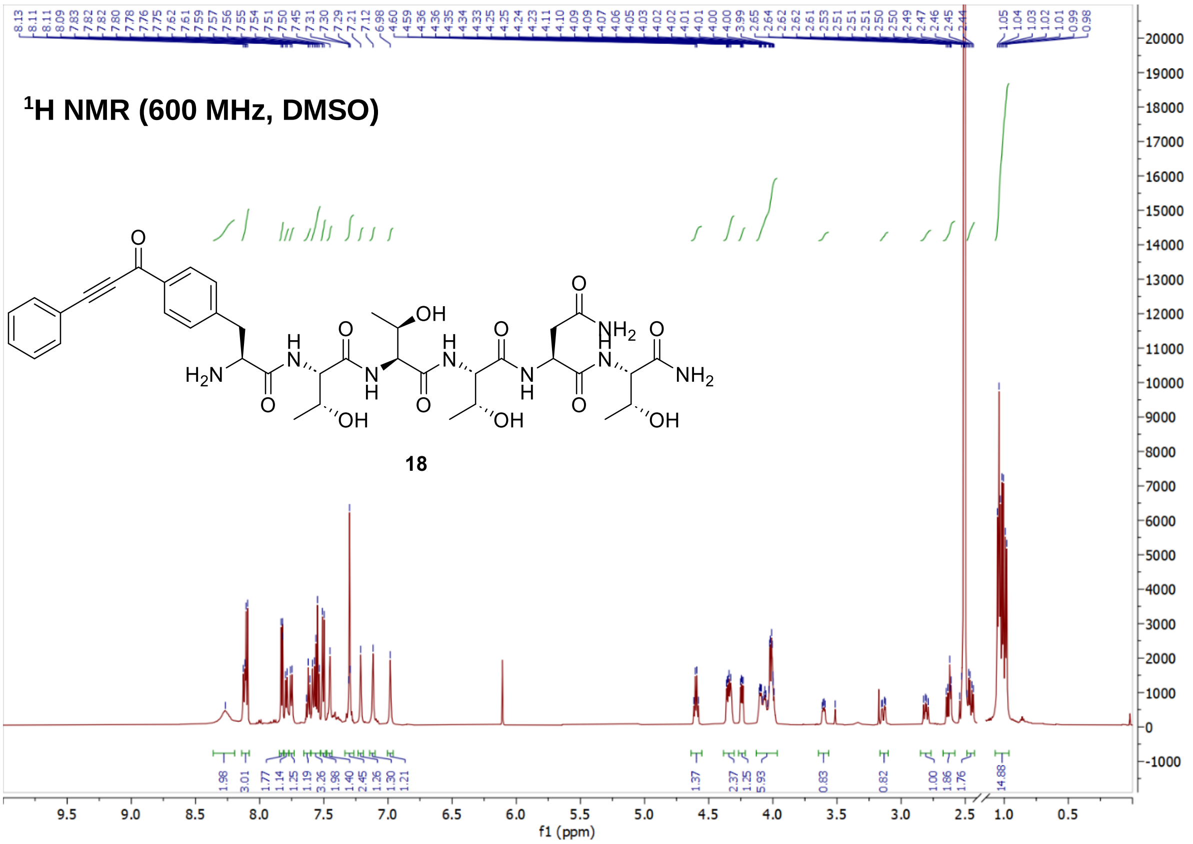

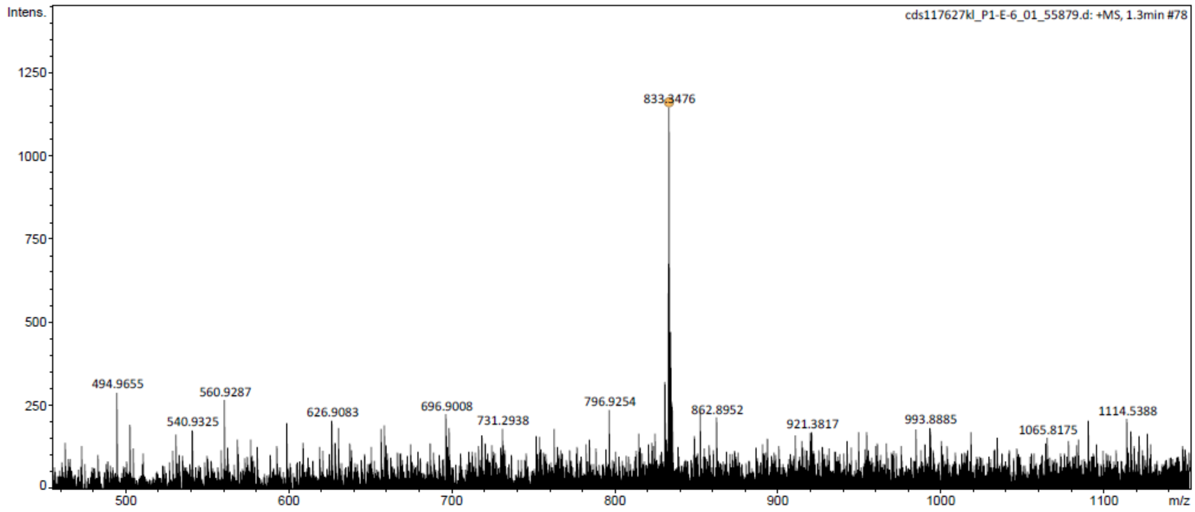

Meas. m/z	#	Ion Formula	m/z	err [ppm]	err [mDa]	mSigma	Mean err [ppm]
833.3476	1	C38H50N8NaO12	833.3440	-4.2	-3.5	66.1	-3.9

11. Small molecule NMRs and HRMS

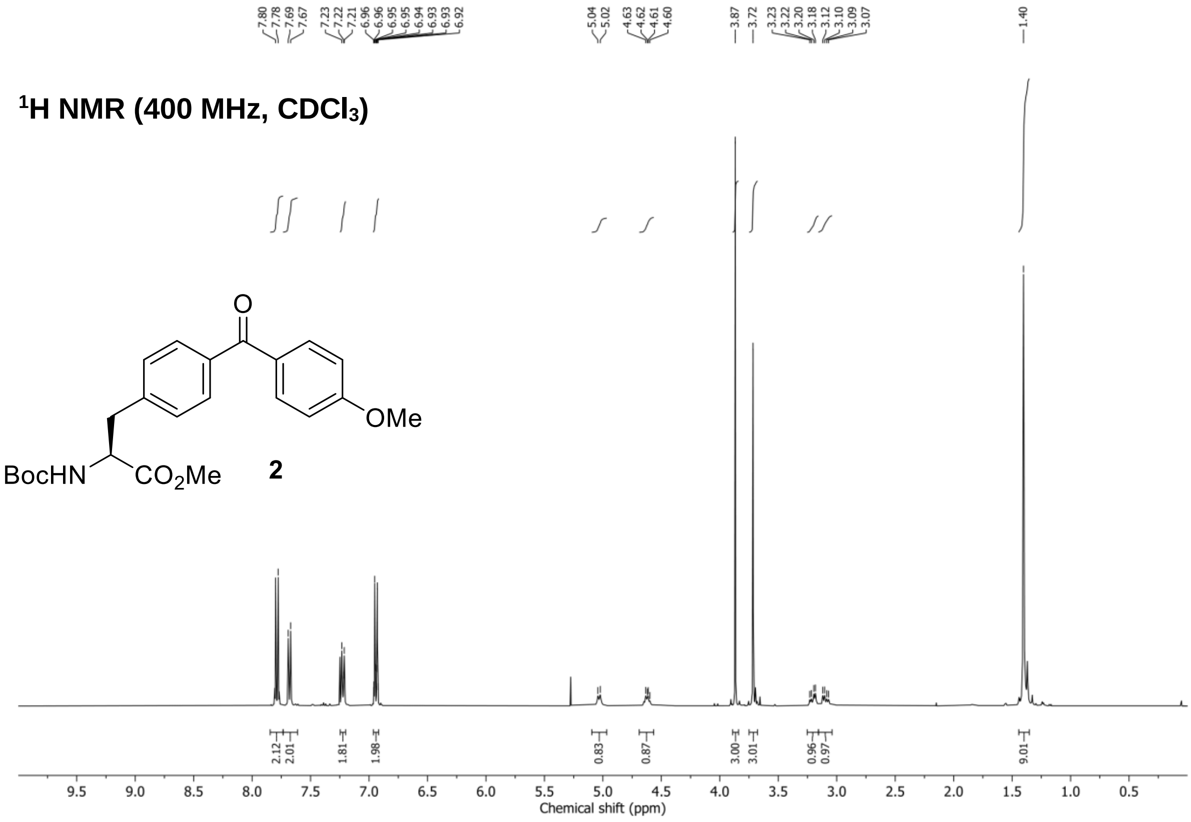

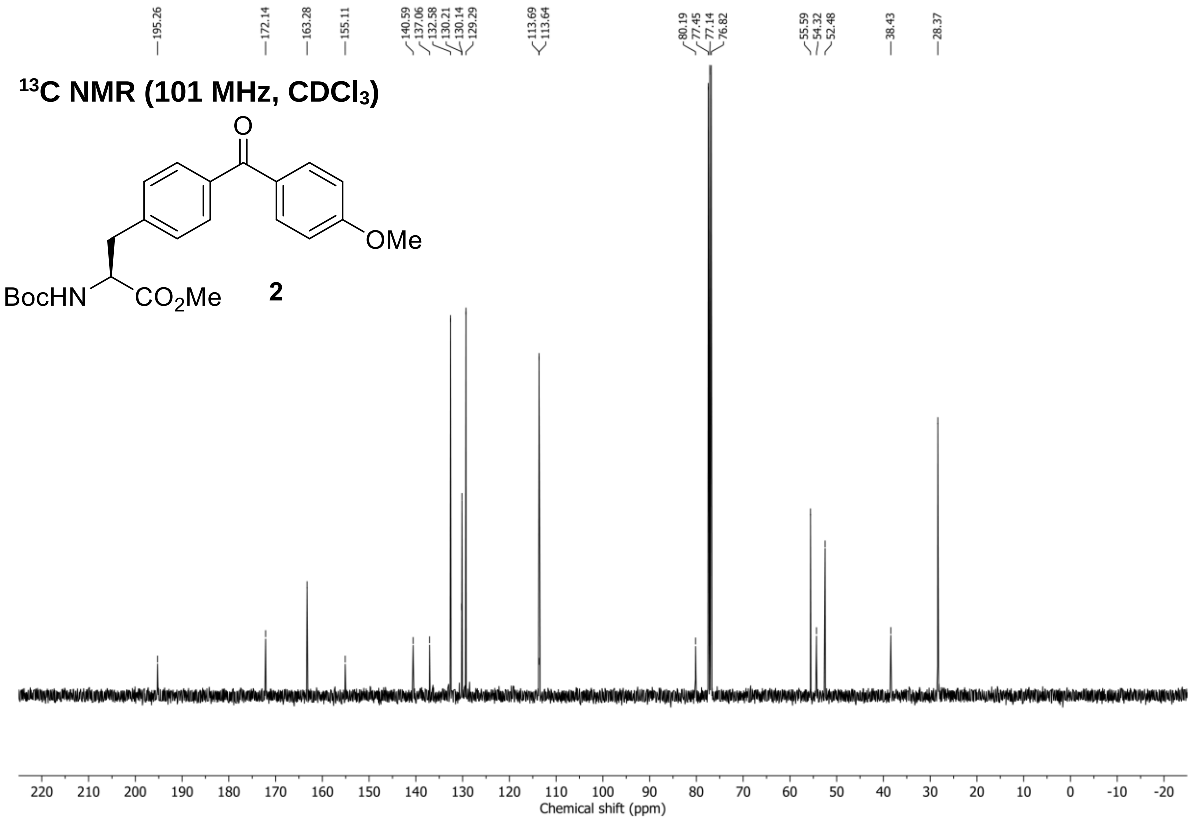

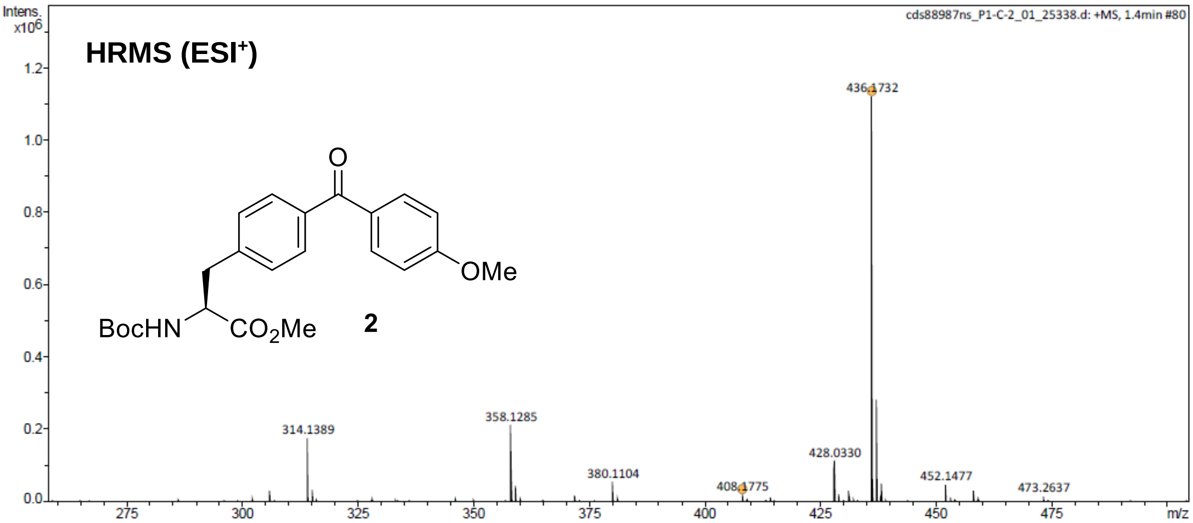

Meas. m/z	#	Ion Formula	m/z	err [ppm]	err [mDa]	mSigma	Mean err [ppm]
408.1775	1	C22H27NNaO5	408.1781	1.6	0.7	26.4	0.5
436.1732	1	C23H27NNaO6	436.1731	-0.3	-0.2	3.0	-0.6

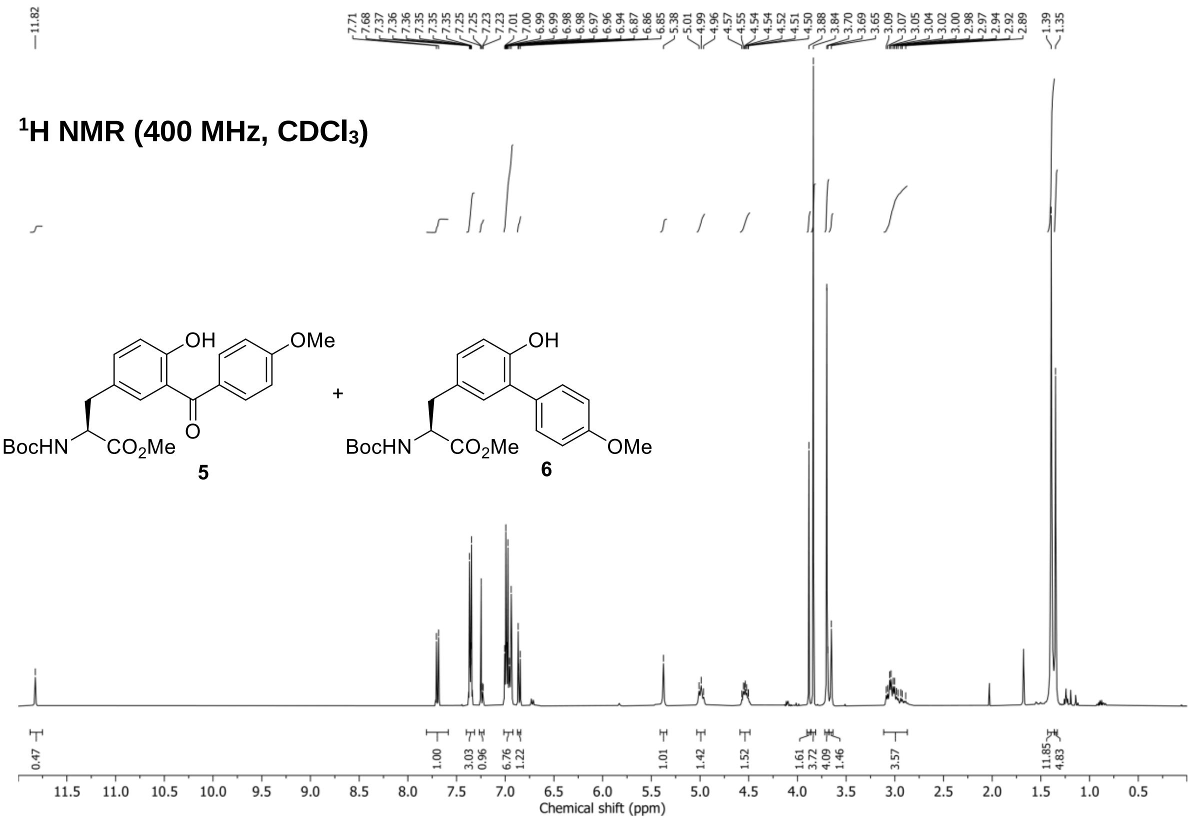
1H NMR (400 MHz, CDCl3)
OH
OMe
O
BocHN
CO2Me
5
+
OH
BocHN
CO2Me
OMe
6
Chemical shift (ppm)

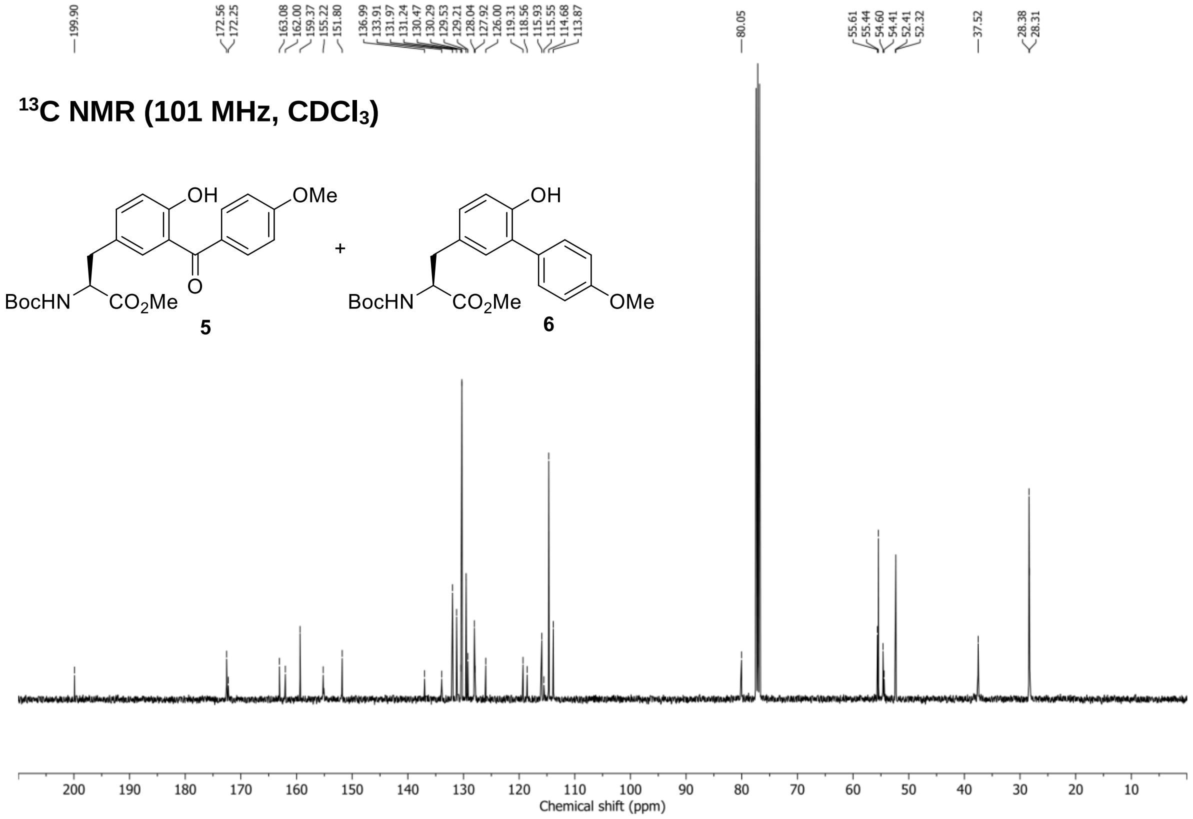
13C NMR (101 MHz, CDCl3)
OH
OMe
O
BocHN
CO2Me
5
+
OH
BocHN
CO2Me
OMe
6
Chemical shift (ppm)

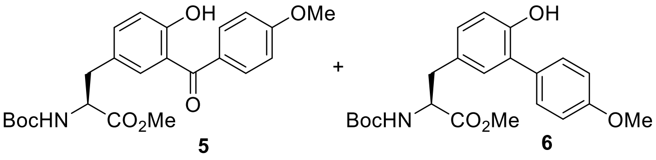

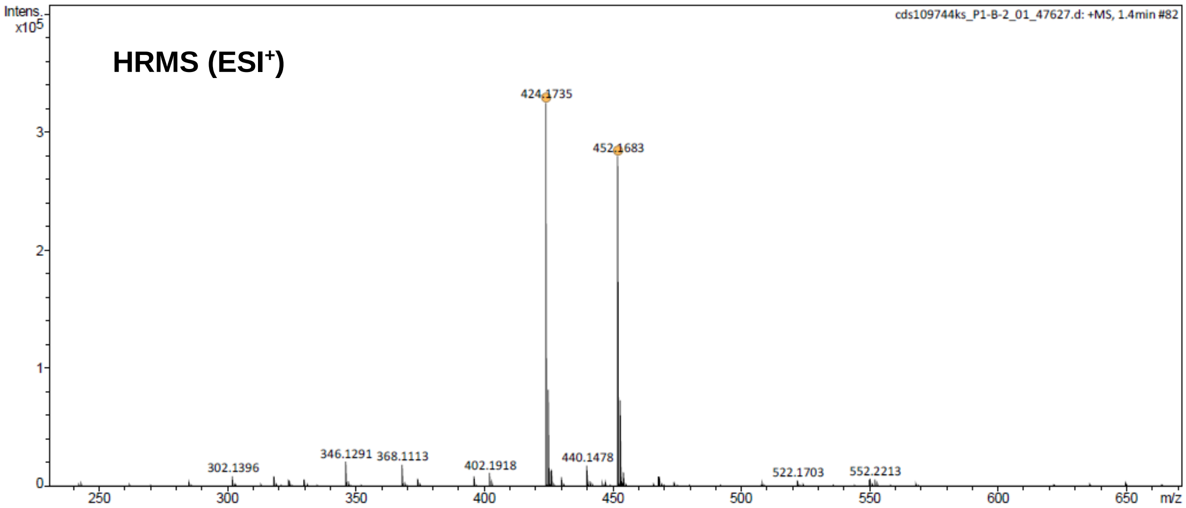

Meas. m/z	#	Ion Formula	m/z	err [ppm]	err [mDa]	mSigma	Mean err [ppm]
424.1735	1	C22H27NNaO6	424.1731	-1.1	-0.5	2.5	-1.3
452.1683	1	C23H27NNaO7	452.1680	-0.8	-0.4	2.0	-1.7
